# Structure-based inhibitor design for reshaping bacterial morphology

**DOI:** 10.1038/s42003-022-03355-3

**Published:** 2022-04-28

**Authors:** Yuri Choi, Ji Su Park, Jinshil Kim, Kyungjin Min, Kiran Mahasenan, Choon Kim, Hye-Jin Yoon, Sewon Lim, Dae Hee Cheon, Yan Lee, Sangryeol Ryu, Shahriar Mobashery, B. Moon Kim, Hyung Ho Lee

**Affiliations:** 1 Department of Chemistry, College of Natural Sciences, Seoul National University, Seoul, 08826 Korea; 2 Department of Food and Animal Biotechnology, Department of Agricultural Biotechnology, Research Institute for Agriculture and Life Sciences, Seoul National University, Seoul, 08826 Korea; 3 Center for Food and Bioconvergence, Seoul National University, Seoul, 08826 Korea; 4 Department of Chemistry and Biochemistry, University of Notre Dame, Notre Dame, Indiana 46556 United States

**Keywords:** X-ray crystallography, X-ray crystallography

## Abstract

The spiral shape of intestinal pathogen *Campylobacter jejuni* is critical for invasion of intestinal mucosa epithelial cells. Insofar as this cell morphology plays a role in the pathology of *C. jejuni* infection, its restructuring by pharmacological intervention could be an unexplored means to prevention of infection. We recently described that peptidoglycan hydrolase 3 (Pgp3) is involved in the spiral-shape formation of *C. jejuni*. We report herein the design and synthesis of the hydroxamate-based inhibitors targeting Pgp3. *C. jejuni* cells exposed to these inhibitors changed from the helical- to rod-shaped morphology, comparable to the case of the *pgp3*-deletion mutant. Evidence for the mechanism of action was provided by crystal structures of Pgp3 in complex with inhibitors, shedding light into the binding modes of inhibitors within the active site, supported by kinetics and molecular-dynamics simulations. *C. jejuni* exposed to these inhibitors underwent the morphological change from helical- to rod-shaped bacteria, an event that reduce the ability for invasion of the host cells. This proof of concept suggests that alteration of morphology affects the interference with the bacterial infection.

## Abstract (web summary)

Peptidoglycan hydrolase 3 (Pgp3) inhibitors are designed that can change the helical morphology of *Campylobacter jejuni* to rod-shaped and decrease its virulence.

## Introduction

The rapid emergence of antibiotic-resistance bacteria is creating a global crisis. The problem is multifaceted, attributed to poor stewardship of antibiotics as well as a lack of new drugs with novel mechanisms of action^1^. Notwithstanding the genuine need, no new classes of antibiotics have been developed for decades for treatment of infections by Gram-negative bacteria^2^. In this regard, targeting cell morphological determinants have been emerged as a new antibacterial strategy, because accumulating evidence supports critical roles of bacterial morphology in bacteria-host interactions, including pathogenesis^3^. Herein, we investigated inhibitors transforming the cell morphology of *Campylobacter jejuni* as an anti-virulence strategy. *C. jejuni*, a Gram-negative bacterial pathogen, is one of the leading global causes of bacterial gastroenteritis worldwide. Infections by *C. jejuni* trigger severe complications such as inflammatory bowel disease, reactive arthritis, and the Guillain-Barré syndrome^4,5^. This pathogen has become increasingly antibiotic-resistant in recent years^6–10^, and especially resistance to fluoroquinolones and macrolides has been rampant^10–12^.

For colonization and for penetration into host cells, motility of *C. jejuni* through the viscous mucus layer of the gastrointestinal tract is important^13^. Interestingly, the helical shape of *C. jejuni* is crucial for the motility by a corkscrew-like motion^13^. In fact, non-motile strains are severely impaired in their ability to colonize the host intestines^13–16^, suggesting that abrogation of the helical cell shape could interfere with the bacterial lifestyle and virulence. The specific type of crosslinking of the cell-wall peptidoglycan in *C. jejuni* is believed to be responsible for its helical shape^17,18^. A polysaccharide chain consisting of repeating β(1 → 4)-linked *N*-acetylglucosamine (GlcNAc or NAG)-*N*-acetylmuramic acid (MurNAc or NAM) disaccharide unit comprises the basic unit of *C. jejuni* peptidoglycan and a pentapeptide (l-Ala^1^-*γ*-d-Glu^2^-*m*DAP^3^-d-Ala^4^-d-Ala^5^, where *m*DAP refers to *meso*-2,6-diaminopimelate) is attached to the NAM^16^. The 4 → 3 amide linkage between d-Ala^4^ from one strand and *m*DAP^3^ from another strand are involved in crosslinking the neighboring peptidoglycan strands^17,19–21^.

**Fig. 1:**
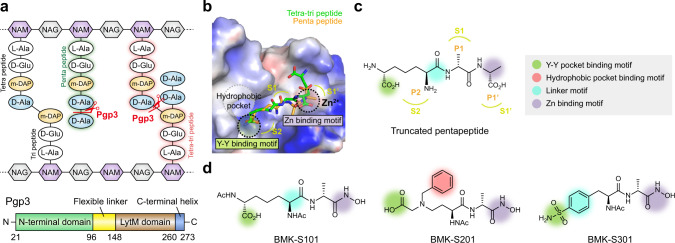
Structure-based design of Pgp3 inhibitors. **a** Schematic diagram of peptidoglycan structure and domain architecture of Pgp3. Cleavage sites by Pgp3 are shown with scissors. **b** Surface representation of the electrostatic potential of Pgp3 in complex with tetra-tripeptide and pentapeptide (PDB ID: 6JN0 and 6JN1, respectively). Tetra-tripeptide and pentapeptide are shown in green and orange sticks, respectively. Representative motifs are shown in dashed circles. Substrate binding pocket (S1’, S1, and S2) are colored in lime. **c** Chemical structure of truncated pentapeptide (mDAP^3^-D-Ala^4^-D-Ala^5^). Green, red, purple, and cyan circles indicate the Y–Y binding motif, hydrophobic pocket binding motif, Zn binding motif, and linker motif, respectively. Substrate-specific binding residues (P1’, P1, and P2) and substrate binding pockets (S1’, S1, and S2) are colored in orange and lime, respectively. **d** Chemical structure of synthesized inhibitors. BMK-S101 was designed in keeping of Zn binding motif, linker motif, and Y–Y binding motif based on pentapeptide. In BMK-S201, additional binding in hydrophobic pocket was introduced, whereas the rigidity was modulated in BMK-S301 by inserting the linker motif.

*C. jejuni* harbors several additional genes encoding peptidoglycan peptidases, which hydrolyze specific sites of the peptidoglycan to conform to the helical shape by yet unknown mechanism^22,23^. The peptidases discovered in *C. jejuni* were peptidoglycan peptidase 1 and 2 (Pgp1 and Pgp2), which are responsible for cleaving peptidoglycan stem peptides by their carboxypeptidase activities^24,25^. Subsequently, we identified another gene, the peptidoglycan peptidase 3 (Pgp3) existing both d,d-endopeptidase and d,d-carboxypeptidase activities^26^. Crystal structures of *C. jejuni* Pgp3 in complex with tetra-tri peptide and pentapeptide derived from peptidoglycan revealed the molecular basis of peptidoglycan turnover chemistry^26^. The Pgp3 LytM domain is responsible for the substrate binding and contains a highly conserved tetrahedral Zn^2+^-ion-binding motif (Fig. 1a)^26^. The *pgp3*-deletion mutant exhibited a curved-rod appearance with a significant deviation from the helical morphology and substantially decreased invasive ability compared to the wild-type strain. These observations are consistent with Pgp3 playing an important role in maintenance of *C. jejuni* helical shape and, by extension, its virulence^26^.

The knowledge of the crystal structure of Pgp3 provided an opportunity for design of inhibitors targeting this enzyme as a potential pharmacological intervention in *C. jejuni* infections. The design, synthesis and evaluation of seven inhibitors is described in this study. *C. jejuni* strains treated with the inhibitors altered their shape from the helical to rod morphology. The inhibitors bound to the enzyme were assessed with seven crystal structures, which shed light on the mechanism of action.

## Results

### Design and synthesis of BMK-S101 for Pgp3

As the crystal structure of Pgp3 revealed the LytM domain contains a Zn^2+^ ion withinthe catalytic site, the main strategy was the design of inhibitors that would bind to the active site of LytM domain by metal coordination in a competitive manner (Fig. 1a). The first designed inhibitor, the compound designated BMK-S101, was conceived by mimicking Pgp3 substrates based on the crystal structures of a mutant variant of Pgp3 in complex with its substrates: a cross-linked muramyl tetra-tri peptide (NAM-l-Ala^1^-*γ*-d-Glu^2^-*m*DAP^3^-d-Ala^4^**−***m*DAP^3^-*γ*-d-Glu^2^-l-Ala^1^-NAM) and a pentapeptide (Ac-l-Ala^1^-*γ*-d-Glu^2^-*m*DAP^3^-d-Ala^4^**−**d-Ala^5^)^26^ (Fig. 1a). Both substrates contain the common motif, *m*DAP^3^-d-Ala^4^ (P2-P1), and only the fifth amino acid is different, i.e., *m*DAP^3^ vs d-Ala^5^. The *m*DAP^3^ side chain of the tetra-tri peptide encompasses the ‘d-Ala’ segment with the correct stereochemistry corresponding to d-Ala^5^ of pentapeptide. Based on the binding modes of tetra-tri peptide and pentapeptide, we reasoned that a hydroxamate moiety as a Zn^2+^-ion-binding motif could be incorporated. Thus, we used the *m*DAP^3^-d-Ala^4^ (P2-P1) backbone incorporated with the hydroxamate for BMK-S101 (Fig. 1c, d). We observed a hydrophobic pocket near the substrate- binding site, so we hypothesized that the incorporation of a phenyl moiety to interact with it would be beneficial (BMK-S201, Fig. 1b–d). Also, we postulated that the alkyl chain flexibility might affect binding affinity of inhibitor. Thus, we incorporated additional ring structures in the middle of BMK-S101 to increase the rigidity (BMK-S301, Fig. 1d).

### Structural analysis of Pgp3 in complex with BMK-S101

**Fig. 2:**
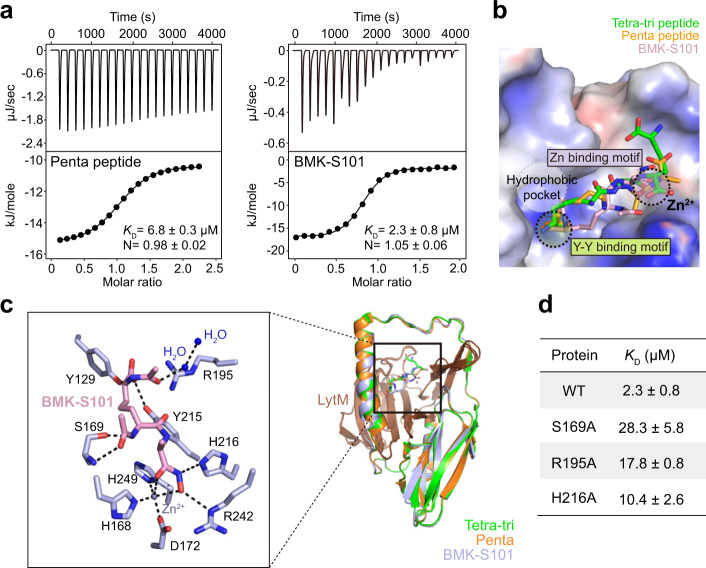
Structural and functional studies on Pgp3 in complex with BMK-S101. **a** Representative ITC fitting results of Pgp3 with the pentapeptide and BMK-S101. The thermodynamic data were collected from titration of compounds into Pgp3, and the parameters were calculated by fitting to a single-binding model. Due to cleavage, Pgp3 H247A variant was used for the pentapeptide. **b** Surface representation of the electrostatic potential of Pgp3 in complex with the tetra-tripeptide (green), the pentapeptide (orange), and BMK-S101 (light pink). Characteristic motifs are shown with dashed circles. Substrate binding pocket (S1’, S1, and S2) are colored in lime. **c** Superposition of three crystal structures of Pgp3 (H247A variant with the tetra-tripeptide in green, with the pentapeptide in orange, and with BMK-S101 in light pink). Detailed interaction of Pgp3 H247A active sites bound with BMK-S101 are expanded. BMK-S101 is shown in sticks colored with light pink, and key residues interacting in Pgp3 are colored with light blue. The coordination of Zn^2+^ and hydrogen bonds are represented by black dashed lines. Zn^2+^ ion and water molecules are shown in blue and slate gray spheres, respectively. **d** *K*_D_ values for Pgp3 WT or variants with BMK-S101. ITC fitting results are shown in Fig. 2a and Supplementary Fig. 1b–c.

We synthesized the BMK-S101 and assayed its ability to bind Pgp3 by isothermal-titration calorimetry (ITC). Indeed, BMK-S101 bound to Pgp3 with the *K*_D_ value of 2.3 ± 0.8 μM, ~3-fold better compared to the value for the pentapeptide (6.8 ± 0.3 μM) (Fig. 2a). In the case of the pentapeptide, the inactive Pgp3 H247A variant was used to prevent turnover of substrate.

We also solved the crystal structure of the Pgp3:BMK-S101 complex (Fig. 2b, c). Previously we had noted that the H247A variant of Pgp3 abrogates binding of tartrate or citrate at the active site^26^, which are essential components of the crystallization solutions. Thus, H247A variant was used for all structural studies. It adopts the open conformation for the active site, which the wild-type enzyme does as well^26^. Furthermore, the H247A variant exhibited a similar *K*_D_ value for BMK-S101 compared to the WT Pgp3 (Supplementary Fig. 1b). In the Pgp3-BMK-S101 complex, electron-density map was obtained for the inhibitor, but segments that were weak in electron density would appear to argue for mobility at those sites (Fig. 2c and Supplementary Fig. 2a). As Pgp3 adopts open and closed conformations due to the large conformational shift of the L1 loop^26^, we checked the conformation of the L1 loop in the BMK-S101-bound Pgp3. It showed the open conformation, similar to Pgp3 structures in complex with its substrates (Fig. 2c). When the structure of Pgp3 in complex with BMK-S101 was overlaid with that with the tetra-tri peptide, their overall structures exhibited high similarity, with RMSD of 0.17 Å over the 220 equivalent Cα positions (Fig. 2c).

BMK-S101 bound to the deep cleft of the LytM domain of Pgp3 with a 1:1 stoichiometry (Fig. 2c). When the electrostatic potentials are drawn at the surface of Pgp3, positive electrostatic potentials were widely distributed along the substrate-binding pocket (S1–S2), complemented by the negatively charged BMK-S101 (Fig. 2b). BMK-S101 mainly interacts with Pgp3 via its terminal *m*DAP and the hydroxamate moiety. Similar to the case of the peptide susbstrates, Tyr129 (at 2.8 Å), Tyr215 (at 2.7 Å) and Arg195 (at 3.4 Å) of Pgp3 contributed to the binding by forming several hydrogen-bonding networks (Fig. 2c). The hydroxamate group coordinated to Zn^2+^ (2.5 Å; Zn^2+^-O distance) and interacted with His168 (at 2.1 Å), Asp172 (at 2.2 Å), His249 (at 2.1 Å) and Arg242 (at 3.1 Å). The hydroxamate nitrogen interacts with His216 (at 3.0 Å). In the middle part of BMK-S101, N1 atom of BMK-S101 is hydrogen-bonded to Ser169 (at 2.4 Å). To evaluate the contributions of the three residues (Ser169, Arg195, and His216) to binding of BMK-S101, they were substituted to Ala, and the effect of each mutation was evaluated using ITC (Fig. 2d and Supplementary Fig. 1). As expected, *K*_D_ values of the mutant Pgp3 proteins increased at least 5-fold compared to that of WT Pgp3 (Fig. 2d and Supplementary Fig. 1b, c).

### Structural studies of BMK-S201 and its derivatives

**Fig. 3:**
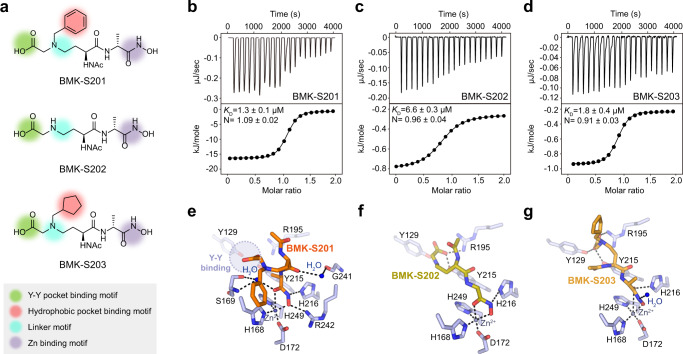
Structural and functional studies on Pgp3 in complex with BMK-S201 and its derivatives. **a** Chemical structures of BMK-S201 and its derivatives, BMK-S202 and BMK-S203. Green, red, purple, and cyan circles indicate the Y-Y binding motif, hydrophobic pocket binding motif, Zn binding motif, and linker motif, respectively. **b**–**d** The ITC fitting results of Pgp3 with (**b**) BMK-S201, (**c**) BMK-S202, and (**d**) BMK-S203. The thermodynamic data were collected from titration of each inhibitor into Pgp3, and the parameters were calculated by fitting to a single-binding model. **e**–**g** Detailed interaction of Pgp3 H247A active sites bound with (**e**) BMK-S201 (orange), (**f**) BMK-S202 (olive), and (**g**) BMK-S203 (bright orange). Key interaction residues in Pgp3 are colored with light blue, whereas Zn^2+^ ion and water molecules are represented by blue and slate gray spheres, respectively. The coordination of Zn^2+^ and hydrogen bonds are represented by black dashed lines. Light blue circle with dashed line represents the Y–Y binding region.

As mentioned earlier, we hypothesized that the incorporation of a hydrophobic moiety to BMK-S101 would create additional interactions in the hydrophobic pocket in addition to Zn^2+^-hydroxamate and the Y-Y motif interactions. Based on this expectation, we synthesized three additional inhibitors (BMK-S201, BMK-S202, and BMK-S203) (Fig. 3a), and subsequently the crystal structures of Pgp3 in complex with BMK-S201 and its derivatives were obtained (Fig. 3e–g). Overall, structures of Pgp3 in complex with either BMK-S101, BMK-S201 or with the analogs were the same as that of the apo WT Pgp3. The L1 loop was in the open conformation with the bound BMK-S201, -S202, and -S203. These inhibitors bound to the deep cleft of the LytM domain with a 1:1 stoichiometry, where the pentapeptide substrate and BMK-S101 bind (Supplementary Fig. 3a). As expected, the hydroxamate group of BMK-S201 coordinated to Zn^2+^ (2.1~2.5 Å; Zn^2+^-O distance) and interacted with Nε atom of Arg242 (2.7~3.2 Å). In the Pgp3:BMK-S201 complex, a network of water molecules is present, forming interactions between Ser169 and the carbonyl group of hydroxamate (Fig. 3e).

Unexpectedly, neither the carboxylate nor the benzyl group of BMK-S201 interacted with the Y–Y motif, nor the hydrophobic pocket (Fig. 3e and Supplementary Fig. 3b). We presumed that the benzyl group in BMK-S201 might disturb the interaction between the carboxylate and the Y–Y motif. To confirm that the benzyl group is a dominant factor for the steric hindrance, we designed BMK-S202, which lacks it (Fig. 3a). In the crystal structure of Pgp3 bound to BMK-S202, the carboxylate formed hydrogen bonds with Tyr129, Tyr215, and Arg195, confirming the hypothesis. Indeed, the smaller cyclopentane ring of BMK-S203 is suitably buried within the hydrophobic pocket in Pgp3, as expected, and the Y–Y motif was engaged in the interactions with the carboxyl groups of BMK-S203 (Fig. 3b). Nonetheless, the three inhibitors (BMK-S201, -S202, and -S203) bound to Pgp3 with *K*_D_ values in the range of 1.8–6.6 μM (Fig. 3b–d), displaying essentially similar binding propensity (Fig. 2a). Overall, BMK-S203 showed a similar binding mode to BMK-S101, with the sole exception of the ensconced hydrophobic ring (Figs. 2a and 3d).

### Structural studies of BMK-S301 and its derivatives

**Fig. 4:**
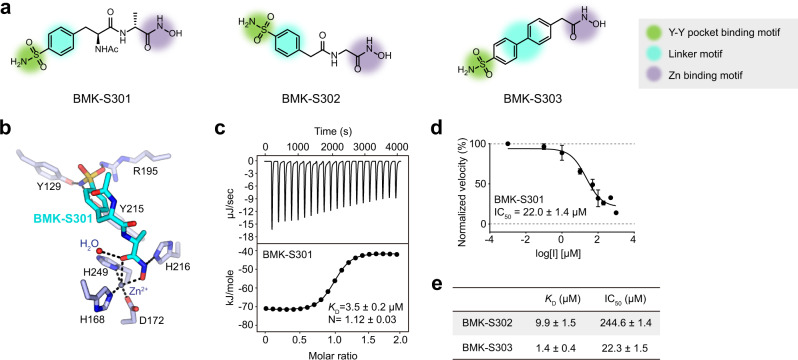
Structural and functional studies on Pgp3 in complex with BMK-S301 and its derivatives. **a** Chemical structures of BMK-S301 and its derivatives (BMK-S302 and -S303). Green, purple and cyan circles indicate the Y-Y binding motif, Zn binding motif and linker motif, respectively. **b** Detailed interaction of Pgp3 H247A active sites bound with BMK-S301 (cyan). Key interaction residues in Pgp3 are colored with light blue, whereas Zn^2+^ ion is represented by slate gray sphere. Tetrahedral coordination of Zn^2+^ and hydrogen bonds are represented by black dashed lines. **c** The ITC fitting results of Pgp3 with BMK-S301. The thermodynamic data were collected from titration of inhibitor into Pgp3, and the parameters were calculated by fitting to a single-binding model. **d** IC_50_ fitting curves for BMK-S301. The data are presented as means ± S.E.M. (*n* = 3). **e** IC_50_ and *K*_D_ value comparisons for BMK-S301 derivatives. The data are presented as means ± S.E.M. (*n* = 3).

We synthesized BMK-S301 and its analogs to reduce the flexibility of the alkyl chain on the preceding inhibitors (Fig. 4). We modified the alkyl carboxylate with the benzenesulfonamide in BMK-S301 to reduce the flexibility of the backbone, while the hydroxamate group was retained. As the sulfonamide moiety, an isostere of carboxylic acid, is known to be more permeable than a carboxylic acid and exhibits envelope permeability in bacteria^27,28^, we expected that the substitution with sulfonamide moiety might enhance compound permeability and inhibitory activity against *C. jejuni* cell. Furthermore, we designed BMK-S302 with yet a shorter backbone. To reduce the number of rotatable bonds, we conceived of BMK-S303 (Fig. 4a). The *K*_D_ values for BMK-S301, -S302, and -S303 were 3.5 ± 0.2, 9.9 ± 1.5, and 1.4 ± 0.4 μM, respectively (Figs. 4c, e and Supplementary Fig. 4a, b), indicating that the values were similar to those of the pentapeptide and BMK-S101 (Fig. 2a).

We determined the crystal structures of Pgp3 in complex with BMK-S301, BMK-S302, and BMK-S303 (Fig. 4b and Supplementary Fig. 5a, b). In the crystal structures of Pgp3 in complex with BMK-S302 and -S303, the electron densities for Zn-hydroxamate motif and Y-Y binding motif were clear, but that for connecting chain was poor. Similar to the other inhibitors, the hydroxamate in all inhibitors stably coordinated to Zn^2+^ (2.0~2.5 Å; Zn^2+^-O distance) and interacted with His168, Asp172, His216 and His249 (1.9~3.7 Å). As expected, *p*-benzenesulfonamide facilitated interactions in the Y–Y binding motif: between the sulfonamide nitrogen and Tyr129 or Try215 (2.2~2.4 or 2.3~2.8 Å, respectively) or between one of the sulfonamide oxygens and Arg195 (2.4~2.8 Å). Also, the IC_50_ values of each inhibitor were determined based on the reduction of enzyme activity. BMK-S301 and -S303 showed micromolar IC_50_ values (22.0 ± 1.4 µM and 22.3 ± 1.5 µM, respectively) (Fig. 4d, e and Supplementary Fig. 4d).

### Morphology change of *C. jejuni* on exposure to the inhibitors

**Fig. 5:**
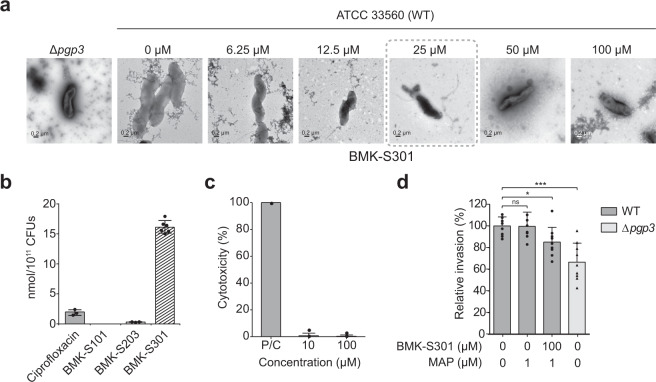
Cell morphology change, cell penetration, and invasion assays in cultured *C. jejuni* cells. **a** TEM analysis of *C. jejuni* strains upon BMK-S301 treatment. *C. jejuni* ATCC 33560 WT was negatively stained with 2% (w/v) uranyl acetate and then observed by using TEM. For inhibitor-treated sample, each concentration of inhibitor was incubated in 37 °C for 24 h before staining. Scale bars (lower left) represent 0.2 μm. The minimum concentration of inhibitors which shows changes in the cell morphology is highlighted in gray dashed box. **b** Accumulation of Pgp3 inhibitors in *C. jejuni* cells. The BMK-S203 and S301 that are active against *C. jejuni* than BMK-S101 exhibit accumulation in the bacteria. BMK-S301 is the most permeable compound through the outer membrane of *C. jejuni*. Ciprofloxacin was used as a reference compound for the accumulation assay (P/C, positive control). **c** The cytotoxicity of BMK-S301 for Caco-2 epithelial cells. Cytotoxicity was determined using LDH activities released from Caco-2 epithelial cells treated with the 10 or 100 μM BMK-S301 for 3 h. Cells were lysed with 2% Triton X-100 for 15 min to release all LDH to 100%. **d** The effect of BMK-S301 on the host cell invasion. 100 μM BMK-S301 with 1 μM MAP (membrane-active peptide, KL-L9P) was used for invasion assay. Caco-2 epithelial cells were with *C. jejuni* ATCC 33560 WT and the numbers of intracellular bacteria were determined 3 h after infection using the gentamicin-protection assay. Error bars represent the means and SEM from three independent experiments. The asterisk shows statistical significance (*p < 0.05; ***p < 0.001; ns not significant). The source data is provided in Supplementary Data 2.

Next, we investigated whether our inhibitors could transform *C. jejuni* from the helical- to curved-rod morphology on exposure to the inhibitors (Fig. 5). We had previously constructed a *C. jejuni pgp3*-deletion mutant and examined it by transmission-electron microscopy (TEM), displaying a curved-rod morphology^26^. To investigate the effect of inhibitors on the morphology of *C. jejuni* cells, we exposed the cells to the inhibitors and checked whether the morphology could be altered. For improved permeability of inhibitors to *C. jejuni*, we treated a cationic kinked alpha-helical membrane-active peptide (KL-L9P), which selectively enhances permeation of antibiotics through the Gram-negative membrane without damaging eukaryotic membrane, when co-treated with antibiotics^29^. Indeed, we observed a concentration-dependent morphology change from spiral to curved-rod shape upon treatment with BMK-S101 and BMK-S301 (Fig. 5a and Supplementary Fig. 6a). We could not detect any changes in the cell shape at lower concentrations of BMK-S101 (≤156 μM). However, at higher concentrations (>313 μM), *C. jejuni* displayed an “unwound” straight morphology (Supplementary Fig. 6a). Similarly, treatment with BMK-S301 at a concentration of 25 μM or higher resulted in morphological changes of *C. jejuni* (Fig. 5a). The minimum concentrations of BMK-S202 and -S203 causing morphological changes were 25~50 μM, whereas 100 and 400 μM for BMK-S302 and -S303, respectively, were necessary for the same effect (Supplementary Fig. 6b).

### Cell penetration of the inhibitors and invasion of *C. jejuni*

Although all inhibitors exhibited *K*_D_ values within a limited range (mid nanomolar to low micromolar), the concentrations manifesting the morphological change were substantially different among them (BMK-S101, >313 μM and BMK-S301, 25 μM). We performed cell-penetration assay to explore whether penetration through the outer membrane of *C. jejuni* cells played a role in the efficacy of the inhibitors (Fig. 5b). After treating the *C. jejuni* cells with the inhibitors, we quantified their penetration by mass spectrometry. We obtained the intracellular concentrations of ciprofloxacin (a positive control) and that of BMK-S203 at 2.0 ± 0.5 nmole/10^11^ CFU and 0.3 ± 0.1 nmole/10^11^ CFU, respectively. BMK-S301 exhibited the highest accumulation at 16.2 ± 1.0 nmole/10^11^ CFU, as much as 8- and 54-folds higher than for ciprofloxacin and BMK-S203, respectively (Fig. 5b). We conclude that the more potent response to BMK-S301 is due to the superior penetration into *C. jejuni*.

We next investigated the effect of inhibitors in an invasion assay using Caco-2 cells, as a measure of virulence. Previously, we showed that the *pgp3*-deletion mutant of *C. jejuni* exhibited decreased invasive ability compared to the WT, while the invasive ability was restored appreciably after complementation^26^. Although BMK-S301 was not toxic to the Caco-2 cells (Fig. 5c), the invasion ability of the WT *C. jejuni* treated with 100 μM BMK-S301 and 1 μM KL-L9P, which is a membrane-active peptide, was decreased compared to that of the untreated *C. jejuni* in a high-viscosity growth medium (Fig. 5d). The invasion ability of WT *C. jejuni* treated with 100 μM BMK-S301 was also comparable to that of *pgp3*-deletion mutant. These results indicate that BMK-S301 induces the morphological change of *C. jejuni* cells from helical to curved-rod shape on inhibition of Pgp3 and that the consequence is diminished virulence of *C. jejuni* at statistically significant levels.

### Kinetic studies of Pgp3

Prior to investigating the inhibition mode of BMK-S101, we measured the enzymatic activity of Pgp3 by increasing the concentrations of a pentapeptide-derived substrate analog. This FRET substrate was prepared by the reaction of fluorescein isothiocyanate (FITC) linked to l-Ala^1^ of the pentapeptide, and 4′-([4′-(dimethylamino)phenyl]­azo)­benzoic acid (DABCYL) placed after d-Ala^5^, which is substituted with lysine in this substrate (Supplementary Fig. 7). Because DABCYL/FITC is a typical FRET quencher–fluorophore pair, an increase in fluorescent signals can be directly detected when it is processed by Pgp3 (Tang et al., 2013). The kinetic parameters (*k*_cat_ = 0.0209 ± 0.0006 s^−1^ and *K*_M_ = 6.6 ± 0.7 µM) for the substrate were obtained by fitting the data to the Michaelis-Menten equation (Supplementary Fig. 8a).

### The inhibition mechanism

All seven X-ray structures of Pgp3 showed that the inhibitors bound to the active site, consistent with the competitive mechanism of inhibition by the kinetic analysis (Supplementary Fig. 8b). In the apo H247A variant, two water molecules (Wat-1 and Wat-2) occupy the active site, of which Wat-1 is the hydrolytic water for the turnover process. Wat-2 exists in the space that the substrate would occupy. The scissile carbonyl oxygen of the substrate displaces Wat-2, thereby interacting directly with Zn^2+^, rendering the amide bond susceptible to nucleophilic attack by Wat-1^26^. In the case of our inhibitors, Wat-2 is displaced with the oxygen atom of the hydroxamate moiety. It is notable that Wat-1 is displaced by the other hydroxamate oxygen atom in the inhibitor-bound structure. The oxygen atom of hydroxamate replaces the Wat-1-mediated hydrogen-bond network between the Zn^2+^ ion and a nitrogen of Arg242 (Fig. 2c). Taken together, we document that the two hydroxamate oxygen atoms replace the key active-site Wat-1 and Wat-2, and the bulk of the inhibitor as it is sequestered in the active site, would block the approach of the substrate.

### Molecular-dynamics simulations of Pgp3 with inhibitors

We further investigated the dynamical interactions of the BMK-S101 (7E60), -S203 (7E63), and -S301 (7E65) using computational simulations. The X-ray structures were prepared, immersed in a box of water molecules, energy-minimized, and subjected to molecular-dynamics simulations for 700 ns each with PMEMD module of AMBER18^30^, following previous protocols^26,31^ (see methods for details).

The BMK-S101 complex (7E60) demonstrated considerable motion in the deep channel. The hydroxamate group coordinated with the catalytic Zn^2+^ and served as an anchor. The carboxylate group of the terminal *m*DAP, which formed an electrostatic interaction with Arg195, adjusted its position early in the simulation. During the simulation, the *m*DAP lost interaction with Arg195 and flipped out of the pocket (Supplementary Movie 1). Further along the simulation, the *m*DAP returned to the initial pocket, where it remained for the rest of the simulation. The dynamical nature of the ligand in the pocket correlated with the dispersed electron density of the ligand in the crystal structure (Supplementary Fig. 2). Compared to the complex for BMK-S101, that for BMK-S203 (7E63) demonstrated better stability in the pocket (Supplementary Movie 2). Although the cyclopentyl ring did not appear to fit deep in the hydrophobic pocket (lined by Leu122, Ile118, and Phe197; Supplementary Fig. 3b), it demonstrated favorable hydrophobic interaction with the side chain of Phe197. The cyclopentyl group appeared mobile, which is consistent with the weaker electron density for it in the crystal structure.

The BMK-S301 (7E65), the most potent among the three inhibitors, demonstrated the most stable interactions during the simulation (Supplementary Movie 3). The phenyl sulfonamide group occupied deep in the pocket. The sulfonamide group demonstrated rearrangement in the pocket and formed hydrogen bonds and long-range electrostatic interactions with Arg195. The shape of the channel is contributed by Phe197 and the stable interactions of the ligand with this residue likely improves the binding. In the case of BMK-S301, the methyl group of the acetamide and the phenyl group appear to closely interact with Phe197. In addition, the position and orientation of d-Ala within the inhibitor, found also in the substrate, seems to favor the overall binding of the ligand. In summary, the simulations explained the dynamical nature of the active site and the binding features of the ligand.

## Discussion

Pgp3 is a zinc-dependent metallopeptidase, with selective catalytic ability for hydrolysis of the cross-linked muramyl tetra-tripeptide or the pentapeptide in the peptidoglycan strands in *C. jejuni*. These reactions are linked to the invasive ability of *C. jejuni* into the host epithelial cells. We set out to explore whether targeting cell morphological determinants could be a new anti-virulence strategy. We have described herein the structure-based design of Pgp3 inhibitors and a proof-of-concept for the hypothesis.

Previously, we determined the crystal structure of pentapeptide-bound Pgp3 H247A variant, enabling us to capture a snapshot of catalytic states of Pgp3 with its substrates^26^. The knowledge of the crystal structure of Pgp3 in complex with its substrates provided an opportunity for designing of inhibitors for this enzyme as a potential pharmacological intervention in *C. jejuni* infections. Based on the structure, we accessed three key motifs (zinc-binding motif, the hydrophobic pocket, and the Y–Y binding motif) and synthesized seven inhibitor candidates in an iterative manner, guided by X-ray structures at every step. Structural studies on Pgp3:inhibitor complexes and various biochemical assays showed that these inhibitors can competitively inhibit Pgp3. Notably, the strategy to strengthen the rigidity of the inhibitor and applying sulfonamide as an outer-membrane-permeability enhancer identified the most potent inhibitor candidate (BMK-S301). Inhibitor BMK-S301 showed better inhibitory activity in the IC_50_ assay and decreased the invasive ability of *C. jejuni* at statistically significant levels. However, further studies are necessary to identify candidates for Investigation New Drug-enabling studies.

Recently, small-molecule inhibitors targeting cell-shape-determining proteins, such as MreB, Csd4 and Pgp1 have been studied^32–35^. These strategies are based on screening of small compounds or peptide analogs^3^. In contrast, we investigated a structure-based inhibitor-design strategy based on the X-ray structure of Pgp3 in complex with its substrates. The structure-based strategy led to three generations of inhibitors, in an iterative manner. We note that Pgp3 inhibitors bind to the active site in the LytM domain, which is highly conserved in various helical bacteria^36^. Thus, our strategies to inhibit the catalytic activity of the LytM domain might have broad applicability to other pathogenic spirilla such as *Helicobacter pylori* or spirochetes.

Collectively, our inhibitors block the d,d-endopeptidase and d,d-carboxypeptidase activities of Pgp3, which are essential for the cellular shape of *C. jejuni*, and its virulence. These results demonstrate that the change of *C. jejuni* morphology has an effect on its virulence, and provide a blueprint for further work.

## Methods

### Chemical synthesis information

Reactions that needed anhydrous conditions were carried out in flame-dried glassware under positive pressure of dry N_2_ using standard Schlenk-line techniques. TLC was performed using silica gel 60F254 coated on aluminum sheet (E. Merck, Art.5554). Column chromatography was performed on silica gel (Merck. 7734 or 9385 Kiesel gel 60), and the eluents are mentioned in each procedure. The ^1^H NMR-spectra and ^13^C NMR-spectra were measured on a Varian/Oxford As-500 (500 MHz) spectrophotometer. Chemical shifts were measured as parts per million (δ values) from tetramethylsilane as an internal standard at probe temperature in chloroform-*d*, methanol-*d*_4_, deuterium oxide, dimethyl sulfoxide-*d*_6_ or acetonitrile-*d*_3_. Coupling constants are provided in Hz, with the following spectral pattern designations: s, singlet; d, doublet; t, triplet; q, quartet; quint, quintet; m, multiplet; br, broad; app, apparent. High-resolution mass spectra (HRMS) were recorded on a ThermoFinnigan LCQ™ Classic, Quadrupole Ion-Trap Mass Spectrometer. HPLC analyses were carried out on an Agilent HP1100 system (Santa Clara, CA, USA), comprised of an auto sampler, quaternary pump, photodiode array detector (DAD), and HP Chemstation software. The separation was carried out on a poroshell 120 EC-C18 column 4.6 × 50 mm i.d. (2.7 µm particle size) with 0.1% TFA in water (A) and acetonitrile (B) as a mobile phase at a flow rate of 1 mL/min at 20 °C. Method: 100% A and 0% B (0 min), 50% A and 50% B (5 min), 5% A and 95% B (15 min), 5% A and 95% B (22 min), 100% A and 0% B (23 min), 100% A and 0% B (25 min). Preparative HPLC was carried out on an Agilent 1260 Infinity system (Santa Clara, CA, USA; solvent system: acetonitrile:water with 0.1% TFA; 0–5 min, 5% acetonitrile; 5–12 min, 5-45% acetonitrile; 12–15 min, 45–95% acetonitrile; 15–18 min, 95% acetonitrile; 18-19 min, 95–5% acetonitrile; 19–20 min, 5% acetonitrile; flow rate: 10.0 mL/min; column: Zorbax C18 column, 5 μm, 80 Å, 21.2 × 150 mm). Details are given in Supplementary Methods for one example of each reaction type. All materials were obtained from commercial suppliers and used without further purification, unless otherwise noted.

#### Protein expression and purification

The gene encoding the amino-acid sequence of Glu21–Gln273 of Pgp3 from *C. jejuni* (strain ATCC 33560) was cloned into the expression vector pET-21a (Novagen) producing the recombinant wild-type Pgp3 protein with the C-terminal His_6_-tag (LEHHHHHH). The protein expression and purification were conducted according to previously described methods with exception^26^. The HiTrap chelating HP (GE Healthcare) column was washed with the lysis buffer containing 60 mM imidazole and the protein was eluted with a linear gradient from 100 to 500 mM imidazole. The protein was further purified by gel filtration on a HiLoad 16/60 Superdex 200 prep-grade column (GE Healthcare), which was previously equilibrated with 20 mM Tris-HCl pH 8.0 and 200 mM NaCl. The fractions containing the Pgp3 protein were pooled and concentrated to 15 mg mL^−1^ for crystallization. The variants of Pgp3 (S169A, R195A, H216A, and H247A) were generated by site-directed mutagenesis using primer sets in Supplementary Table 1 and were purified as described for the WT Pgp3.

#### Crystallization and data collection

We determined seven structures of Pgp3 H247A variant in complex with BMK-S101, -S201, -S202, -S203, -S301, -S302, and -S303. Before crystallization, H247A Pgp3 (15 mg mL^−1^) and inhibitors (70 mg mL^−1^) in 20 mM Tris-HCl pH 8.0 and 200 mM NaCl were mixed in a 1:10 molar ratio and incubated at 4 °C for 1 h. Final protein concentration was 10 mg mL^−1^. Crystals of H247A Pgp3 in complex with BMK-S101 were grown at 296 K by the sitting-drop vapor-diffusion method using the Mosquito robotic system (TTP Labtech). The sitting drop was prepared by mixing equal volumes of the protein:inhibitor solution (0.2 μL) and reservoir solution (0.2 μL) containing 0.1 M HEPES pH 7.5 and 1.4 M sodium citrate tribasic dihydrate. Crystals of the H247A Pgp3:BMK-S101 complex were cryoprotected in the reservoir solution supplemented with 10% (*v/v*) glycerol and were flash-frozen in a nitrogen gas stream at 100 K. Data for the complex of BMK-S101 with H247A Pgp3 were collected at 2.2 Å resolution, using the EIIGER4M detector at the beamline BL26B1 of Spring-8, Japan.

For the BMK-S201-bound form, the sitting drop was prepared by mixing equal volumes of the protein:inhibitor solution (0.2 μL) and the reservoir solution (0.2 μL) containing 0.1 M sodium acetate: acetic acid pH 4.5 and 0.8 M NaH_2_PO_4_/1.2 M K_2_HPO_4_. Crystals of the H247A Pgp3:BMK-S201 complex were cryoprotected in the reservoir solution supplemented with 20% (*v/v*) glycerol and were flash-frozen in a nitrogen gas stream at 100 K. Native data for the BMK-S201-bound H247A Pgp3 were collected at 1.8 Å resolution, using the EIIGER9M detector at the beamline 5 C of Pohang Light Source (PLS).

For complexes with BMK-S202 and -S302, crystals of the apo H247A Pgp3 were grown at 296 K by the sitting-drop method in the reservoir solution containing 0.2 M potassium sodium tartrate tetrahydrate, 0.1 M sodium citrate tribasic dihydrate pH 5.6 and 2 M ammonium sulfate. Crystals were soaked for 1–20 min and cryoprotected in the reservoir solution supplemented with 20 mM of each inhibitor and 30% (*v/v*) glycerol. Soaked crystals were flash-frozen in a nitrogen gas stream at 100 K. For the complex with BMK-S303, the apo crystals of H247A Pgp3 were grown in the reservoir solution containing 0.2 M ammonium sulfate, 0.1 M sodium acetate trihydrate pH 4.6, and 30% polyethylene glycol monomethyl ether 2000. The complexes with BMK-S203 and -S301, the reservoir solution was 0.1 M sodium citrate tribasic dihydrate pH 5.5 and 22% PEG 1000 for the apo crystals of H247A Pgp3. Each crystal was soaked for 1–20 min and cryoprotected in the reservoir solution supplemented with 20 mM of each inhibitor and 20% (*v/v*) glycerol. All native data were collected at 1.92~2.85 Å resolutions using the EIIGER9M detector at the beamline 5 C of PLS. X-ray diffraction data for all crystals were processed and scaled using the program suit HKL2000^37^ and XDS. Data collection statistics are summarized in Supplementary Table 2.

#### Structure determination and refinement

Seven structures of Pgp3 H247A variant in complex with inhibitors were determined by molecular replacement utilizing the program MOLREP, with H247A Pgp3 apo structure (PDB code 6JMZ) as a reference model^26^. Manual model building was performed using the program COOT, and models were refined with the program REFMAC5, including the bulk-solvent correction^38,39^. Five percentage of the total data were randomly set aside as test data for calculating *R*_free_^40^. Further manual model building and restrained refinement were carried out using the COOT program the REFMAC5 program, respectively^39^. Several rounds of model building - simulated annealing - positional refinement - individual *B*-factor refinement were performed to resolve the structures. The stereochemistry of the refined models was assessed using the MolProbity interface^41^. Crystallographic and refinement statistics are summarized in Supplementary Table 2. In the case of the complexes with BMK-S203 and BMK-301, inhibitors were modeled into an electron-density map in only one subunit per asymmetric unit due to weak electron density. Atomic coordinates and structure factors of seven crystal structures of Pgp3 in complex with inhibitors have been deposited in PDB (PDB ID codes 7E60, 7E61, 7E63, 7E64, 7E65, 7E66, and 7E67).

#### Isothermal-titration calorimetry

ITC experiments were performed using Affinity ITC instruments (TA Instruments, New Castle, DE, USA) at 298 K. For *K*_D_ values of the pentapeptide and all inhibitors, 150 μM of Pgp3 WT or H247A (for pentapeptide) prepared in a buffer containing 20 mM Tris-HCl pH 8.0 and 200 mM NaCl were degassed at 295 K prior to measurements. Using a micro-syringe, 2.5 μL of each inhibitor (2.25 mM) was added at intervals of 200 s to the Pgp3 solution in the cell with gentle stirring (125 rpm). The same ITC condition was applied to evaluate the *K*_D_ values of BMK-S101 for each Pgp3 variant. All data were fitted to the single-binding-site model. Fitted curves were generated using GraphPad Prism 7. Data are mean ± SEM from triplicate experiments and the ITC thermograms are representative of triplicate experiments.

#### Enzyme kinetics and inhibition

The enzymatic assay for Pgp3 activity was determined using a fluorescein isothiocyanate (FITC) and 4′-([4′-(Dimethylamino)phenyl]­azo)­benzoic acid (DABCYL) pair for FRET. The FITC fluorophore was linked at l-Ala^1^ of pentapeptide, whereas DABCYL was linked at the position of d-Ala^5^ in pentapeptide (Supplementary Fig. 7). Detailed synthesis of the modified pentapeptide (FITC-L-Ala^1^-D-Glu^2^-mDAP^3^-D-Ala^4^-L-Lys^5^-DABCYL) is described in chemical synthesis information. Using the modified pentapeptide as a substrate, fluorescence intensities were measured by a microplate reader (Synergy H1, BioTek, USA). The excitation and emission spectra were set at 495 nm and 525 nm, respectively. Pgp3 (0.1 μM) in 20 mM HEPES pH 7.5, 200 mM NaCl, and 0.4 μM of zinc was used in this assay. The reaction was initiated by adding 3 μL of 0.015–2 mM substrate (final 1.56–200 μM) in the total volume of 30 μL of the reaction mixture. Initial velocities were calculated using slopes of fluorescence signal intensity in 3 min after the initiation of the reaction. In order to elucidate the inhibition mechanism of BMK-S301, initial velocities with various concentrations of substrates were measured in the presence of 40 μM BMK-S301. To determine the *k*_cat_ and *K*_M_ values, the fluorescence of a pentapeptide-derived substrate without DABCYL quencher, which is a reaction product of Pgp3 cleavage, was used for standard curve. The same method was used for the determination of the IC_50_ values for inhibitors. Inhibitors in each concentration were pre-incubated with 0.1 μM of Pgp3 for 1 h on ice, and initial velocities were determined for 0.1, 10, 50, 100, 200, 500, and 1000 μM of each inhibitor in reaction with 10 μM of substrate. The IC_50_ values were calculated using GraphPad Prism 7.

#### Bacterial strains and culture conditions

The wild-type *C. jejuni* ATCC 33560 and the *pgp3*-deletion mutant strain used in this work were reported previously (Min et al., 2020). These strains were grown at 37 °C or 42 °C in Mueller-Hinton (MH) media (Oxoid, UK) under a micro-aerobic condition (5% O_2_, 10% CO_2_, 85% N_2_). Occasionally, MH media were supplemented with kanamycin (50 µg/mL).

#### Cell morphology change monitored by using transmission-electron microscopy (TEM)

Cell-morphology change of *C. jejuni* upon inhibitor treatment was monitored, as described previously with modifications^35^. Briefly, overnight cultures of *C. jejuni* were harvested from MH agar plates and suspended in MH broth to approximately 1 × 10^6^ colony-forming unit (CFU)/mL. A total of 500 μL of the diluted bacterial culture was added into each well of a 24-well plate with 5 μL of two-fold serial dilutions of inhibitor. For the control strain with no inhibitor, the same volume of dilution buffer (20 mM Tris-HCl pH 8.0 and 200 mM NaCl) was used. The plate was incubated under a micro-aerobic condition at 37 °C for 24 h in the static condition. After incubation, 10 μL of the bacterial cultures was placed on formvar/carbon-coated copper grids (200 mesh) and negatively stained with 2% uranyl acetate for 2 s. The morphology of bacteria was observed with an energy-filtering transmission microscope (EF-TEM; Libra 120, Germany) at voltage of 120 kV.

#### Drug accumulation assay

The accumulation assay was adapted from two different methods^42,43^. The *C. jejuni* (ATCC 33560) colonies on a Müller-Hinton II (MHII) agar plate were resuspended in 40 mL of MHII broth to be optical density at 600 nm (OD_600_) of 0.002 (approximately 10^7^ CFU/mL). The culture was grown overnight at 42 °C under the micro-aerobic condition. The cells were harvested at 6000 *g* for 15 min and washed with 1 × PBS, followed by resuspension in pre-warmed 1 × PBS to ~10^10^ CFU/mL. The cell suspension (1.2 mL) was dispensed into a 24-well plate. Each compound to be examined was added into each well (final concentration = 100 μM). Ciprofloxacin was used as a positive control, since its accumulation in *C. jejuni* has been proved^43^. After incubation with shaking at 37 °C for 20 min, OD_600_ of each sample was measured, and the cells were harvested at 6000 *g* for 10 min at 4 °C. The pellets were resuspended in 250 μL of water, followed by three cycles of Freeze-Thaw with a dry ice/ethanol bath and a 37 °C-heating block in order to extract the compounds from *C. jejuni*. The cells were pelleted at 13,000 *g* for 5 min at room temperature. The supernatants (220 μL) were transferred to new tubes. The pellets were resuspended in 100 μL of methanol with vigorous vortexing for 1 min, followed by pelleting as before. The supernatants (100 μL) were combined with the solutions previously collected. The residual cell debris were removed at 20,000 *g* for 15 min at room temperature. The supernatants (300 μL) were dried with Genevac™ miVac Centrifugal Concentrator at room temperature for 5 h. Finally, the samples were resuspended with appropriate solvents and analyzed by LC-MS.

#### Liquid chromatography/mass spectrometry

The concentrated sample from accumulation assay was reconstituted in 98:2 of water:acetonitrile (ACN) for BMK-S101, -S203, and -S301, and in 95:5 of water:ACN for ciprofloxacin. The suspension was centrifuged and the supernatant was analyzed by LC-MS. LC-MS systems consists of a Bruker impact II ultra-high resolution Qq-time-of-flight mass spectrometer using Hystar 5.0 SR1 software with a Waters Acquity UPLC H-Class system equipped with a quaternary solvent manager, a sample manager-FTN, and a photodiode array detector. The Bruker electrospray ionization source was operated in the positive ion mode, except for BMK-S301 (in the negative ion mode). The chromatographic separation was accomplished either on a Waters Acquity UPLC HSS T3 column (2.1 × 150 mm, 1.8 μm) for BMK-S101, -S203, and -S301 or a Waters Acquity UPLC BEH C18 column (2.1 × 50 mm, 1.7 μm) for ciprofloxacin. A four-step 10-min LC gradient was as follows: 3 min at 98:2; 3 min to 85:15; 0.1 min to 98:2; 3.9 min at 98:2 for BMK-S101, -S203, and -S301. The ratio of the mobile phases (A = 0.1% formic acid in water, B = 0.1% formic acid in ACN) is given as A:B in LC gradients. For ciprofloxacin, the following LC gradient was used: 4 min from 95:5 to 85:15; 3.9 min to 20:80; 0.1 min to 95:5; 2 min at 95:5. The quantification was done by integrating the peak areas from extracted-ion chromatograms of corresponding *m*/*z* values of each compound. The quantity of the compounds was normalized as nmole/10^11^ CFU.

#### Cytotoxicity assay

To examine the cytotoxicity of BMK-S301, the lactate dehydrogenase (LDH) activity was measured using Cytotoxicity Detection Kit^PLUS^ (LDH; Roche, Switzerland) according to the manufacturer’s instructions. Briefly, the monolayers of Caco-2 cells were prepared in a 96 well tissue culture plate and incubated in Dulbecco’s modified-eagle medium (DMEM; ATCC) with 10% fetal bovine serum (FBS; Invitrogen), and 0.6% carboxymethylcellulose (CMC) at 37 °C under 5% CO_2_. The BMK-S301 (10 or 100 μM) was treated, and plate was incubated for 3 h. The 2% Triton X-100 was used to release all LDH of Caco-2 cell as a positive control.

#### Invasion assay

Invasion assay was performed as described previously with modifications^44^. Briefly, prior to bacterial infection, a monolayer of 1 × 10^5^ Caco-2 cells was prepared in a 24-well tissue culture plate and incubated in DMEM with 10% FBS, and 0.6% CMC at 37 °C under 5% CO_2_. The cultures of wild-type *C. jejuni* strain with or without BMK-S301 (100 μM) and KL-L9P (1 μM) were prepared, as described above (TEM). The bacterial suspensions were diluted in DMEM with 10% FBS and 0.6% CMC, and then added onto the cell monolayer at a multiplicity of infection (MOI) of 100. After a 3 h incubation, the cells were incubated with 250 μg/mL of gentamicin in the fresh pre-warmed medium for 2 h to kill any extracellular bacteria. The wells were washed three times with 1 × PBS, and treated with 0.1% Triton X-100 in 1 × PBS for 15 min. After dilution with 1 × PBS, the suspensions were plated on MH agar to enumerate the CFU. All experiments were done in triplicate.

#### Statistics and reproducibility

Statistical analysis was performed using the GraphPad Prism 5.01 software. All results were analyzed by Student’s unpaired *t*test. The data are presented as means and standard errors of the mean (SEM).

#### Molecular dynamics simulation

The X-ray structures of the protein-ligand complexes were prepared with the Maestro program (Schrodinger Suite 2020-1), during which hydrogen atoms were added and protonation states of residues were assigned. Forcefield parameters for protein and ligands were obtained from ff19SB and GAFF respectively. Metal Center Parameter Builder (MCPB)^45^ module implemented in AmberTools20^30^ package was used to build the parameters of active-site Zn^2+^ ion, coordinated ligands, and protein residues (His168, Asp172, His249), following the bonded-model approach^26,45^. Charges for the atoms in the metal center and ligands were calculated with Gaussian16 program^46^ at B3LYP/6-31 G* level of theory and incorporated with RESP methodology^47^. Leap module of AmberTools20 was used to immerse the complex, centered in a rectangular box of water molecules (TIP3P model) with edges at least 10 Å away from the protein surface. The whole system was neutralized by addition of chloride ions. Molecular-dynamics simulations of the protein-ligand complexes were performed with GPU-accelerated PMEMD module^48^ of AMBER18^30^. The systems were energy-minimized and equilibrated for 500 ps in stages, following a previously reported protocol^31^. The equilibrated system was subjected to the production molecular-dynamic simulation in an isothermal-isobaric (NPT) ensemble for 700 ns. Langevin thermostat^49^ (collision frequency of 2.0 ps^−1^) and Berendsen barostat^50^ were used to maintain constant temperature (300 K) and pressure (1 atm), respectively. All bonds involving hydrogen atoms were constrained with SHAKE algorithm^51^ to allow a time step of 2 fs for the simulations. Periodic boundary condition was applied and a cutoff value of 8 Å was set for non-bonded interactions. Longer-range electrostatics were treated with Particle Mesh Ewald method^52^. Trajectories from the simulations were post-processed with CPPTRAJ^53^ module of AmberTools20 and analyzed with VMD program^54^. The AMBER topology and coordinate files are uploaded in Supplementary Data 1.

### Reporting summary

Further information on research design is available in the Nature Research Reporting Summary linked to this article.

## Supplementary information

Supplementary Information

Description of Additional Supplementary Files

Supplementary Data 1

Supplementary Data 2

Supplementary Movie 1

Supplementary Movie 2

Supplementary Movie 3

Reporting Summary

## Data Availability

Crystallographic coordinates of the Pgp3:BMK-S101, -S201, -S202, -S203, -S301, -S302, and -S303 complexes have been deposited in the RCSB Protein Data Bank, www.wwpdb.org, with accession number 7E60, 7E61, 7E63, 7E64, 7E65, 7E66, and 7E67, respectively. Source data files are provided in Supplementary Data 1 and 2.
