# Supplementary material for: Structure-based inhibitor design for reshaping bacterial morphology
Source: Commun Biol. 2022 Apr 28;5:395. doi: 10.1038/s42003-022-03355-3 (PMC9050674; doi:10.1038/s42003-022-03355-3)
Supplement: Supplementary file 1 — Supplementary Information [file 42003_2022_3355_MOESM1_ESM.pdf]

Supplementary Information

Structure-based inhibitor design for reshaping bacterial morphology

Yuri Choi[1,‡], Ji Su Park[1,‡], Jinshil Kim[2,3,‡], Kyungjin Min[1,‡], Kiran Mahasenan[4], Choon Kim[4], Hye-Jin Yoon[1], Sewon Lim[1], Dae Hee Cheon[1], Yan Lee[1], Sangryeol Ryu[2,3], Shahriar Mobashery[4*], B. Moon Kim[1*], and Hyung Ho Lee[1*]

[1]Department of Chemistry, College of Natural Sciences, Seoul National University, Seoul 08826, Korea

[2]Department of Food and Animal Biotechnology, Department of Agricultural Biotechnology, and Research Institute for Agriculture and Life Sciences, Seoul National University, Seoul 08826, Korea

[3]Center for Food and Bioconvergence, Seoul National University, Seoul 08826, Korea

[4]Department of Chemistry and Biochemistry, University of Notre Dame, Notre Dame, Indiana, 46556, USA

[‡]These authors contributed equally to this work.

[*]E-mail: mobashery@nd.edu; kimbm@snu.ac.kr; hyungholee@snu.ac.kr

Key words: Inhibitor, peptidoglycan, bacterial cell wall, antibiotics, crystal structure, cell shape

This PDF file includes:

Supplementary Methods

Supplementary Figures 1 to 8

Supplementary Tables 1 to 2

Caption for Supplementary Movies 1 to 3

Caption for Supplementary Data 1 to 2

Supplementary Reference

List of Abbreviations

AC_2O	Acetic anhydride
Ahx	Aminohexanoic acid
Ala	Alanine
Boc	*tert*-butoxycarbonyl
Dabcyl	4-(dimethylaminoazo)benzene-4-carboxylic acid
DCE	1,2-Dichloroethane
DCM	Dichloromethane
DIC	N,N'-Diisopropylcarbodiimide
DIPEA	*N,N*-diisopropylethylamine
DMAP	4-Dimethylaminopyridine
DMF	*N,N*-dimethylformamide
EDCI	1-(3-Dimethylaminopropyl)-3-ethylcarbodiimide Hydrochloride
FITC	Fluorescein isothiocyanate
Fmoc	Fluorenylmethylcarbonyl
FRET	Förster Resonance Energy Transfer
Glu	Glutamic acid
HCl	Hydrogen chloride
H_2O	Water
HBTU	Hexafluorophosphate Benzotriazole Tetramethyl Uronium
HOBt	1-Hydroxybenzotriazole
HPLC	High-performance liquid chromatography
K_2CO_3	Potassium carbonate
LC-MS	Liquid chromatography-mass spectrometry
Lys	Lysine
MeOH	Methanol
$MgSO_4$	Magnesium sulfate
NaCl	Sodium chloride
NaOH	Sodium hydroxide
NMR	Nuclear magnetic resonance
PtO_2	Platinum (IV) oxide
RP-HPLC	Reverse phase-high performance liquid chromatography
RT	Room temperature
TFA	2,2,2-trifluoroacetic acid
THF	Tetrahydrofuran
TEA	Triethylamine
TES	Triethylsilane
TIPS	Triisopropylsilane
TLC	Thin-layer chromatography

Supplementary Methods

Synthetic schemes

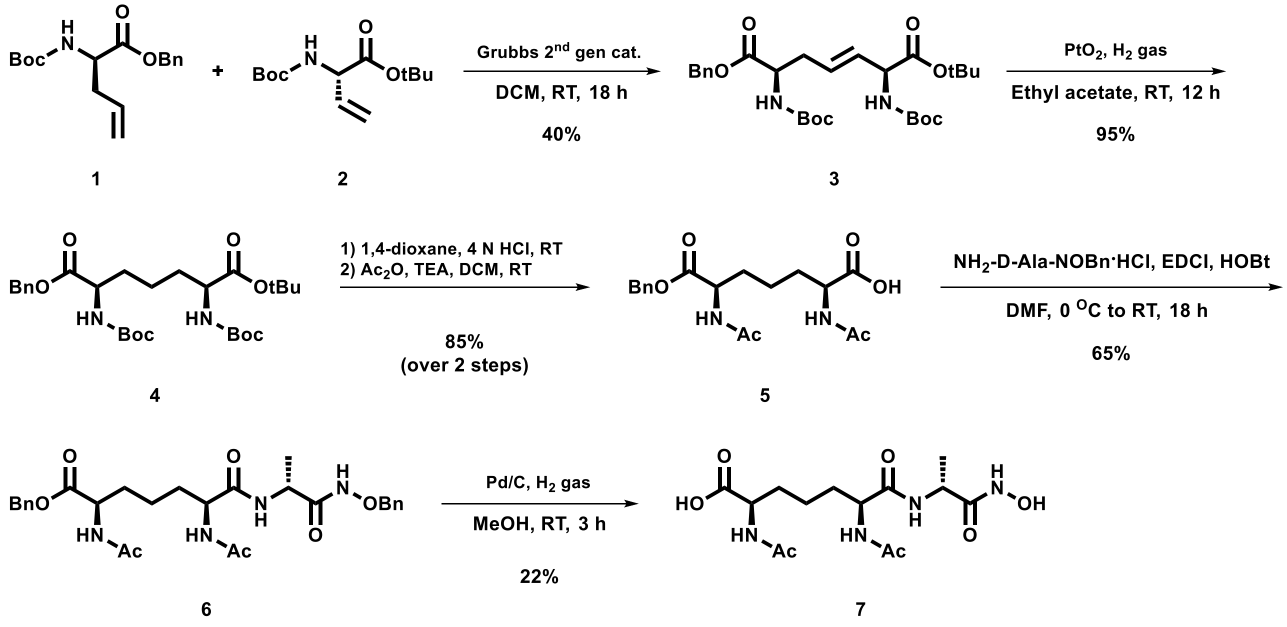

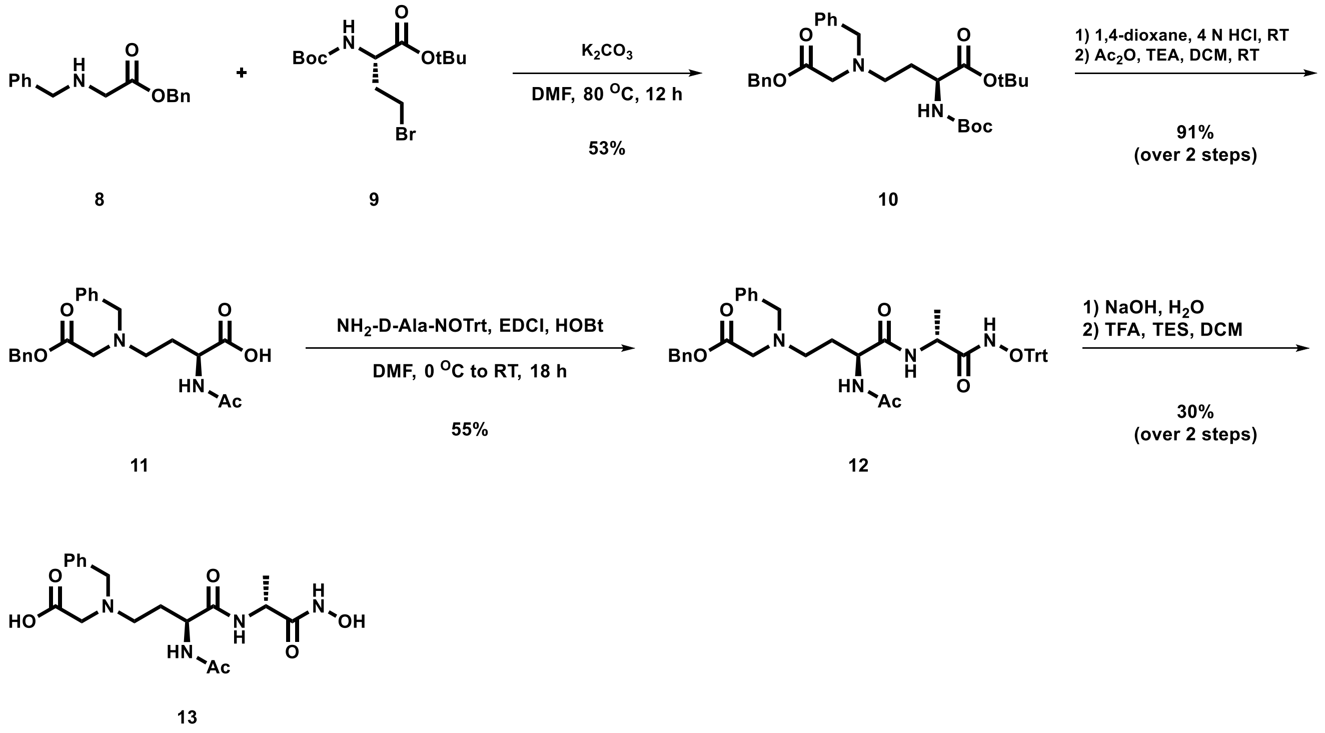

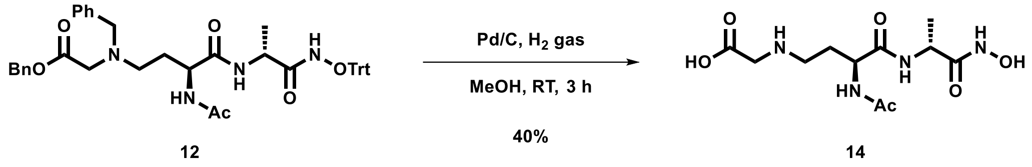
Pd/C, H2 gas
MeOH, RT, 3 h
40%
12
14

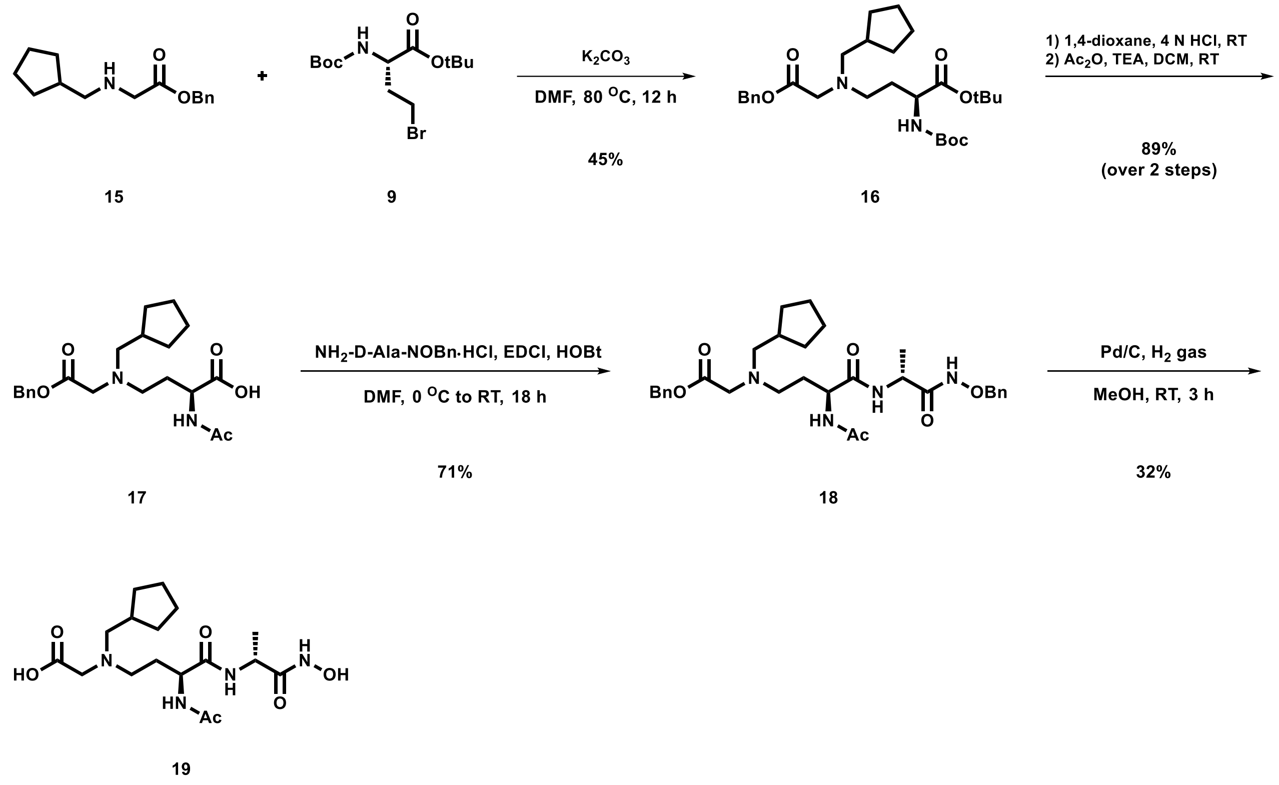
K2CO3
DMF, 80 OC, 12 h
45%
1) 1,4-dioxane, 4 N HCl, RT
2) Ac2O, TEA, DCM, RT
89%
(over 2 steps)
15
9
16
NH2-D-Ala-NOBn·HCl, EDCI, HOBt
DMF, 0 OC to RT, 18 h
71%
Pd/C, H2 gas
MeOH, RT, 3 h
32%
17
18
19

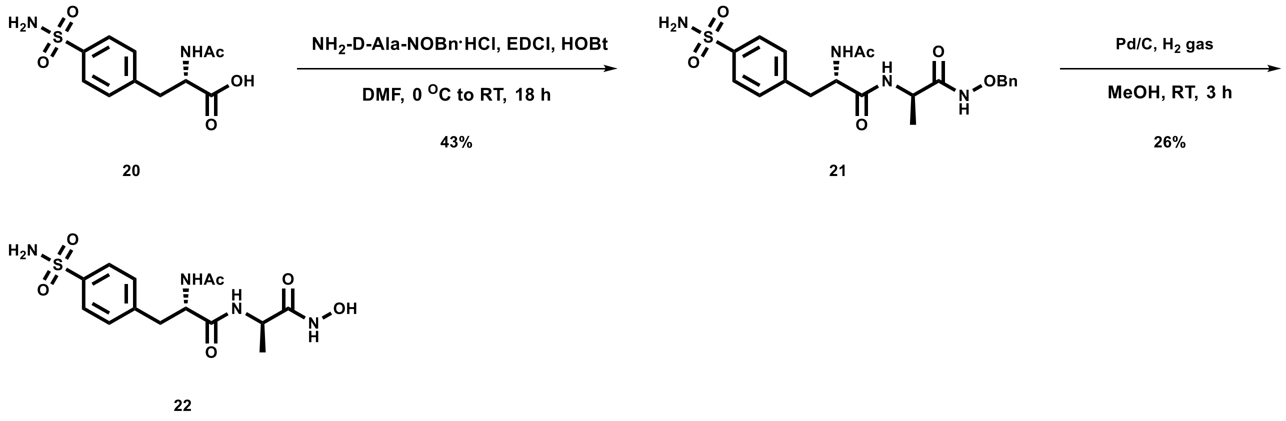
NH2-D-Ala-NOBn·HCl, EDCI, HOBt
DMF, 0 OC to RT, 18 h
43%
Pd/C, H2 gas
MeOH, RT, 3 h
26%
20
21
22

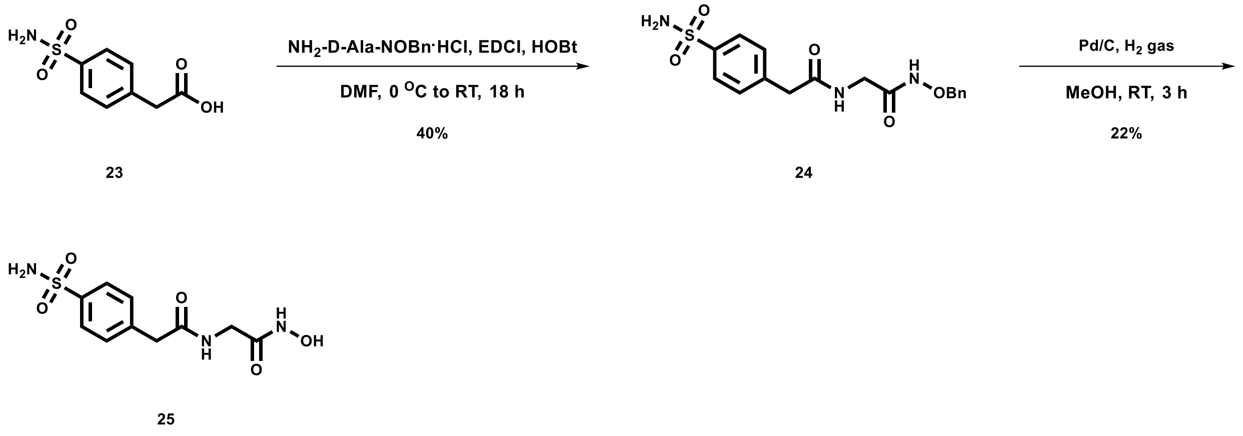
NH2-D-Ala-NOBn·HCl, EDCl, HOBt
DMF, 0 OC to RT, 18 h
40%
Pd/C, H2 gas
MeOH, RT, 3 h
22%
23
24
25

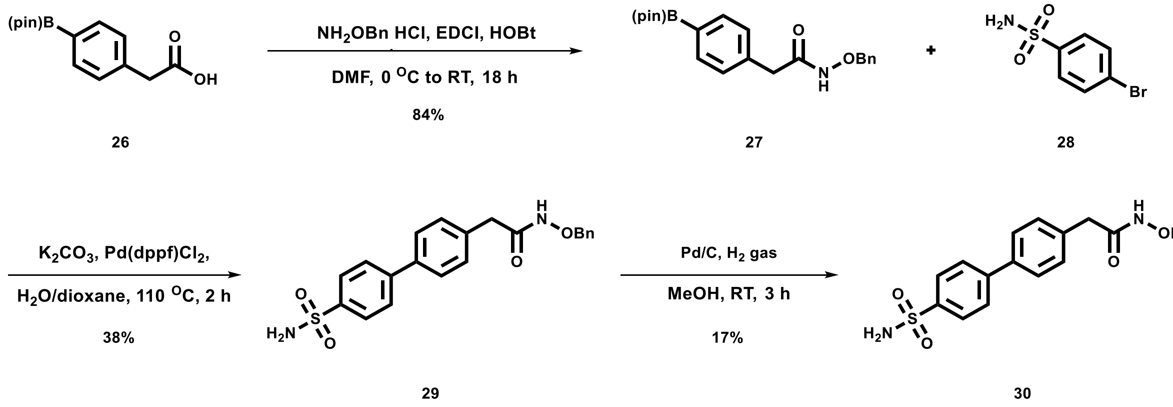
(pin)B
NH2OBn HCl, EDCl, HOBt
DMF, 0 OC to RT, 18 h
84%
26
27
28
K2CO3, Pd(dppf)Cl2,
H2O/dioxane, 110 OC, 2 h
38%
Pd/C, H2 gas
MeOH, RT, 3 h
17%
29
30

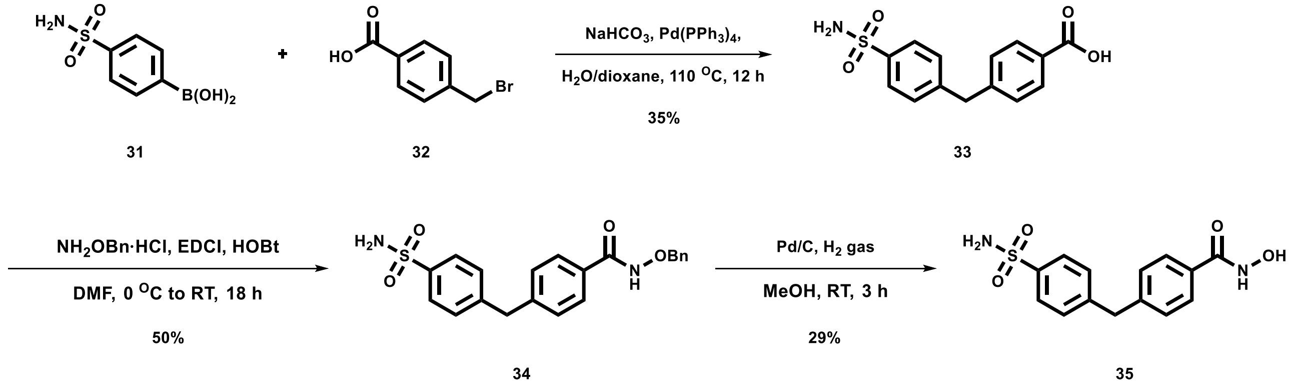
NaHCO3, Pd(PPh3)4,
H2O/dioxane, 110 OC, 12 h
35%
31
32
33
NH2OBn·HCl, EDCl, HOBt
DMF, 0 OC to RT, 18 h
50%
Pd/C, H2 gas
MeOH, RT, 3 h
29%
34
35

Experimental procedures

7-Benzyl 1-(*tert*-butyl) (2S,6R,E)-2,6-bis((*tert*-butoxycarbonyl)amino)hept-3-enedioate (3)

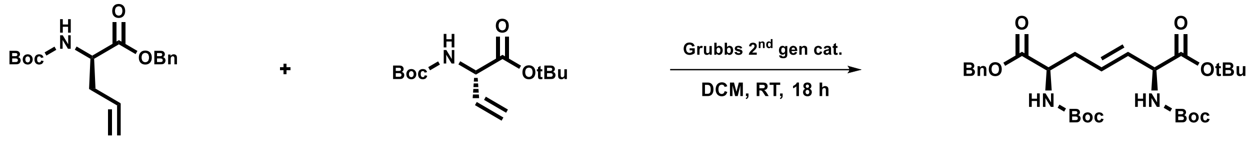

1 2 3

Experimental: A solution of (*R*)-allylglycine derivative 1 (0.31 g, 1.00 mmol) and (*S*)-vinylglycine derivative 2 (0.46 g, 1.80 mmol) in dichloromethane (4.0 mL) was placed under an argon atmosphere. Grubbs' 2nd-generation catalyst (0.041 g, 0.05 mmol) was added and the reaction mixture was stirred at room temperature for 18 h. The mixture was concentrated *in vacuo* and the residue was purified by column chromatography (hexane/ethyl acetate) to furnish compound 3 as a colorless oil (0.21 g, 40%, HPLC purity > 99%) **LC-MS** (ESI) *m*/*z*: Anal. Calc'd. for [M+H] $C_{28}H_{43}N_2O_8$: 535.3; found: 535.3.

1-Benzyl 7-(*tert*-butyl) (2*R*,6*S*)-2,6-bis((*tert*-butoxycarbonyl)amino)heptanedioate (4)

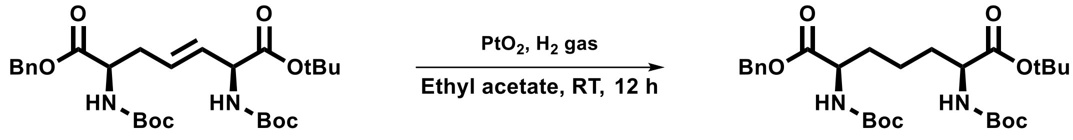

3 4

Experimental: A portion of 5% PtO_2 (51 mg) was added to a solution of compound **3** (0.24 g, 0.45 mmol) in ethyl acetate (1.1 mL). The reaction mixture was placed under a H_2 atmosphere and was stirred for 12 h. On completion of the reaction, the suspension was filtered through a pad of Celite®, the solid was washed with methanol, and the filtrate was concentrated *in vacuo*. The residue was purified by column chromatography (hexane/ethyl acetate) to furnish compound **4** as a colorless oil (0.23 g, 95%, HPLC purity > 99%). **LC-MS** (ESI) m/z: Anal. Calc'd. for [M+H] $C_{28}H_{45}N_2O_8$: 537.3; found: 537.3.

(2*S*,6*R*)-2,6-Diacetamido-7-(benzyloxy)-7-oxoheptanoic acid (5)

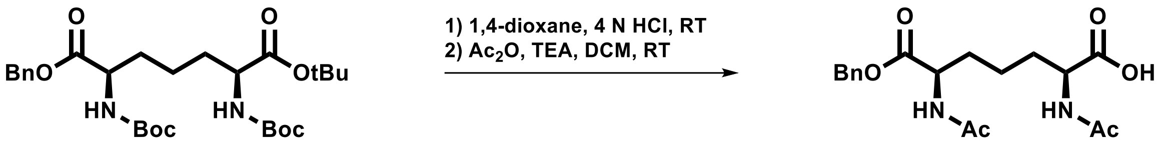

4 **5**

Experimental: To a round-bottom flask charged with compound **4** (0.20 g, 0.37 mmol) was added 4.0 N HCl/dioxane (1.2 mL). The mixture was stirred at room temperature for 6 h. The mixture was diluted with diethyl ether (20 mL) and concentrated under reduced pressure to afford the crude amine, which was used directly in the next reaction.

To a solution of the crude amine in dichloromethane (1.5 mL) was added Ac_2O (74 μL, 0.78 mmol) and TEA (217 μL, 1.55 mmol) at iced-water temperature. The reaction mixture was allowed to warm to room temperature and was stirred for 2 h. A 1.0 N HCl solution (5.0 mL) was added and solution was washed with ethyl acetate (3 x 10 mL). The combined organic layer was dried over anhydrous $MgSO_4$, the suspension was filtered and the filtrate was concentrated to dryness. The residue was purified by column chromatography (dichloromethane/methanol). The desired product was obtained as a colorless oil (0.12 g, 85%, HPLC purity > 99%). **LC-MS** (ESI) *m*/*z*: Anal. Calc'd. for [M+H] $C_{18}H_{25}N_2O_6$: 365.2; found: 365.1.

Benzyl (2*R*,6*S*)-2,6-diacetamido-7-(((*R*)-1-((benzyloxy)amino)-1-oxopropan-2-yl) amino)-7-oxoheptanoate (6)

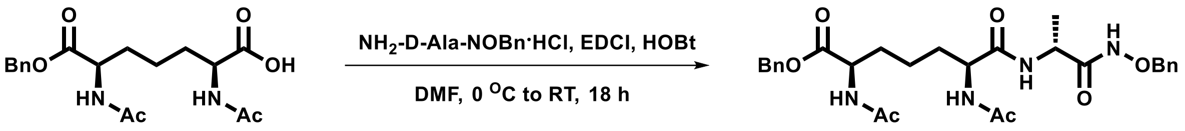

5 **6**

Experimental: To (*R*)-2-amino-*N*-(benzyloxy)propanamide hydrochloride (80 g, 0.35 mmol) in DMF (1.2 ml) was added EDCI (84 mg, 0.44 mmol), HOBt (59 mg, 0.44 mmol), TEA (0.12 ml, 0.87 mmol) and compound **5** (0.1 g, 0.29 mmol). The reaction mixture was stirred for overnight. The mixture was then poured in 1.0 N aq HCl solution (5.0 mL) and the mixture was extracted with ethyl acetate (3 x 10 mL). The organic layer was dried over anhydrous $MgSO_4$, and then the residue was purified by column chromatography (dichloromethane/methanol). The desired product

was obtained as a white solid (0.10 g, 65%, HPLC purity > 99%). **LC-MS** (ESI) *m/z*: Anal. Calc'd. for [M+H] $C_{28}H_{37}N_4O_7$: 541.3; found: 541.2.

(2*R*,6*S*)-2,6-Diacetamido-7-(((*R*)-1-(hydroxyamino)-1-oxopropan-2-yl)amino)-7-oxoheptanoic acid (7, BMK-S101)

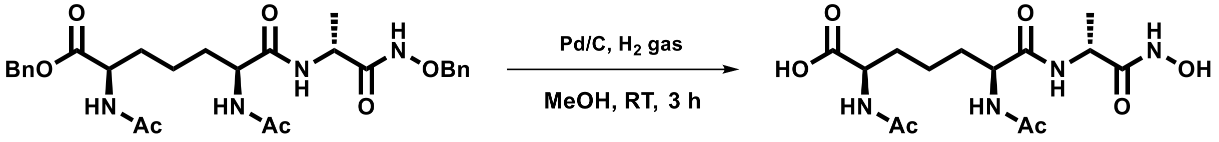

6 7

Experimental: A portion of 10% Pd on carbon (19 mg) was added to a solution of compound **6** **(**0.10 g, 0.19 mmol) in methanol (1.0 mL). The reaction mixture was placed under a H_2 atmosphere and stirred for 3 h. On completion of the reaction, the suspension was filtered through a pad of Celite®, the solid was washed with methanol, and the filtrate was concentrated *in vacuo*. The residue was purified by preparative RP-HPLC to furnish compound **7** (0.015 g, 22%, HPLC purity > 99%). **^{1}H NMR** (500 MHz, methanol-*d*$_4$) δ 4.37 – 4.18 (m, 2H), 4.22 – 4.16 (m, 1H), 1.98 (s, 6H), 1.90 – 1.80 (m, 1H), 1.78 – 1.73 (m, 1H), 1.72 – 1.61 (m, 1H) 1.53 – 1.38 (m, 2H), 1.35 (d, *J* = 7.2 Hz, 3H). **^{13}C NMR** (125 MHz, deuterium oxide) δ 175.9, 174.3, 174.1, 174.0, 171.2, 53.7, 52.6, 47.6, 30.2, 29.9, 21.5, 21.2, 16.5. **HRMS** (ESI) *m/z*: Anal. Calc'd. for [M+Na] $C_{14}H_{24}N_4NaO_7$: 383.1543; found 383.1541.

***tert*-Butyl (*S*)-4-(benzyl(2-(benzyloxy)-2-oxoethyl)amino)-2-((*tert*-butoxycarbonyl) amino) butanoate (10)**

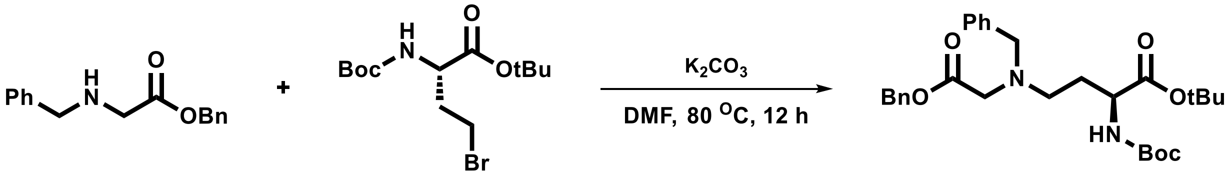

8 9 10

Experimental: In a round-bottom flask charged with the compound **8** (0.26 g, 1.00 mmol) in DMF (5.0 mL), K_2CO_3 (0.21 g 1.5 mmol) and compound **9** (0.51 g, 1.5 mmol) were added. The resulting brown suspension was stirred at 80 ºC for 12 h. After complete consumption of the compound **8**,

the reaction was quenched with 1.0 N HCl (10 mL) under cooling with ice bath. The aqueous layer was washed three times with ethyl acetate (3 x 20 mL) and the combined organic layer was washed with saturated NaCl (3 x 20 mL), dried over anhydrous $MgSO_4$. The suspension was filtered and the filtrate was concentrated under reduced pressure. The crude product was purified by column chromatography (hexane/ethyl acetate). The desired product was obtained as a white solid (0.27 g, 53%, HPLC purity > 99%). **LC-MS** (ESI) *m*/*z*: Anal. Calc'd. for [M+H] $C_{29}H_{41}N_2O_6$: 512.3; found 512.2.

(*S*)-2-Acetamido-4-(benzyl(2-(benzyloxy)-2-oxoethyl)amino)butanoic acid (11)

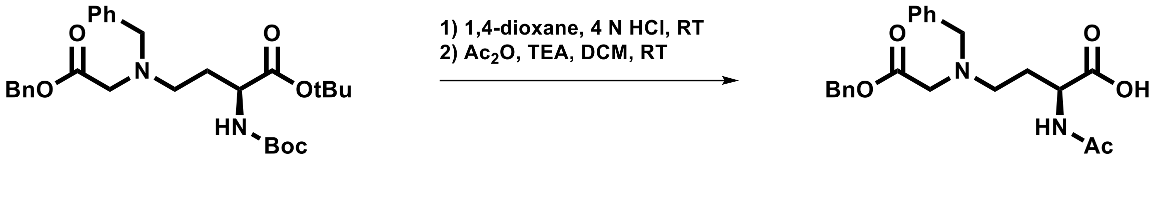

10 11

Experimental: To a round-bottom flask charged with the compound **10** (0.20 g, 0.39 mmol) was added 4.0 N HCl/dioxane (1.2 mL). The mixture was stirred at room temperature for 6 h. The mixture was diluted with diethyl ether (20 mL) and was concentrated under reduced pressure to afford the resultant crude amine, which was used directly in the next reaction.

To a solution of the crude amine in dichloromethane (1.5 mL) was added Ac_2O (44 µL, 0.47 mmol) and TEA (130 µL, 0.94 mmol) at iced-water temperature. The reaction mixture was allowed to warm to room temperature and was stirred 2 h. A 1.0 N HCl solution (5.0 mL) was added and the mixture was washed with ethyl acetate (3 x 10 mL), and the combined organic layer was dried over anhydrous $MgSO_4$. The suspension was filtered and the filtrate was concentrated to dryness. The residue was purified with column chromatography (dichloromethane/methanol). The desired product was obtained as a colorless oil (0.14 g, 91%, HPLC purity > 99%). **LC-MS** (ESI) *m*/*z*: Anal. Calc'd. for [M+H] $C_{22}H_{27}N_2O_5$: 399.2; found 399.1.

Benzyl *N*-((*S*)-3-acetamido-4-oxo-4-(((*R*)-1-oxo-1-((trityloxy)amino)propan-2-yl) amino)butyl)-N-benzylglycinate (12)

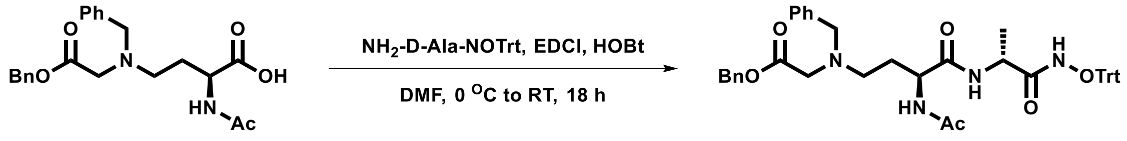

11 12

Experimental: To (*R*)-2-amino-*N*-(trityloxy)propanamide (0.087 g, 0.25 mmol) in DMF (1.0 mL) was added EDCI (0.058 g, 0.30 mmol), HOBt (0.040 g, 0.30 mmol), TEA (0.087 ml, 0.63 mmol) and compound **11** (0.10 g, 0.25 mmol). The reaction mixture was stirred overnight. The mixture was then poured into 1.0 N HCl (5.0 mL) and the solution was washed with ethyl acetate (3 x 10 mL). The combined organic layer was dried over anhydrous $MgSO_4$, the suspension was filtered and the filtrate was evaporated to dryness. The residue was purified by column chromatography (dichloromethane/methanol). The desired product was obtained as a white solid (0.10 g, 55%, HPLC purity > 99%). **LC-MS** (ESI) *m/z*: Anal. Calc'd. for [M+H] $C_{44}H_{47}N_4O_6$: 727.3; found 727.3.

_N_-((_S_)-3-Acetamido-4-(((_R_)-1-(hydroxyamino)-1-oxopropan-2-yl)amino)-4-oxobutyl)-_N_-benzylglycine (13, BMK-S201)

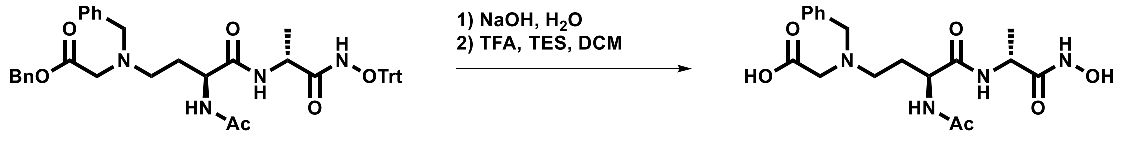

12 13

Experimental: To a stirred solution of compound **12** (0.10 g, 0.14 mmol) in water (1.0 mL) was added NaOH (0.033 g, 0.83 mmol). The reaction was stirred for 2 h at room temperature. The mixture was then poured in 1.0 N HCl solution (5.0 mL) under cooling with ice bath and was washed with ethyl acetate (3 x 10 mL). The combined organic layer was dried over anhydrous $MgSO_4$, the suspension was filtered and the filtrate was concentrated under reduced pressure to furnish the corresponding carboxylic acid, which was used directly in the next reaction.

Following a reported procedure[1], the corresponding carboxylic acid was dissolved in dichloromethane (1.5 mL), and TFA (38 µL) and triethylsilane (38 µL) were added. The mixture was stirred at room temperature for 30 min, solvents were removed under reduced pressure and the crude mixture was washed with hexane/diethyl ether 75:25 (v:v) affording a residue. The corresponding residue was purified by preparative RP-HPLC to obtain compound **13** (0.016 g,

30%, HPLC purity > 99%). **^{1}H NMR** (500 MHz, deuterium oxide) δ 7.50 (s, 5H), 4.42 (s, 2H), 4.34 (s, 1H), 4.24 – 4.18 (m, 1H), 3.79 (s, 2H), 3.24 (s, 2H), 2.17 (d, *J* = 74.1 Hz, 2H), 1.98 (s, 3H), 1.33 (d, *J* = 6.8 Hz, 3H). **^{13}C NMR** (125 MHz, Deuterium Oxide) δ 174.3, 171.8, 171.1, 130.9, 130.3, 129.3, 128.6, 58.6, 54.8, 50.9, 50.4, 47.7, 25.8, 21.6, 16.4. **HRMS** (ESI) *m/z*: Anal. Calc'd. For [M+Na] $C_{18}H_{26}N_4NaO_6$: 417.1750; found 417.1749.

((*S*)-3-Acetamido-4-(((*R*)-1-(hydroxyamino)-1-oxopropan-2-yl)amino)-4-oxobutyl) glycine (14, BMK-S202)

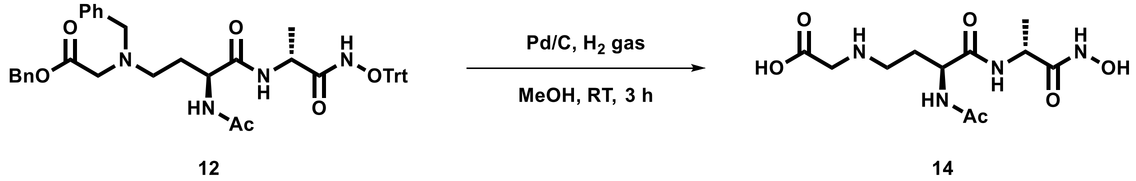

Experimental: A portion of 10% Pd on carbon (11 mg) was added to a solution of compound **12** (0.070 g, 0.10 mmol) in methanol (1.0 mL). The reaction mixture was placed under a hydrogen atmosphere and was stirred for 3 h. On completion of the reaction, the suspension was filtered through a pad of Celite®, the solid was washed with MeOH, and the filtrate was concentrated *in vacuo*. The residue was purified by preparative RP-HPLC to furnish compound **14** (0.012 g, 40%, HPLC purity > 99%). **^{1}H NMR** (500 MHz, methanol-*d*$_4$) δ 4.45 (t, *J* = 6.5 Hz, 1H), 4.29 (d, *J* = 7.4 Hz, 1H), 3.82 (s, 2H), 3.11 (s, 2H), 2.18 (s, 1H), 2.05 (s, 3H), 2.00 (s, 1H), 1.42 – 1.31 (m, 3H). **^{13}C NMR** (125 MHz, Deuterium Oxide) δ 174.7, 172.3, 171.3, 170.1, 51.3, 48.5, 47.8, 44.2, 27.6, 21.8, 16.6. **HRMS** (ESI) *m/z*: Anal. Calc'd. for [M+Na] $C_{11}H_{20}N_4NaO_6$: 327.1281; found 327.1279.

***tert*-Butyl (*S*)-4-((2-(benzyloxy)-2-oxoethyl)(cyclopentylmethyl)amino)-2-((*tert*-butoxycarbonyl)amino)butanoate (16)**

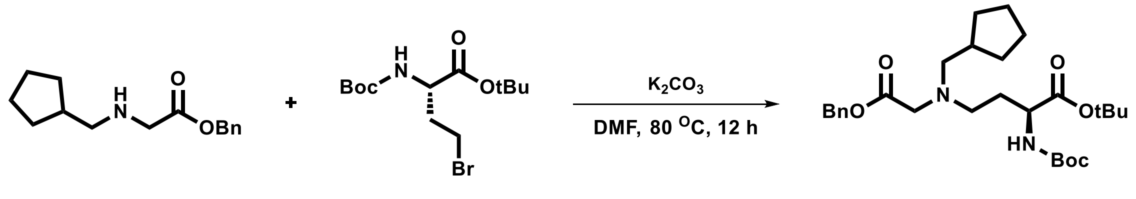

15 9 16

Experimental: In a round-bottom flask charged with compound **15** (0.25 g, 1.00 mmol) in DMF (5.0 mL), K_2CO_3 (0.21 g 1.5 mmol) and compound **9** (0.51 g, 1.5 mmol) were added. The resulting brown suspension was stirred at 80 ºC for 12 h. After complete consumption of **15**, the reaction was quenched with 1.0 N HCl (10 mL) under cooling with ice bath. The aqueous layer was washed three times with ethyl acetate (3 x 20 mL) and the combined organic layer was washed with saturated NaCl (3 x 20 mL), dried over anhydrous $MgSO_4$. The suspension was filtered and the filtrate was concentrated under reduced pressure to dryness. The crude product was purified by column chromatography (hexane/ethyl acetate). The desired product was obtained as a white solid (0.21 g, 41%, HPLC purity > 99%). **LC-MS** (ESI) *m/z*: Anal. Calc'd. for [M+H] $C_{28}H_{45}N_2O_6$: 505.3; found 505.3.

(*S*)-2-Acetamido-4-((2-(benzyloxy)-2-oxoethyl)(cyclopentylmethyl)amino)butanoic acid (17)

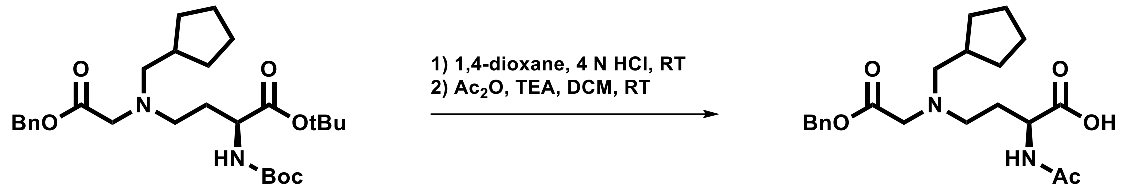

16 17

Experimental: To a round-bottom flask charged with compound **16** (0.20 g, 0.40 mmol) was added 4.0 N HCl/dioxane (1.2 mL). The mixture was stirred at room temperature for 6 h. The mixture was diluted with diethyl ether (20 mL) and was concentrated under reduced pressure to afford the corresponding crude amine, which was used directly in the next reaction.

To a solution of crude amine in dichloromethane (1.5 mL) was added Ac_2O (44 µL, 0.47 mmol) and Et_3N (130 µL, 0.94 mmol) at iced-water temperature. The reaction mixture was allowed to warm to room temperature and was stirred 2 h. A portion of 1.0 N HCl (5.0 mL) was added and the mixture was washed with ethyl acetate (3 x 10 mL), and the combined organic layer was dried over anhydrous $MgSO_4$. The suspension was filtered and the filtrate was concentrated to dryness. The residue was purified by column chromatography (dichloromethane/methanol). The desired product was obtained as a colorless oil (0.14 g, 89%, HPLC purity > 99%). **LC-MS** (ESI) *m/z*: Anal. Calc'd. for [M+H] $C_{21}H_{31}N_2O_5$: 391.2; found 391.2.

Benzyl N-((S)-3-acetamido-4-(((R)-1-((benzyloxy)amino)-1-oxopropan-2-yl)amino)-4-oxobutyl)-N-(cyclopentylmethyl)glycinate (18)

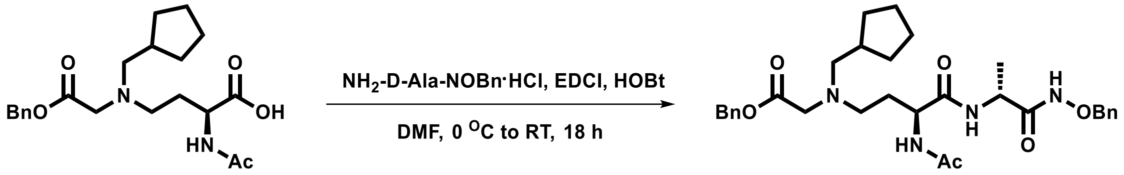

17 18

Experimental: To (*R*)-2-amino-*N*-(benzyloxy)propanamide hydrochloride (0.045 g, 0.25 mmol) in DMF (1.0 mL) was added EDCI (0.058 g, 0.30 mmol), HOBt (0.04 g, 0.30 mmol), TEA (0.087 ml, 0.63 mmol) and compound **17** (0.1 g, 0.25 mmol). The reaction mixture was stirred overnight. The mixture was then poured into 1.0 N HCl solution (5.0 mL) and was washed with ethyl acetate (3 x 10 mL). The combined organic layer was dried over anhydrous $MgSO_4$, the suspension was filtered and the filtrate was concentrated to dryness. The residue was purified by column chromatography (hexane/ethyl acetate). The desired product was obtained as a white solid (0.10 g, 71%, HPLC purity > 99%). **LC-MS** (ESI) *m/z*: Anal. Calc'd. for [M+H] $C_{31}H_{43}N_4O_6$: 567.3; found 589.3.

N-((S)-3-Acetamido-4-(((R)-1-(hydroxyamino)-1-oxopropan-2-yl)amino)-4-oxobutyl) -N-(cyclopentylmethyl)glycine (19, BMK-S203)

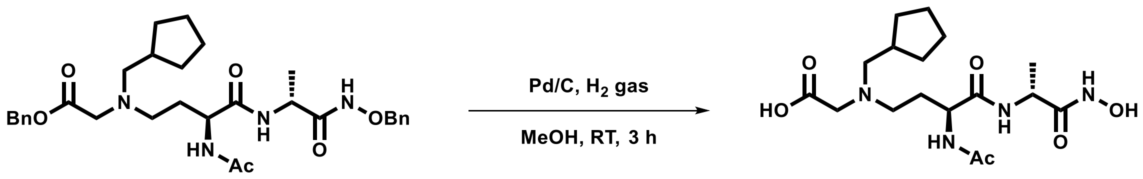

18 19

Experimental: A portion of 10% Pd on carbon (0.013 g) was added to a solution of compound **18** (0.070 g, 0.12 mmol) in MeOH (1.0 mL). The reaction mixture was placed under a H_2 atmosphere and stirred for 3 h. On completion of the reaction, the suspension was filtered through a pad of Celite®, the solid was washed with MeOH, and the filtrate was concentrated *in vacuo* to dryness. The residue was purified by preparative RP-HPLC to obtain compound **19**(0.015 g, 32%, HPLC purity > 99%). ^{1}H NMR (500 MHz, methanol-d_4) δ 4.46 (t, *J* = 6.5 Hz, 1H), 4.30 (q, *J* = 7.0 Hz, 1H), 4.02 (s, 2H), 3.33 (t, *J* = 7.3 Hz, 2H), 3.23 (t, *J* = 5.6 Hz, 2H), 2.30 – 2.21 (m,, 2H), 2.02 (s,

3H), 1.97 – 1.88 (m,, 2H), 1.75 – 1.69 (m,, 2H), 1.67 – 1.59 (m,, 2H), 1.40 – 1.28 (m,, 5H). ^{13}C NMR (125 MHz, Deuterium oxide) δ 174.4, 171.9, 171.1, 169.0, 54.5, 51.5, 51.0, 47.7, 34.8, 30.4, 25.4. 24.5, 21.6, 16.4. **HRMS** (ESI) *m/z*: Anal. Calc'd. for [M+Na] $C_{17}H_{30}N_4NaO_6$: 409.2063; found 409.2062.

(*S*)-2-Acetamido-*N*-((*R*)-1-((benzyloxy)amino)-1-oxopropan-2-yl)-3-(4-sulfamoylphenyl)propenamide (21)

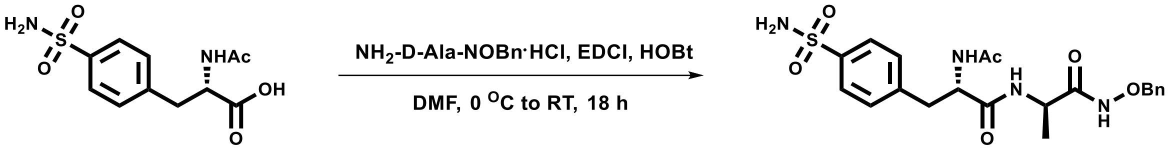

20 21

Experimental: To (*R*)-2-amino-*N*-(benzyloxy)propanamide hydrochloride (0.28 g, 1.2 mmol) in DMF (4.0 mL) was added EDCI (0.29 g, 1.5 mmol), HOBt (0.20 g, 1.5 mmol), TEA (0.35 ml, 2.5 mmol) and *N*-acetyl-4-(aminosulfonyl)-L-phenylalanine (0.29 g, 1.0 mmol). The reaction mixture was stirred overnight. The mixture was then poured into 1.0 N aq HCl solution (20 mL) and the mixture was washed with ethyl acetate (3 x 20 mL). The combined organic layer was dried over anhydrous $MgSO_4$, the suspension was filtered and the filtrate was concentrated to dryness. The residue was purified by column chromatography (dichloromethane/methanol). The desired product was obtained as a white solid (0.20 g, 43%, HPLC purity > 99%). MS (ESI) *m/z*: Anal. Calc'd. for [M+H] $C_{21}H_{27}N_4O_6S$: 463.2; found 463.1.

(*S*)-2-Acetamido-*N*-((*R*)-1-(hydroxyamino)-1-oxopropan-2-yl)-3-(4-sulfamoylphenyl) propenamide (22, BMK-S301)

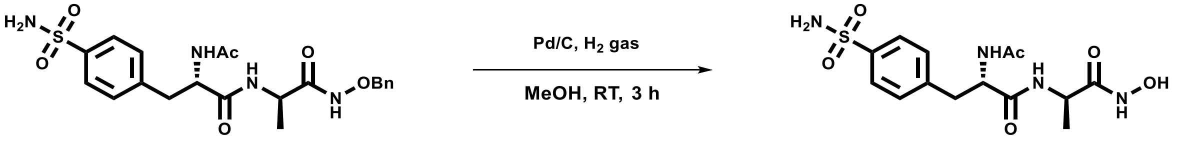

21 22

Experimental: A portion of 10% Pd on carbon (23 mg) was added to a solution of compound **21** (0.10 g, 0.22 mmol) in methanol (1.0 mL). The reaction mixture was stirred for 3 h under hydrogen atmosphere. On completion of the reaction, the suspension was filtered through a pad of Celite®, the solid was washed with methanol, and the filtrate was concentrated *in vacuo* to dryness. The

residue was purified by preparative RP-HPLC to obtain compound **22** (0.021 g, 26%, HPLC purity > 99%). **^{1}H NMR** (500 MHz, deuterium oxide) δ 7.88 (d, *J* = 7.6 Hz, 2H), 7.47 (d, *J* = 7.7 Hz, 2H), 4.52 (t, *J* = 7.6 Hz, 1H), 4.15 (d, *J* = 7.1 Hz, 1H), 3.22 – 3.07 (m, 2H), 1.99 (s, 3H), 1.12 (d, *J* = 6.9 Hz, 3H). **^{13}C NMR** (125 MHz, Deuterium oxide) δ 174.1, 172.6, 171.1, 142.0, 140.0, 130.3, 126.2, 55.1, 47.5, 37.0, 21.6, 16.3. **HRMS** (ESI) *m*/*z*: Anal. Calc'd. for [M+Na] $C_{14}H_{20}N_4NaO_6S$: 395.1001; found 395.0998.

N-(Benzyloxy)-2-(2-(4-sulfamoylphenyl)acetamido)acetamide (24)

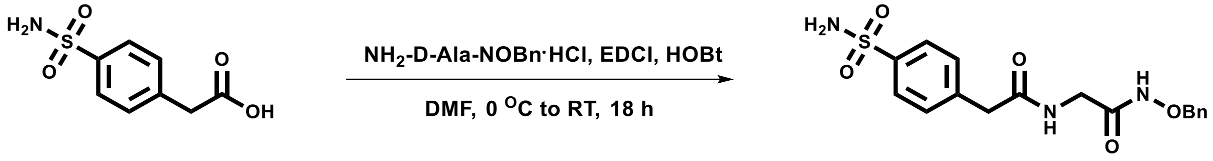

23 24

Experimental: To 2-amino-*N*-(benzyloxy)acetamide hydrochloride (0.28 g, 1.2 mmol) in DMF (4.0 mL) was added EDCI (0.29 g, 1.5 mmol), HOBt (0.20 g, 1.5 mmol), TEA (0.35 ml, 2.5 mmol) and 4-(aminosulfonyl)benzeneacetic acid (0.22 g, 1.0 mmol). The reaction mixture was stirred overnight. The mixture was then poured into 1.0 N aq HCl solution (20 mL) and the resulting mixture was washed with ethyl acetate (3 x 20 mL). The combined organic layer was dried over anhydrous $MgSO_4$, the suspension was filtered and the filtrate was concentrated to dryness. The residue was purified with the column chromatography (dichloromethane/methanol). The desired product was obtained as a white solid (0.15 g, 40%, HPLC purity > 99%). **LC-MS** (ESI) *m*/*z*: Anal. calcd. for [M+H] $C_{17}H_{20}N_3O_5S$: 378.1; found 378.1.

N-Hydroxy-2-(2-(4-sulfamoylphenyl)acetamido)acetamide (25, BMK-S302)

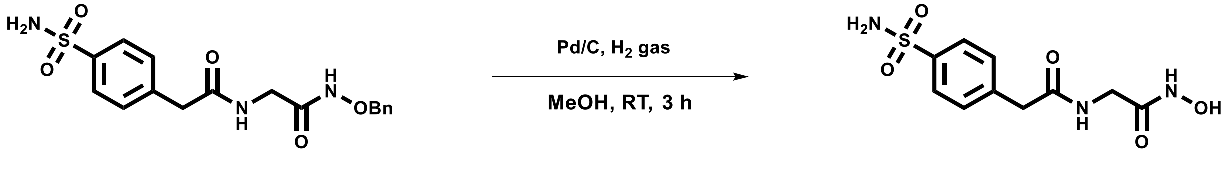

24 25

Experimental: A portion of 10% Pd on carbon (28 mg) was added to a solution of compound **24** (0.1 g, 0.26 mmol) in MeOH (1.0 mL). The reaction mixture was placed stirred for 3 h under hydrogen atmosphere. On completion of the reaction, the suspension was filtered through a pad of

Celite®, the solids were washed with MeOH, and the filtrate was concentrated *in vacuo* to dryness. The residue was purified by preparative RP-HPLC to obtain compound **25** (0.016 g, 22%, HPLC purity > 99%). **^{1}H NMR** (500 MHz, methanol-d_4) δ 7.84 (d, J = 7.9 Hz, 2H), 7.48 (d, J = 7.9 Hz, 2H), 3.80 (s, 1H), 3.68 (s, 1H), 3.35 (s, 2H). **^{13}C NMR** (125 MHz, Methanol-d_4) δ 173.5, 167.1, 143.6, 141.3, 130.9, 127.3, 43.1, 41.4. **HRMS** (ESI) *m*/*z*: Anal. Calc'd. for [M+Na] $C_{10}H_{13}N_3NaO_5S$: 310.0474; found 310.0516.

_N_-(Benzyloxy)-2-(4-(4,4,5,5-tetramethyl-1,3,2-dioxaborolan-2-yl)phenyl)acetamide (27)

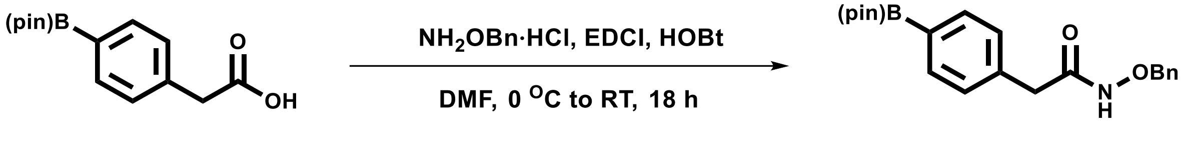

26 27

Experimental: To *O*-benzyl hydroxylamine hydrochloride (0.19 g, 1.2 mmol) in DMF (4.0 mL) was added EDCI (0.29 g, 1.5 mmol) and HOBt (0.20 g, 1.5 mmol), TEA (0.35 ml, 2.5 mmol) and compound **15** (0.26 g, 1.0 mmol) and the reaction mixture was stirred overnight. The mixture was then poured into 1.0 N HCl solution (20 mL) and was washed with EtOAc (3 x 20 mL). The combined organic layer was dried over anhydrous $MgSO_4$, the suspension was filtered and the filtrate was concentrated to dryness. The residue was purified by column chromatography (hexane/ethyl acetate). The desired product was obtained as a white solid (0.31 g, 84%, HPLC purity > 99%). **LC-MS** (ESI) *m*/*z*: Anal. Calc'd. for [M+H] $C_{28}H_{37}N_4O_7$: 368.2; found 368.2.

_N_-(Benzyloxy)-2-(4'-sulfamoyl-[1,1'-biphenyl]-4-yl)acetamide (29)

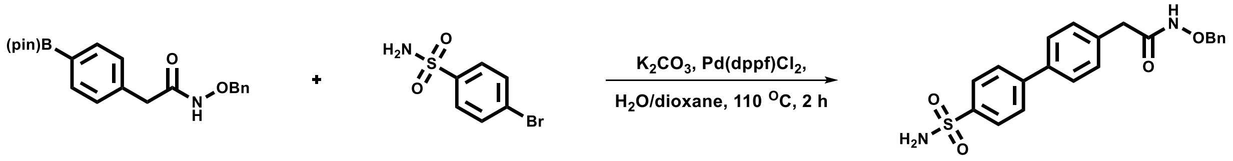

27 28 29

Experimental: A two-necked round-bottom flask was charged with 4-bromobenzenesulfonamide (**28**) (0.24 g, 1.0 mmol), compound **27** (0.44 g, 1.2 mmol), [1,1'-bis(diphenylphosphino)ferrocene] dichloropalladium(II) DCM complex (40 mg, 0.050 mmol), and potassium carbonate (0.21 g, 1.5 mmol). The flask was purged with nitrogen, followed by the addition of 1,4-dioxanes (5 mL) and water (0.25 mL). The mixture was brought to reflux for a 2-h duration. The mixture was then

poured into 1.0 N HCl (20 mL) and was washed with ethyl acetate (3 x 20 mL). The combined organic layer was dried over anhydrous $MgSO_4$, the suspension was filtered and the filtrate was concentrated to dryness. The crude residue was purified by column chromatography (dichloromethane/methanol). The title compound was obtained as a white solid (0.10 g, 38%, HPLC purity > 99%). **LC-MS** (ESI) *m/z*: Anal. Calc'd. for [M+H] $C_{18}H_{25}N_2O_6$: 397.1; found 397.1.

_N_-Hydroxy-2-(4'-sulfamoyl-[1,1'-biphenyl]-4-yl)acetamide (30, BMK-S303)

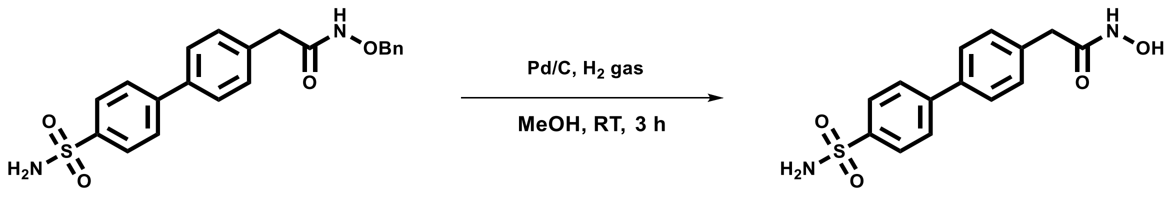

29 30

Experimental: A portion of 10% Pd on carbon (27 mg) was added to a solution of compound **29** (0.10 g, 0.25 mmol) in MeOH (1.0 mL). The reaction mixture was placed stirred for 3 h under hydrogen atmosphere. On completion of the reaction, the suspension was filtered through a pad of Celite®, the solid was washed with methanol, and the filtrate was concentrated *in vacuo*. The residue was purified by preparative RP-HPLC to obtain compound **30** (0.013 g, 17%, HPLC purity > 99%). ^{1}H NMR (500 MHz, acetonitrile-d_3) δ 7.91 (d, J = 7.9 Hz, 2H), 7.79 (d, J = 8.2 Hz, 2H), 7.63 (d, J = 7.8 Hz, 2H), 7.38 (d, J = 7.7 Hz, 2H), 3.42 (s, 2H). 13C NMR (126 MHz, DMSO-d6) δ 166.8, 143.2, 142.8, 136.9, 136.4, 129.7, 127.0, 126.9, 126.3, 39.0. **HRMS** (ESI) *m/z*: Anal. Calc'd. For [M+Na] $C_{14}H_{14}N_2NaO_4S$: 329.0572.; found 329.0577.

4-(4-Sulfamoylbenzyl)benzoic acid (32)

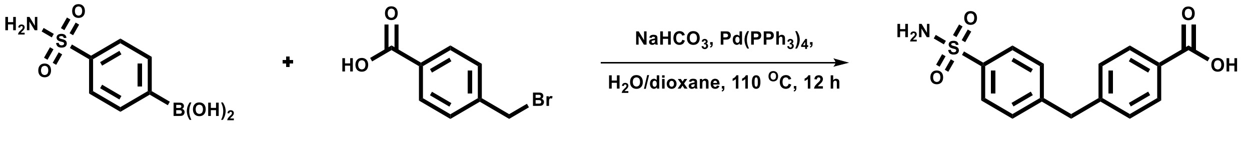

31 32 33

Experimental: To a solution of compound **31** (0.21 g, 1.0 mmol) in 1,2-dimethoxyethane (2.0 mL) and water (1.0 mL) was added compound **32** (0.24 g, 1.2 mmol), sodium bicarbonate (0.18 g, 2.1 mmol) and tetrakis(triphenylphosphine) palladium(0) (0.011 g, 0.010 mmol). The flask

containing the mixture was vacuum flushed and filled with nitrogen and then heated to 100 °C for 12 h. The mixture was then poured into 1.0 N HCl (20 mL) and was washed with dichloromethane (3 x 20 mL). The combined organic layer was dried over anhydrous $MgSO_4$, the suspension was filtered and the filtrate was concentrated to dryness. Solvent was removed and the residue was purified by column chromatography (dichloromethane/methanol) to obtain compound **33** (0.1 g, 35%, HPLC purity > 99%). **LC-MS** (ESI) *m/z*: Anal. Calc'd. For [M+H] $C_{18}H_{25}N_2O_6$: 292.1; found 292.0.

***N*-(Benzyloxy)-4-(4-sulfamoylbenzyl)benzamide (34)**

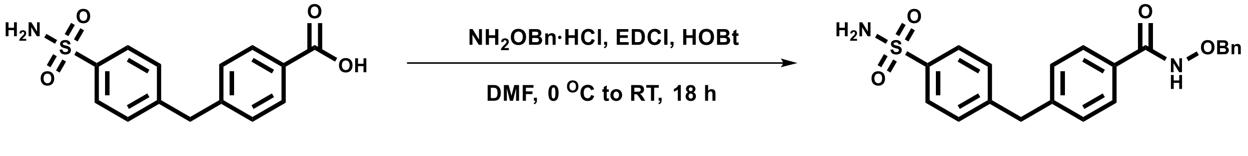

33 34

Experimental: To *O*-benzyl hydroxylamine hydrochloride (0.099 g, 0.62 mmol) in DMF (2.0 mL) was added EDCI (0.15 g, 0.77 mmol), HOBt (0.10 g, 0.77 mmol), TEA (0.18 ml, 1.3 mmol) and compound **33** (0.15 g, 0.52 mmol). The reaction mixture was stirred overnight. The mixture was then poured into 1.0 N HCl solution (10 mL) and was washed with ethyl acetate (3 x 20 mL). The combined organic layer was dried over anhydrous $MgSO_4$, the suspension was filtered and the filtrate was concentrated to dryness. The residue was purified by column chromatography (dichloromethane/methanol). The title compound was obtained as a white solid (0.10 g, 50%, HPLC purity > 99%). **LC-MS** (ESI) *m/z*: Anal. Calc'd. For [M+H] $C_{28}H_{37}N_4O_7$: 397.1; found 397.1.

***N*-Hydroxy-4-(4-sulfamoylbenzyl)benzamide (35, BMK-S304)**

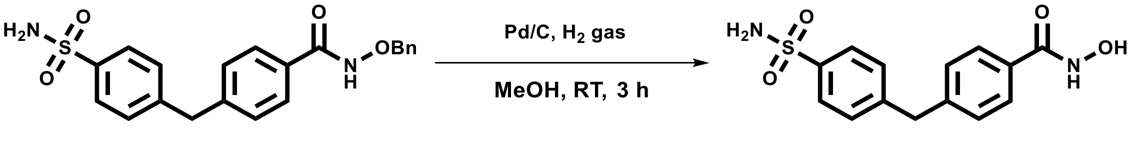

34 35

Experimental: A portion of 10% Pd on carbon (27 mg) was added to a solution of compound **34** (0.10 g, 0.25 mmol) in methanol (1.0 mL). The reaction mixture was placed was stirred for 3 h under hydrogen atmosphere. On completion of the reaction, the suspension was filtered through a pad of Celite®, the solid was washed with methanol, and the filtrate was concentrated to dryness

in vacuo. The residue was purified by preparative RP-HPLC to obtain compound **35** (0.022 g, 29%, HPLC purity > 99%). **^{1}H NMR** (500 MHz, methanol-*d*4) δ 7.82 (d, *J* = 8.1 Hz, 2H), 7.68 (d, *J* = 7.9 Hz, 2H), 7.39 (d, *J* = 8.0 Hz, 2H), 7.31 (d, *J* = 7.9 Hz, 2H), 4.11 (s, 2H). **^{13}C NMR** (125 MHz, methanol-*d*4) δ 166.6, 145.3, 144.4, 141.6, 130.3, 129.1, 128.8, 127.04, 126.0, 40.8. **HRMS** (ESI) *m*/*z*: Anal. Calc'd. for [M+Na] $C_{14}H_{14}N_2NaO_4S$: 329.0572.; found 329.0577.

Preparation of FRET-labeled peptide substrate

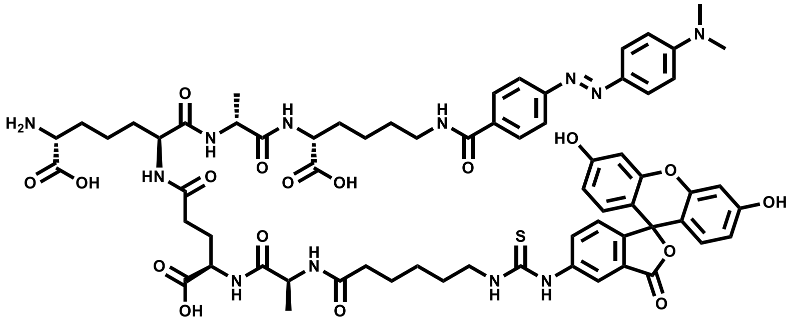

Experimental: The FRET-labeled peptide substrate was synthesized under standard Fmoc solid-phase protocol. Wang resin (50 mg, 0.078 mmol, 1.56 mmol g $^{-1}$) was preswollen with DCM for 30 min and then filtered off. Meanwhile, a solution of Fmoc-D-Lys(Dabcyl)-OH (5.0 equiv.), DIC (5.0 equiv.), HOBt (5.0 equiv.), and DMAP (0.30 equiv.) in DCM: DMF (2:1, 1.0 mL) was prepared and stirred for 5 min. The preactivated solution was added to the resin and the reaction mixture was shaken for 12 h. The resin suspension was filtered and the resin was washed with DMF (3 × 5 mL), MeOH (3 × 5 mL) and DCM (3 × 5 mL). The reaction completeness on the resin was confirmed by the Kaiser test. Then the Fmoc protective group was removed with a 20% solution of piperidine in DMF (3 × 10 min) and then washed with DMF (3 × 5 mL) and DCM (3 × 5 mL). The resin was subsequently treated with a solution of Fmoc-D-Ala-OH (5 equiv.), HBTU (5 equiv.), HOBt (5.0 equiv.), and DIPEA (10 equiv.) in DMF (1.0 mL). The reaction mixture was shaken for 90 min. The resin suspension was filtered and the resin was washed with DMF (3 × 5 mL) and DCM (3 × 5 mL). The protocols for Fmoc deprotection and HBTU coupling were repeated with the *m*-DAP derivative, Fmoc-D-Glu-OtBu, Fmoc-L-Ala-OH and Fmoc-6-Ahx-OH. At this point, a solution of FITC (1.1 equiv.) in pyridine/DMF/DCM (12:7:5, 1.0 mL) was added to the Fmoc deprotected resin and shaking of the mixture was sustained for 9 h. The resin was then filtered and washed sequentially with DMF (3 × 5 mL), MeOH (3 × 5 mL) and DCM (3 × 5 mL).

The peptide was cleaved from the resin by shaking in a solution of TFA/TIPS/H_2O (1.0 mL, 95:2.5:2.5) for 2 h. The resin was filtered, washed with TFA (7 mL) and the combined filtrate was concentrated *in vacuo*. The resulting residue was precipitated by mixing with cold ether (15 mL) to obtain a white product. The suspension was then centrifuged and the cold ether was carefully removed. The precipitated peptide was dissolved in water (10 mL) and lyophilized to furnish a white powder, which was purified by preparative RP-HPLC (7.1 mg, 15% overall yield). **LC-MS** (ESI) *m/z*: Anal. Calc'd. for [M+H] $C_{66}H_{79}N_{12}O_{17}S$: 1344.5; found 1344.5.

Synthesis of the membrane-sensitizing peptide, KL-L9P

Synthesis of KL-L9P (sequence: KLLKLLKKPLKLLK) was performed by using an Fmoc-based solid-phase peptide synthesis protocol on a Discover SPS Microwave Peptide Synthesizer (CEM). Rink Amide MBHA resin (300 mg, 0.18 mmol, 0.54 mmol/g loading capacity) was deprotected with 20% piperidine in *N*,*N*-dimethylformamide (DMF) (4 mL). Then, the first amino acid with the Fmoc-protecting group, F-moc lysine (379 mg, 0.809 mmol), benzotriazole-1-yloxy-tris-pyrrolidino-phosphonium hexafluorophosphate (PYBOP) (422 mg, 0.809 mmol) and *N*,*N*-diisopropylethylamine (DIPEA) were added to the resin and the mixture was stirred at room temperature for 5 min. The resin was washed with DMF several times. The Fmoc-protecting group from the resin charged amino acid was deprotected with 20% piperidine in DMF, followed by additional washes with DMF. The coupling and deprotection steps were repeated with different Fmoc-protected amino acids sequentially until deprotection of the last Fmoc-protected amino acids. The coupling time was generally 5 min except for the proline, which was 10 min. After the deprotection of the last amino acid, the resin-bound peptide was washed with DMF to remove piperidine. The *N*-terminus of the peptide was then acetylated by adding a solution of acetic anhydride (0.90 mmol, 87 μL) and *N*-hydroxybenzotriazole (0.90 mmol, 12 mg) in a mixed solvent (9:1 DMF:dichloromethane; 3 mL). After 2 h reaction at room temperature, the peptide was cleaved from the resin by treatment with a mixture of trifluoracetic acid (TFA), triisopropylsilane and water (96.5:1:2.5, 2 mL) for 2 h at room temperature. The resin was thoroughly washed with TFA. The cleaved peptide solution was concentrated by evaporation of the solvent with nitrogen. After precipitation into the mixture of *n*-hexane and diethyl ether (v/v = 50:50), the resulting suspension was centrifuged at 4,000 rpm for 5 min at 4 °C to collect the white pellet of the peptide.

The purity (>95%) was determined by HPLC and MALDI-TOF confirmed the molecular mass of the sample, respectively.

NMR Spectra

Compound 7

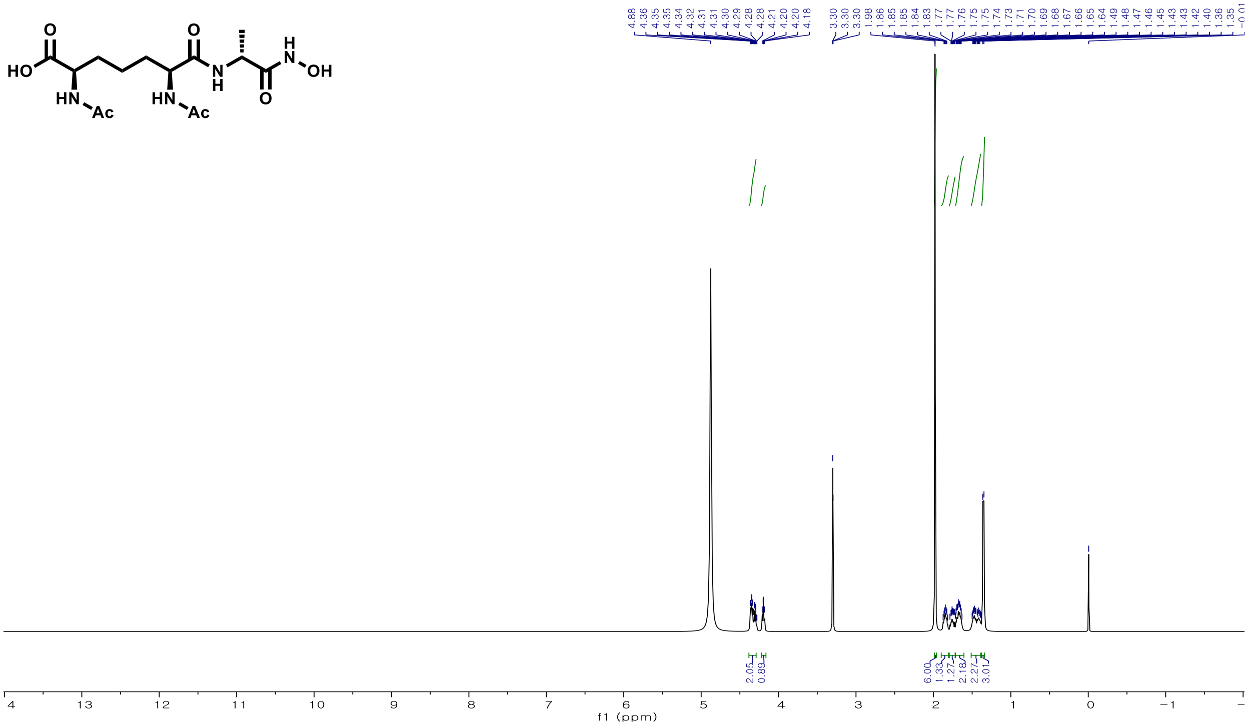

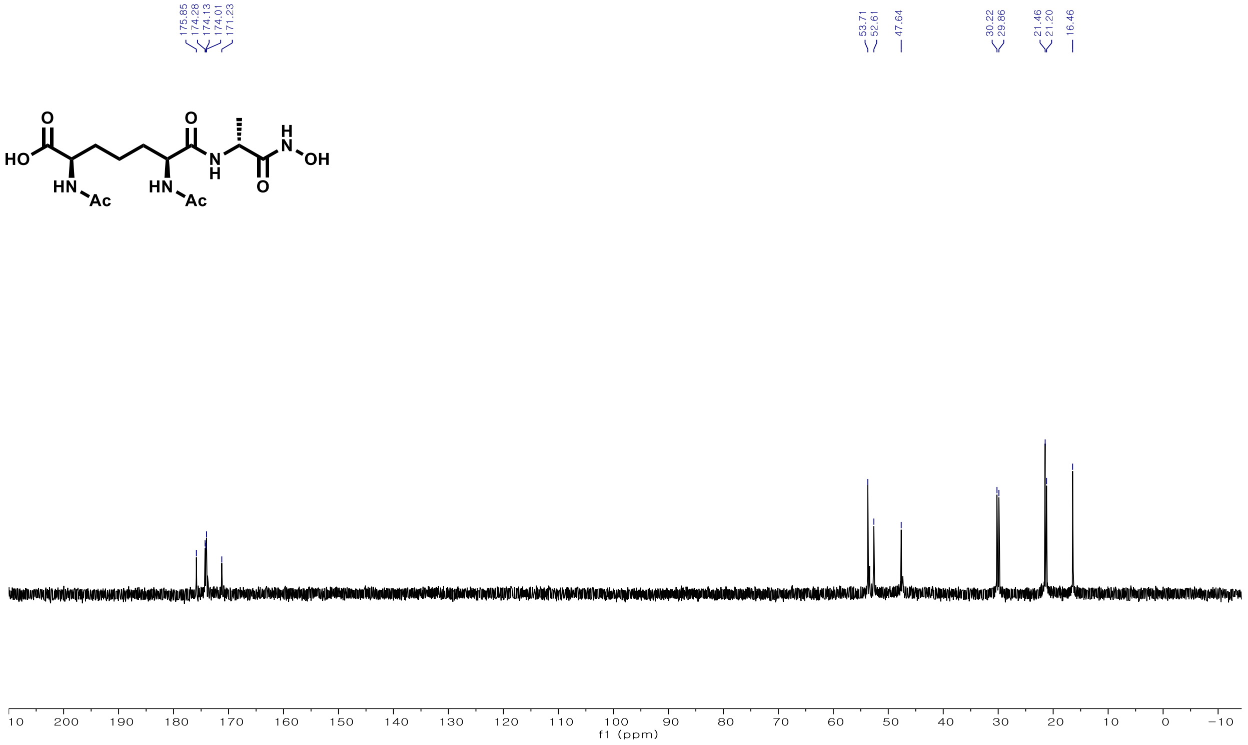

Compound 13

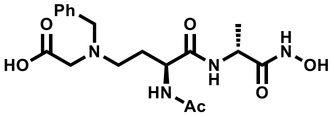
Ph
HO
O
N
O
N
H
HN
Ac
H
N
OH
O

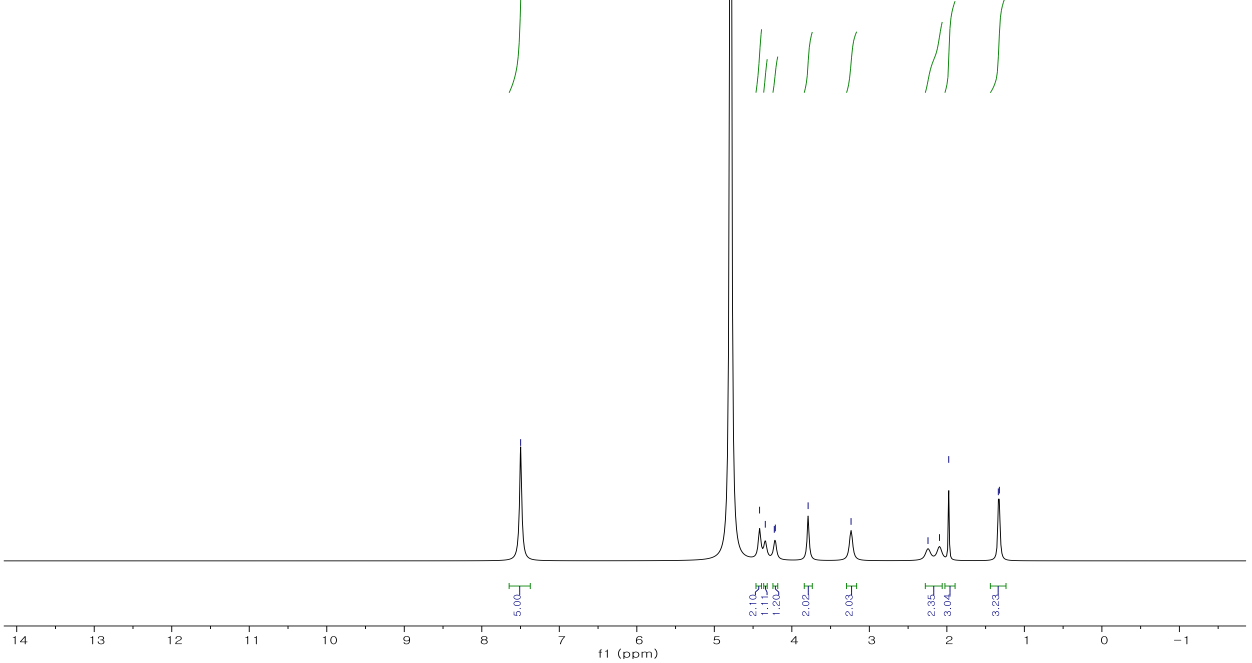
7.50
4.79
4.42
4.34
4.23
4.21
3.79
3.24
2.24
2.10
1.98
1.34
1.32
5.00
2.10
1.11
1.20
2.02
2.03
2.35
3.04
3.23
14 13 12 11 10 9 8 7 6 5 4 3 2 1 0 −1
f1 (ppm)

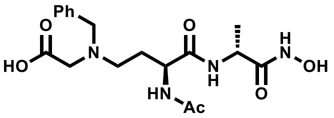
Ph
HO
O
N
O
N
H
HN
Ac
H
N
OH
O

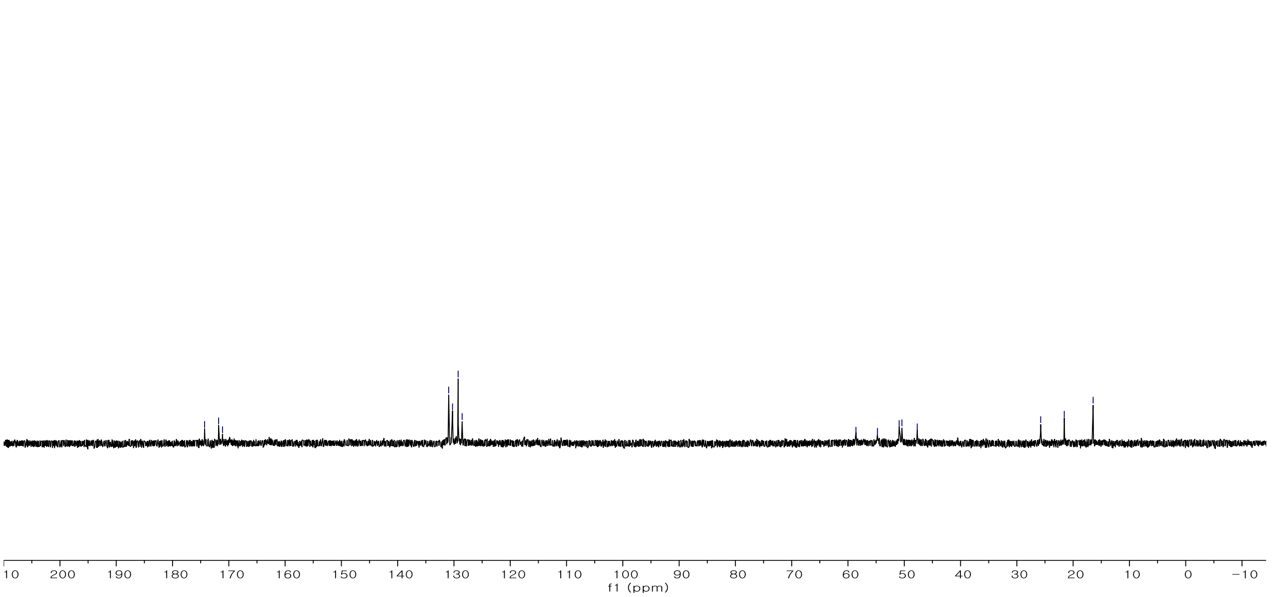
174.31
171.81
171.12
130.94
130.26
129.26
128.55
58.58
54.76
50.91
50.41
47.70
25.76
21.58
16.44
10 200 190 180 170 160 150 140 130 120 110 100 90 80 70 60 50 40 30 20 10 0 −10
f1 (ppm)

Compound 14

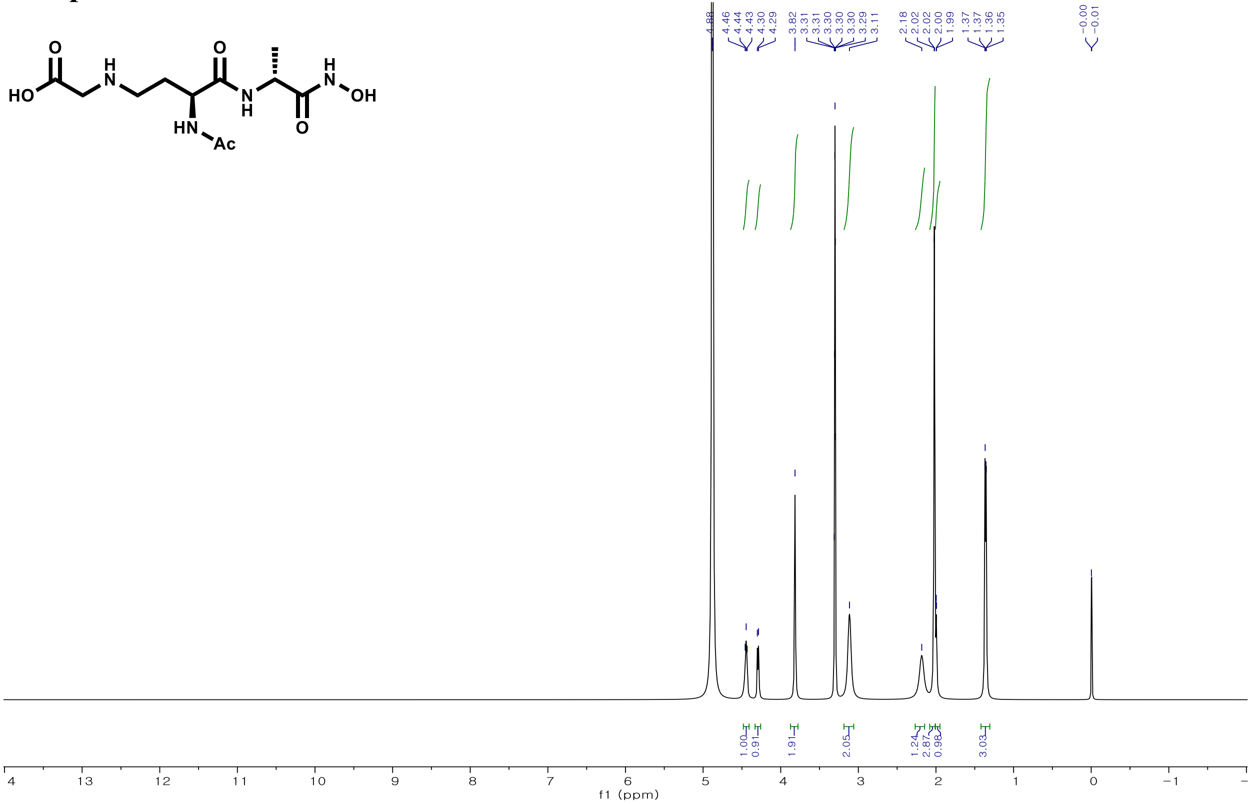
4.88
4.46
4.44
4.43
4.30
4.29
3.82
3.31
3.31
3.30
3.30
3.30
3.29
3.11
2.18
2.02
2.02
2.00
1.99
1.37
1.37
1.36
1.35
-0.00
-0.01
1.00
0.91
1.91
2.05
1.24
2.87
0.98
3.03
f1 (ppm)

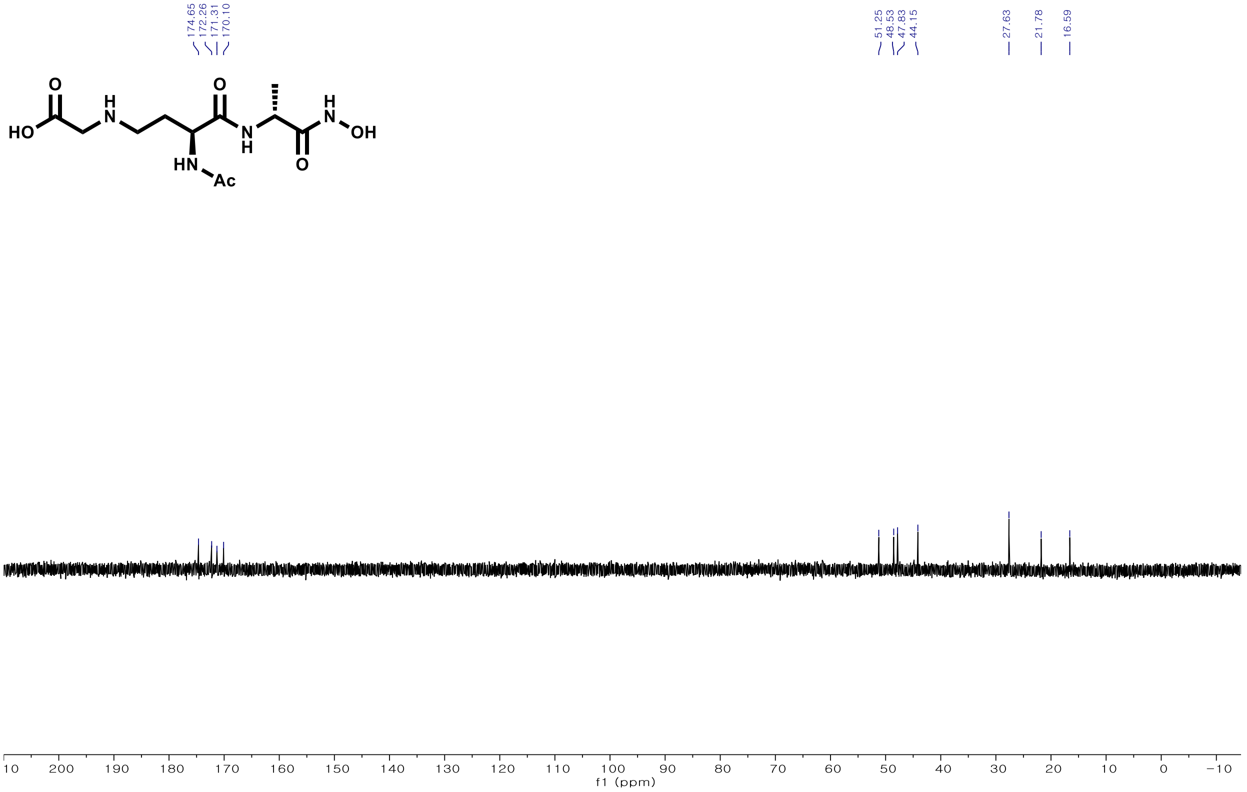
174.65
172.26
171.31
170.10
51.25
48.53
47.83
44.15
27.63
21.78
16.59
f1 (ppm)

Compound 19

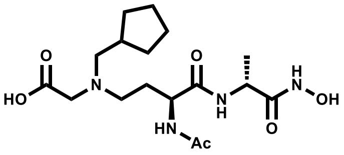

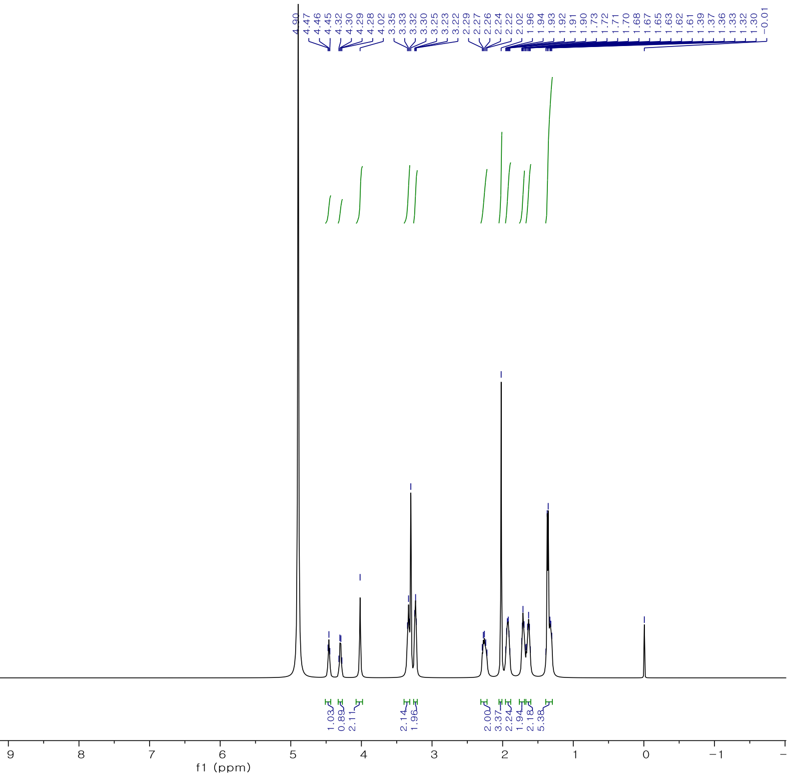

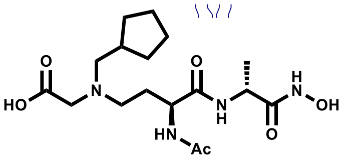

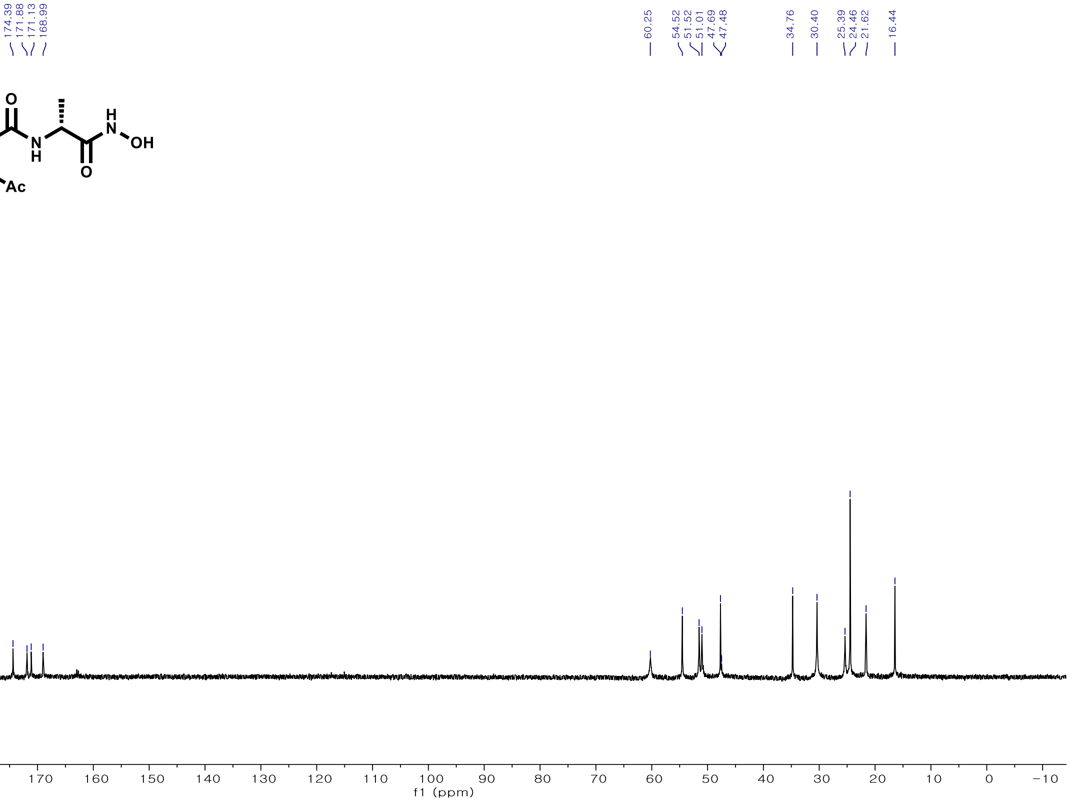

Compound 22

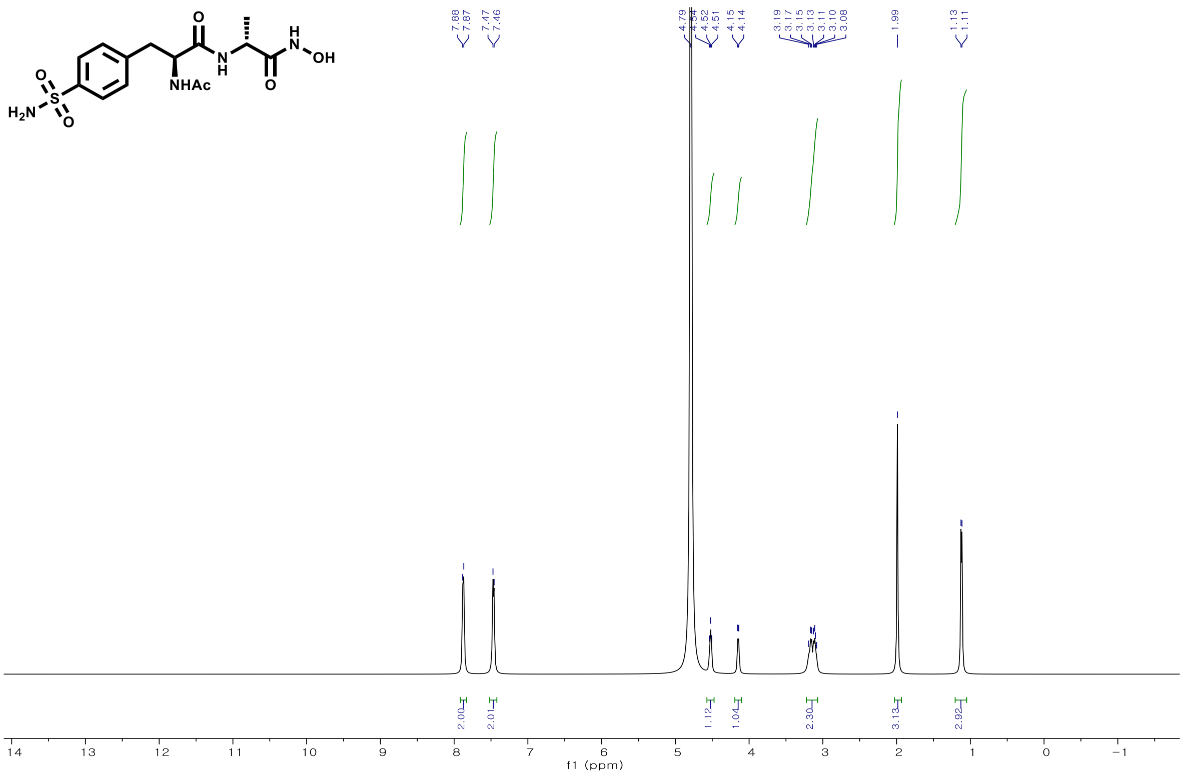
7.88 7.87 7.47 7.46 4.79 4.54 4.52 4.51 4.15 4.14 3.19 3.17 3.15 3.13 3.11 3.10 3.08 1.99 1.13 1.11
2.00 2.01 1.12 1.04 2.30 3.13 2.92
NHAc
OH
H_2N
f1 (ppm)

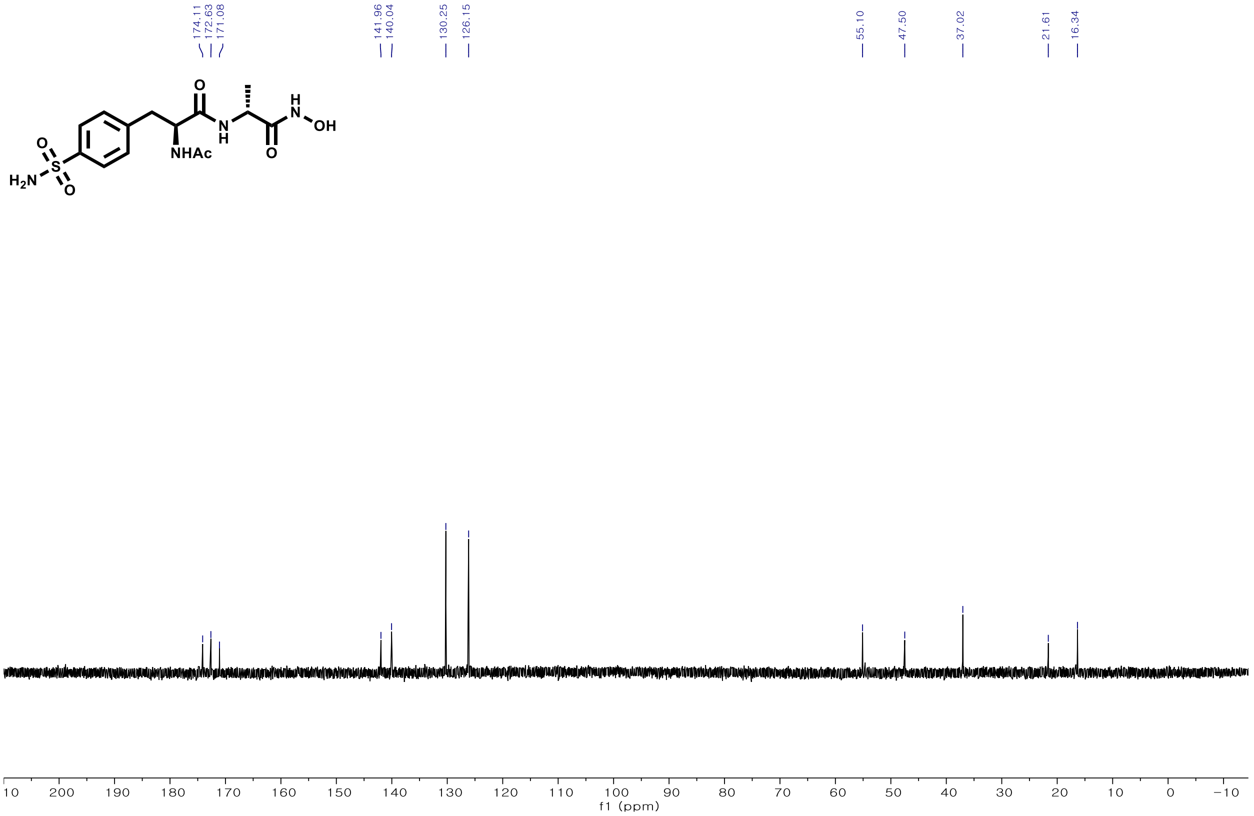
174.11 172.63 171.08 141.96 140.04 130.25 126.15 55.10 47.50 37.02 21.61 16.34
NHAc
OH
H_2N
f1 (ppm)

Compound 25

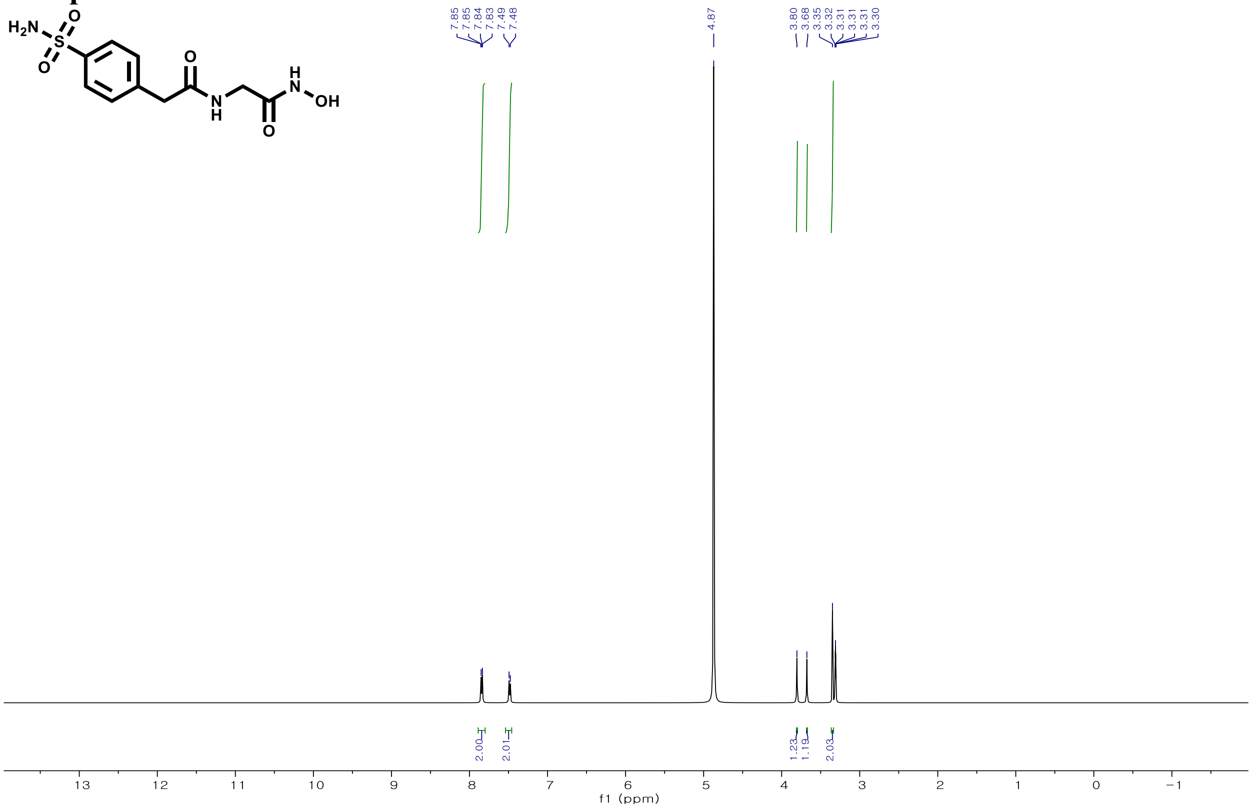
7.85
7.85
7.84
7.83
7.49
7.48
4.87
3.80
3.68
3.35
3.32
3.31
3.31
3.31
3.30
2.00
2.01
1.23
1.19
2.03
f1 (ppm)

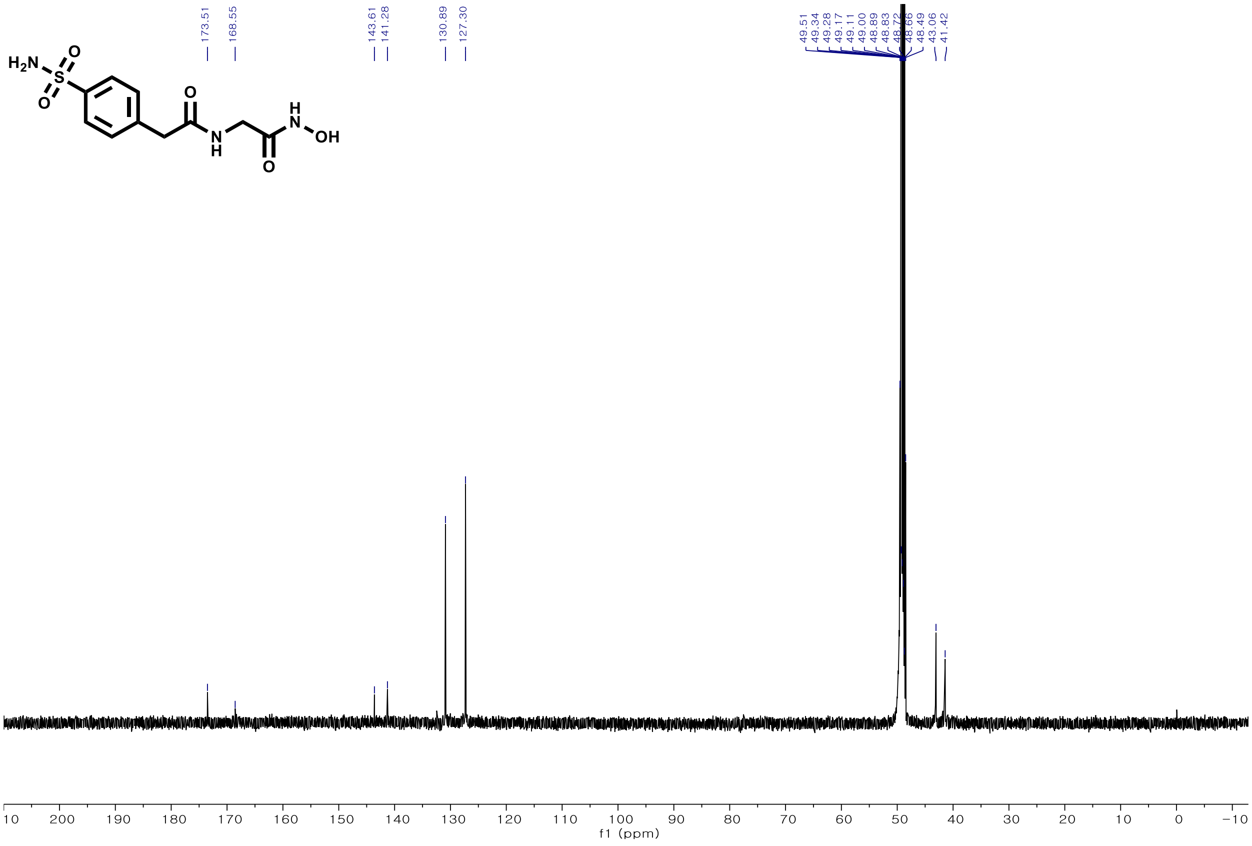
173.51
168.55
143.61
141.28
130.89
127.30
49.51
49.34
49.28
49.17
49.11
49.00
48.89
48.83
48.72
48.66
48.49
43.06
41.42
f1 (ppm)

Compound 30

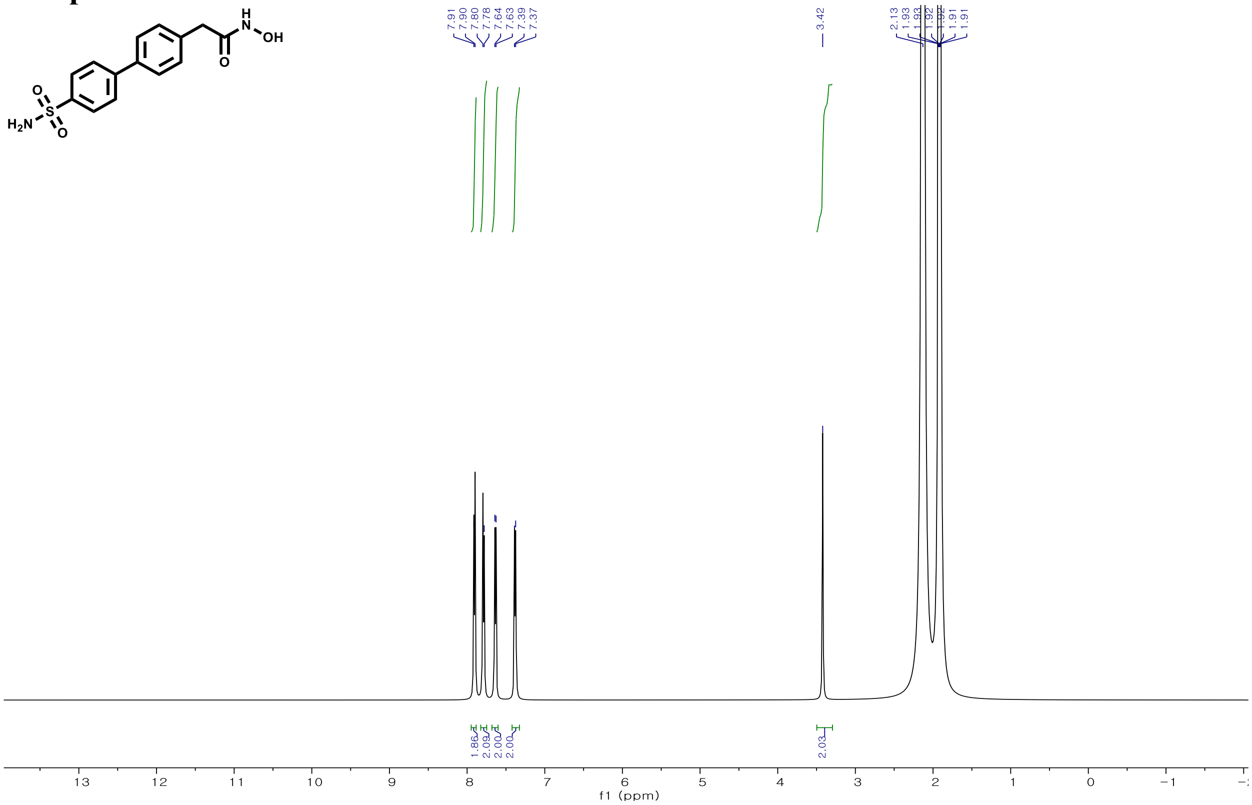

7.91
7.90
7.80
7.78
7.64
7.63
7.39
7.37
3.42
2.13
1.93
1.93
1.92
1.92
1.91
1.91
1.86
2.09
2.00
2.00
2.03
f1 (ppm)

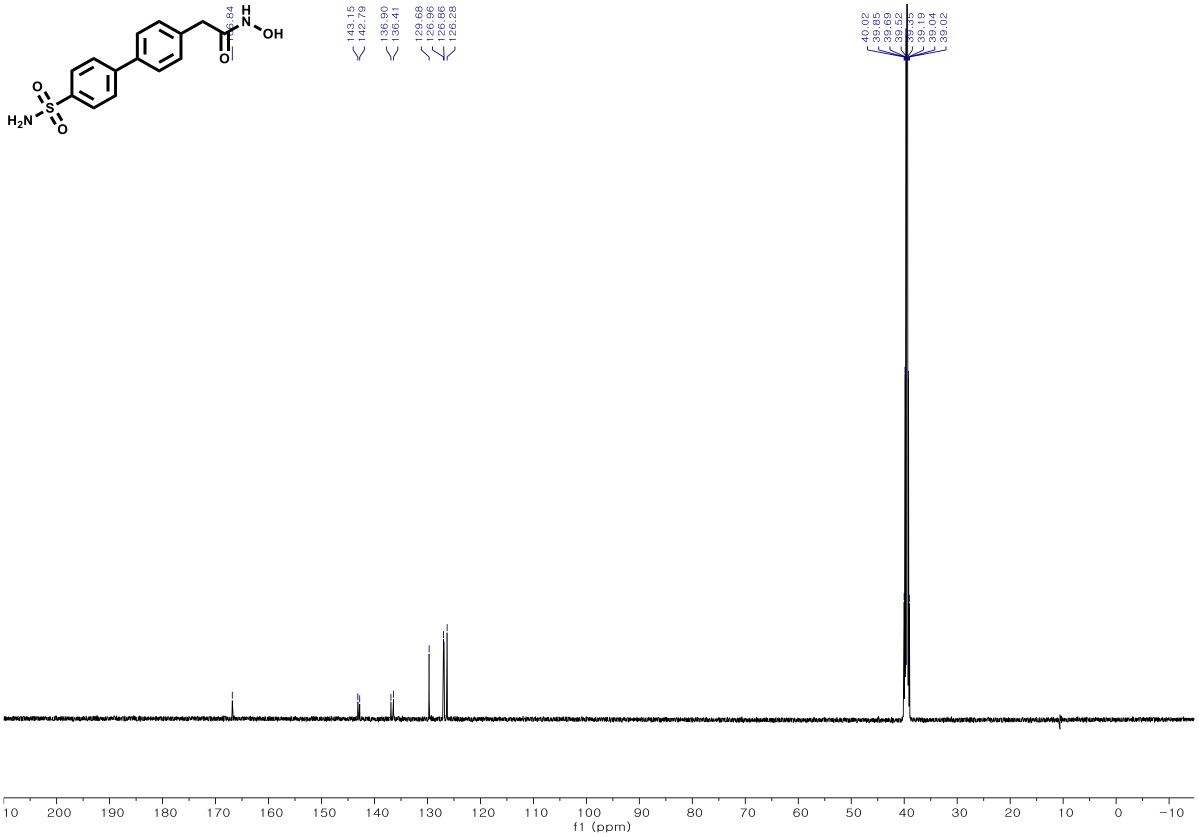

143.15
142.79
136.90
136.41
129.68
126.96
126.86
126.28
40.02
39.85
39.69
39.52
39.35
39.19
39.04
39.02
f1 (ppm)

Compound 35

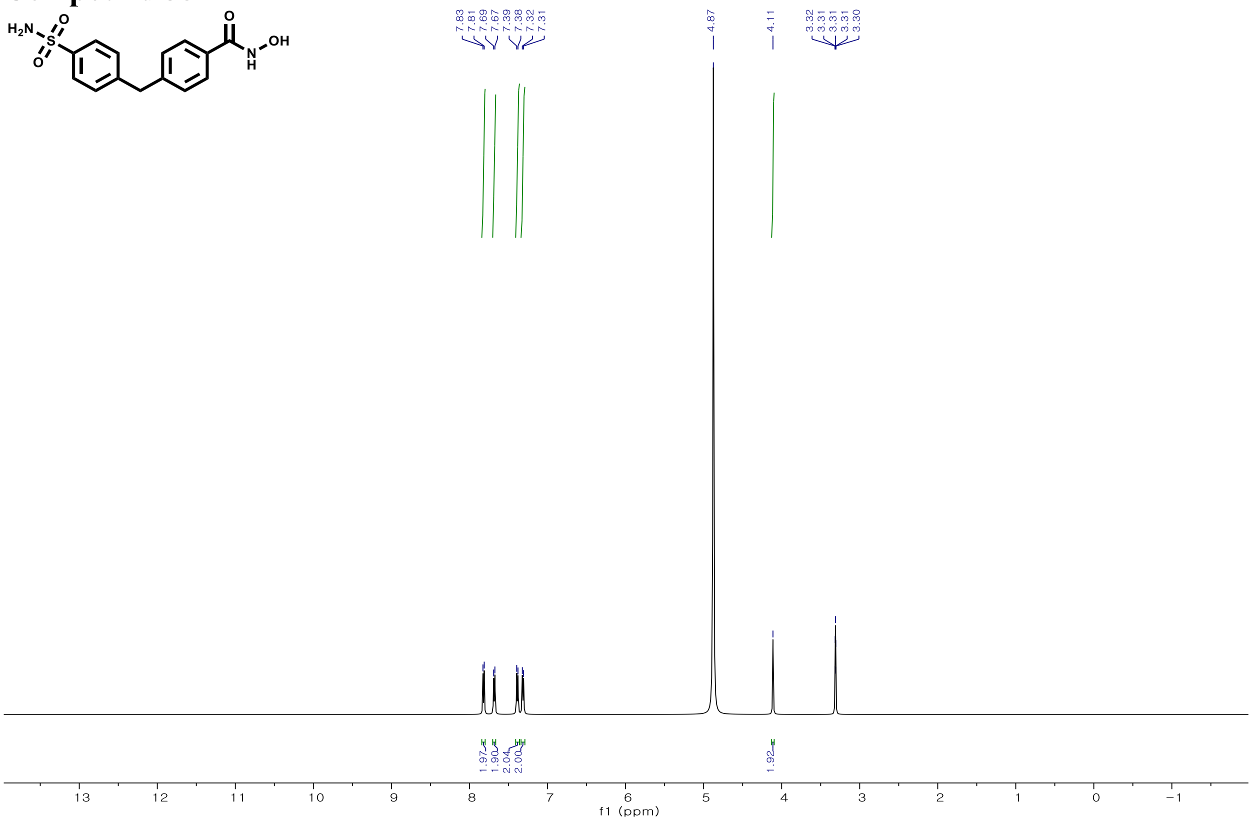

H2N
S
O
O
N
H
OH
7.83
7.81
7.69
7.67
7.39
7.38
7.32
7.31
4.87
4.11
3.32
3.31
3.31
3.31
3.30
1.97
1.90
2.04
2.00
1.92
13 12 11 10 9 8 7 6 5 4 3 2 1 0 -1
f1 (ppm)

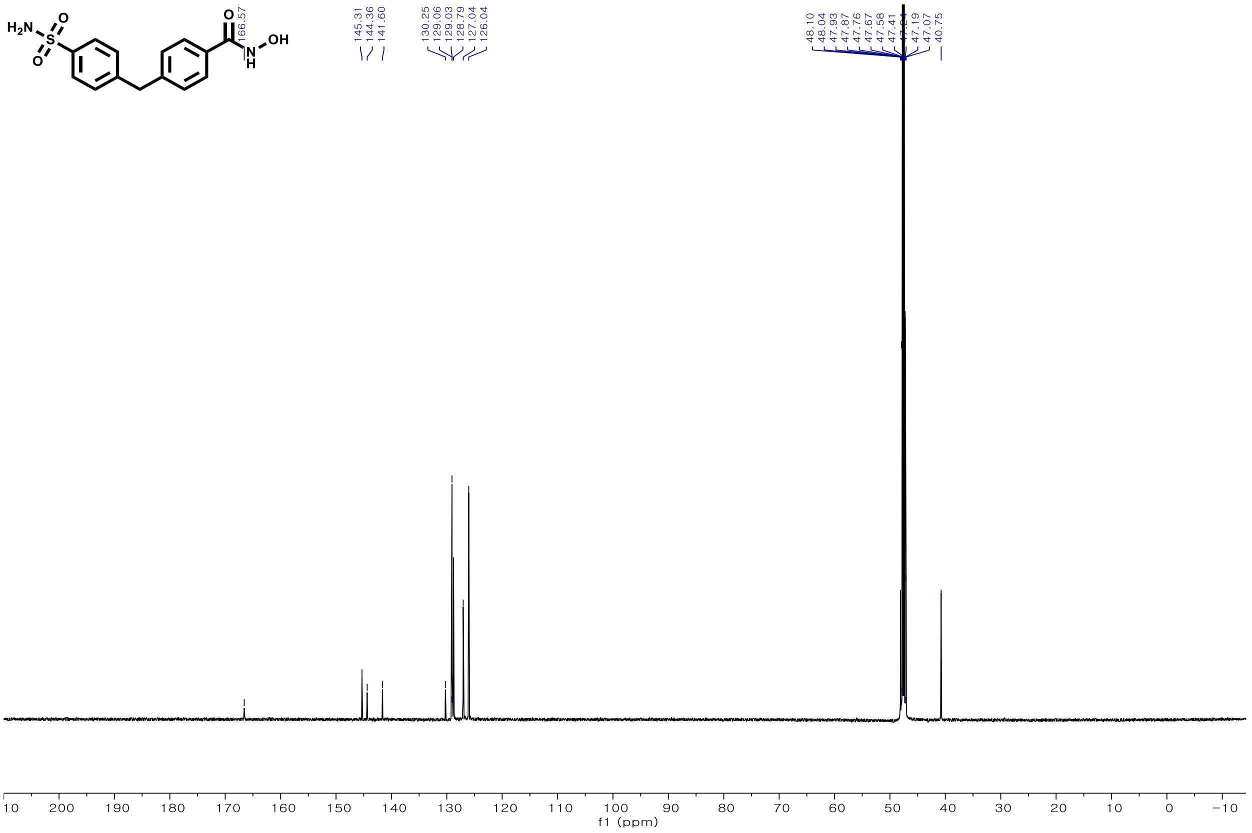

H2N
S
O
O
N
H
OH
166.57
145.31
144.36
141.60
130.25
129.06
129.03
128.79
127.04
126.04
48.10
48.04
47.93
47.87
47.76
47.67
47.58
47.41
47.19
47.07
40.75
10 200 190 180 170 160 150 140 130 120 110 100 90 80 70 60 50 40 30 20 10 0 -10
f1 (ppm)

HPLC Spectra of compound

FRET-labeled peptide substrate

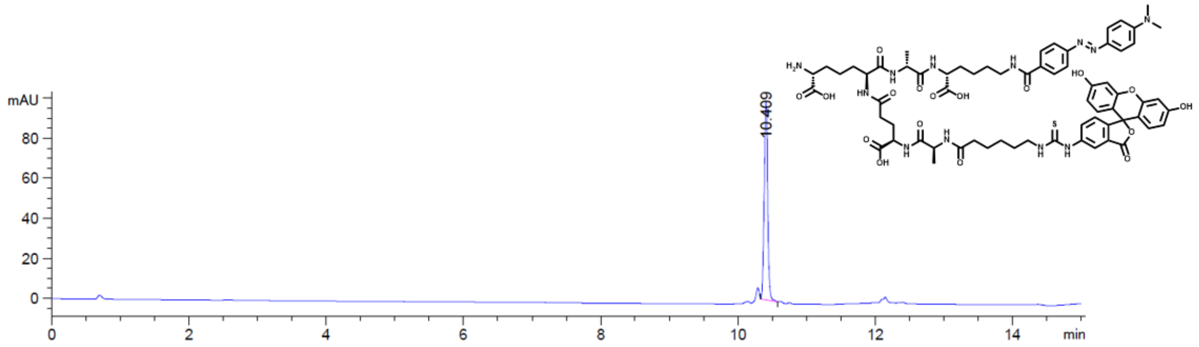

HPLC chromatogram of KL-L9P

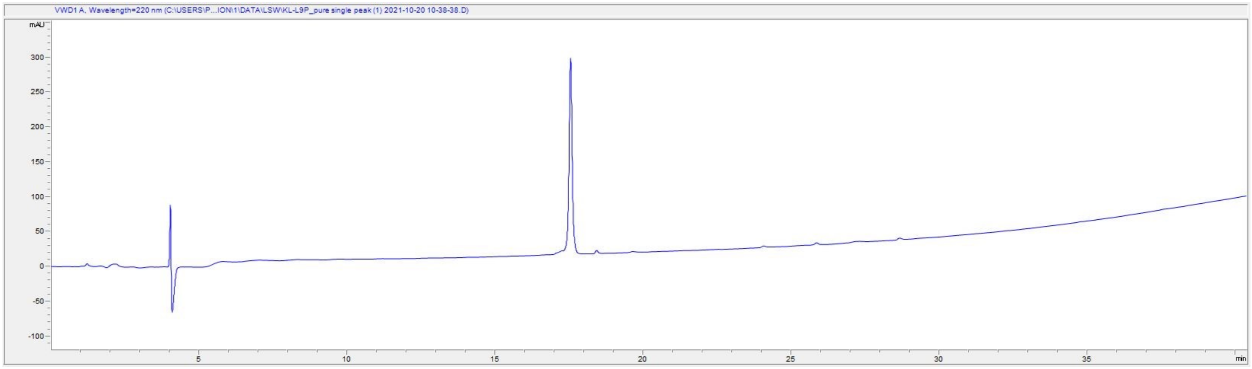

A Zorbax 300SB-C18 (5 µm, 9.4 × 250 mm) column was used as the stationary phase. For the mobile phase, buffer A (water with 0.1% v/v TFA) and buffer B (acetonitrile with 0.1% v/v TFA) were used with a linear gradient from 5% to 100% of buffer B over 40 min.

MALDI-TOF MS spectrum of KL-L9P

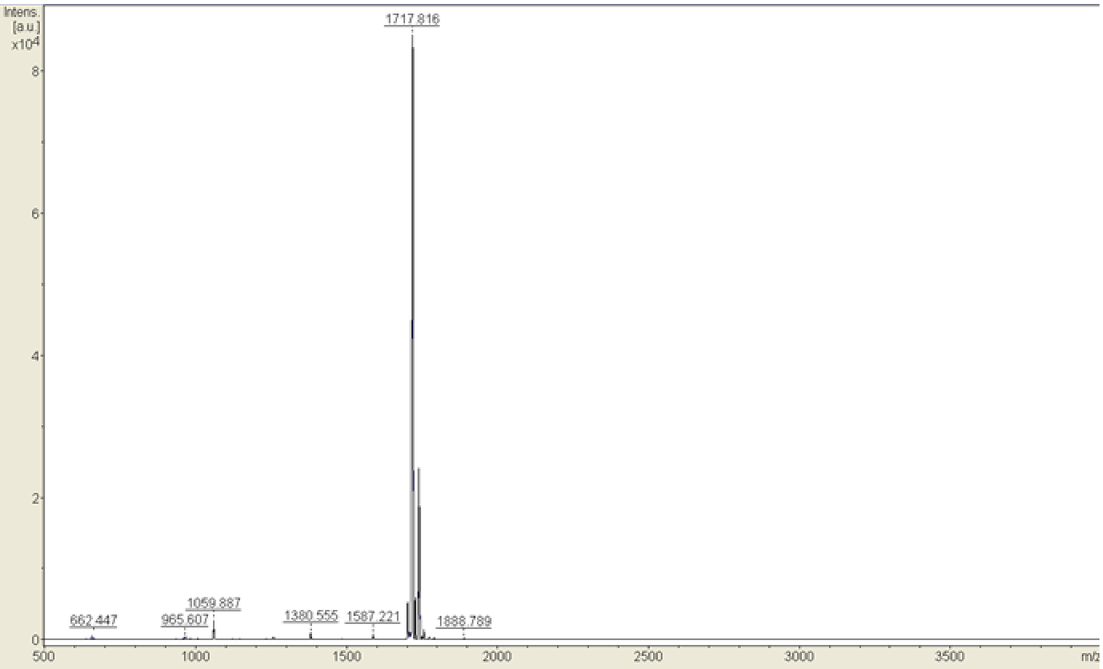

The MALDI-TOF MS spectrum of KL-L9P. MS [M+H]+ : 1717.25 (calcd.), 1717.8 (found).

Supplementary Figures

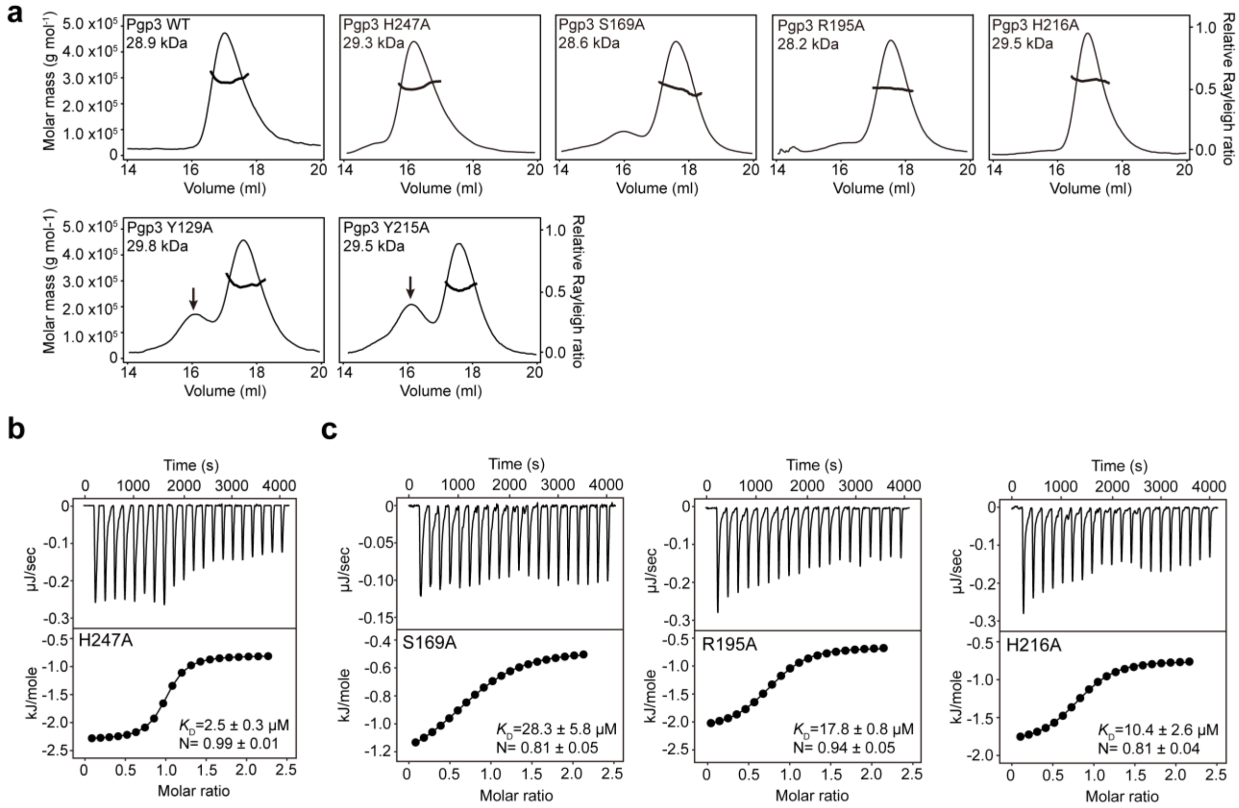

Supplementary Figure 1. ITC assay of Pgp3 variants with BMK-S101. a. The molecular weight of Pgp3 WT and variants (H247A, S169A, R195A, H216A, Y129A, and Y215A) measured by SEC-MALS. The thick line represents measured molecular mass. Since Pgp3 variants in Y-Y motif (Y129A and Y215A) affected the protein folding, these variants were excluded for following ITC assay. **b-c.** The ITC fitting results of Pgp3 variants (**b** H247A or **c** S169A, R195A, and H216A) with BMK-S101 are shown. The thermodynamic data were collected from titration of BMK-S101 into Pgp3 proteins, and the parameters were calculated by fitting to a single-binding-site model. The resulting K_D values are given in Fig. 2d as well.

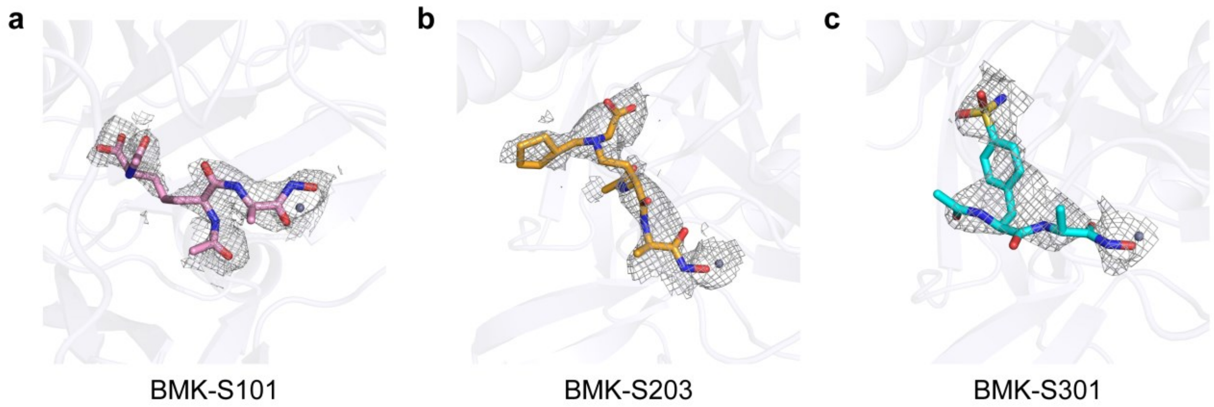

Supplementary Figure 2. Electron-density maps of inhibitors.

a-c. $2F_O–F_C$ electron density maps (0.8 σ) of representative inhibitors **a** BMK-S101 (light pink), **b** BMK-S203 (bright orange), and **c** BMK-S301 (cyan).

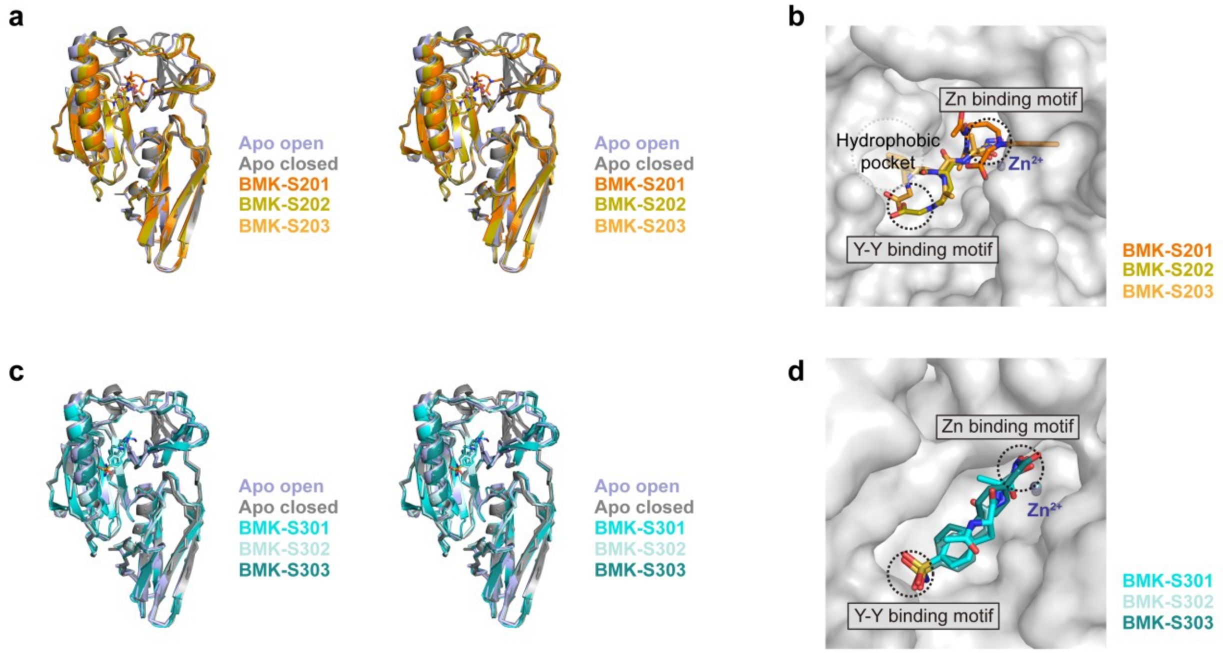

Supplementary Figure 3. Overall comparison of Pgp3 structures in complex with inhibitors. a. Stereo view of the superimposition between Pgp3 WT (open form, light blue, PDB ID: 6JMX), Pgp3 WT (closed form, grey, PDB ID: 6JMY), Pgp3 H247A with BMK-S201 (orange), Pgp3 H247A with BMK-S202 (olive), and Pgp3 H247A with BMK-S203 (bright orange). **b.** Surface representation of the Pgp3 in complex with BMK-S201, BMK-S202, and BMK-S203. Representative motifs are shown in dashed circles, and inhibitors are colored in the same color with Supplementary Fig. 3a. **c.** Stereo view of the superimposition between Pgp3 WT (open form, light blue, PDB ID: 6JMX), Pgp3 WT (closed form, grey, PDB ID: 6JMY), Pgp3 H247A with BMK-S301 (cyan), Pgp3 H247A with BMK-S302 (light teal), and Pgp3 H247A with BMK-S303 (deep teal). **d.** Surface representation of the Pgp3 in complex with BMK-S301, BMK-S302, and BMK-S303. Representative motifs are shown in dashed circles, and inhibitors are colored in the same color with Supplementary Fig. 3c.

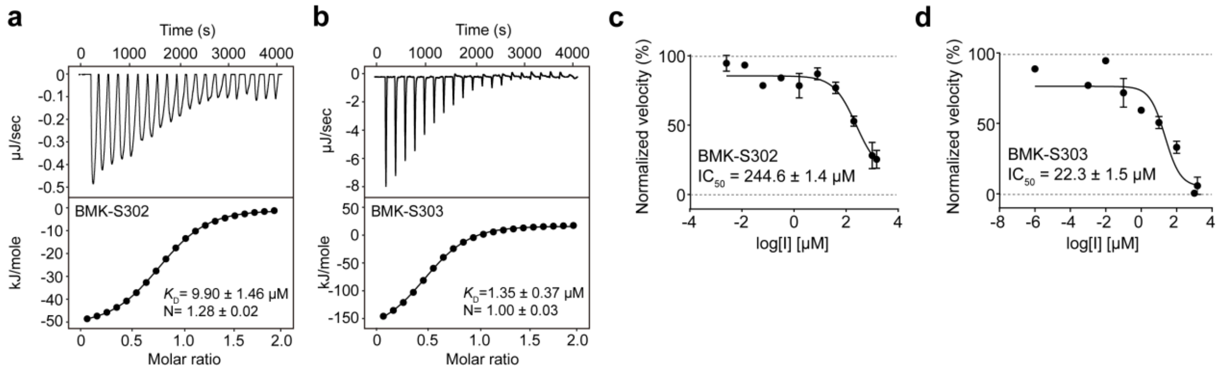

Supplementary Figure 4. Functional studies on Pgp3 in complex with BMK-S301 derivatives. a-b. The ITC fitting results of Pgp3 with **a** BMK-S302 and **b** BMK-S303. **c-d.** IC_{50} fitting curves for **c** BMK-S302 and **d** BMK-S303. The data are presented as means ± S.E.M. (n=3).

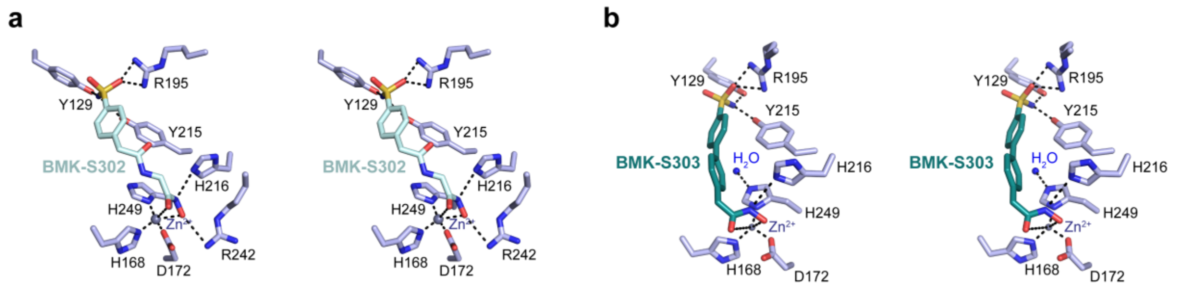

Supplementary Figure 5. Crystal structures of Pgp3 H247A in complex with BMK-S301 derivatives. a-b. Detailed interactions in Pgp3 with BMK-S301 derivatives are shown. Stereo diagrams of Pgp3 H247A active sites bound with **a** BMK-S302 (light teal), and **b** BMK-S303 (deep teal). The coordination of Zn^{2+} and hydrogen bonds are represented by black dashed lines. The Zn^{2+} ion and water molecules are shown in blue and slate gray spheres, respectively.

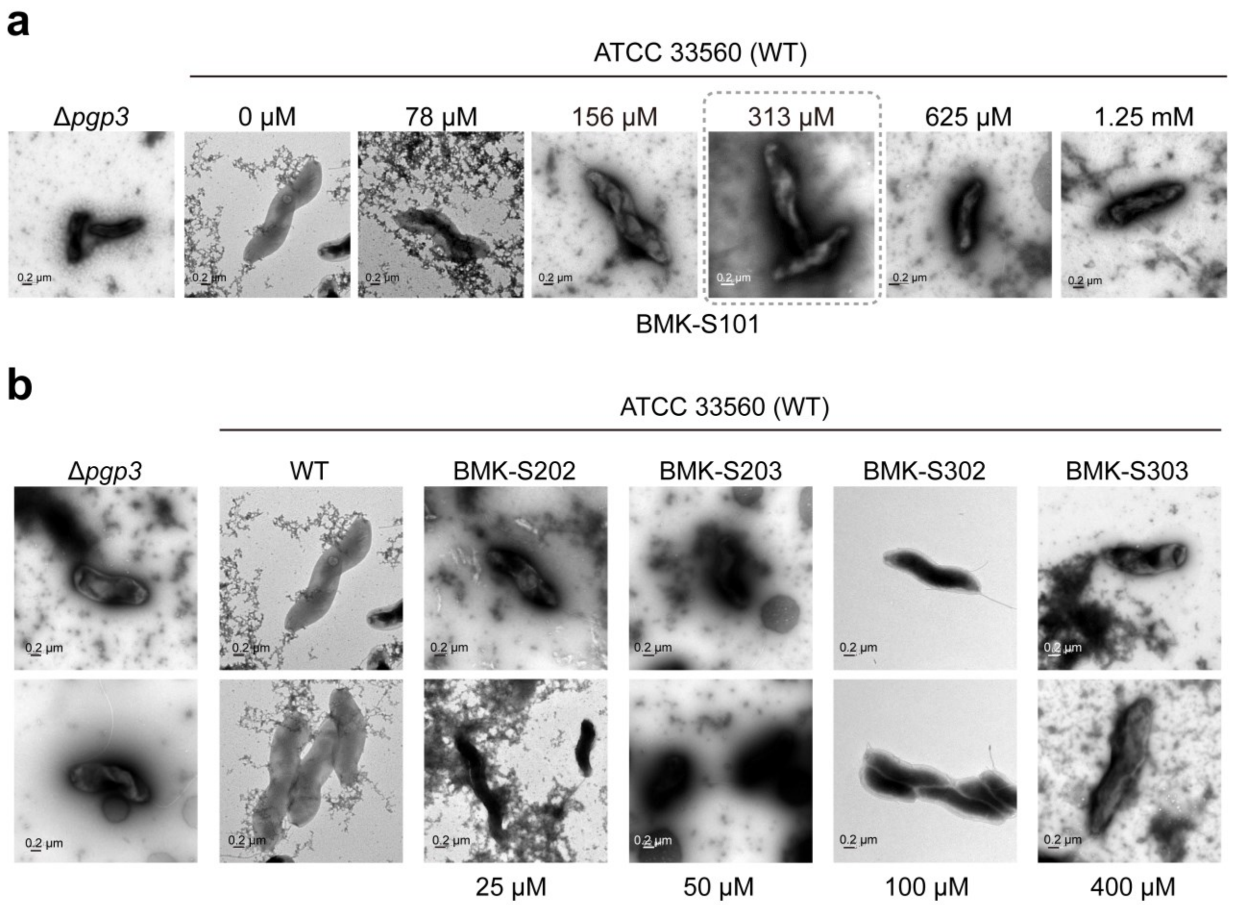

Supplementary Figure 6. TEM analyses of *C. jejuni* strains upon inhibitor treatment.

a-b. *C. jejuni* WT (ATCC 33560) and Δ*pgp3* were negatively stained with 2% (w/v) uranyl acetate and then observed by TEM. For inhibitor treated sample, each concentration of inhibitors was incubated at 37 °C for 24 h before staining. **a.** TEM analysis of *C. jejuni* strains treated with BMK-S101. The minimum concentration of inhibitors which shows changes in the cell morphology is highlighted in gray dashed box. **b.** The representative images of *C. jejuni* at the minimum concentration of inhibitors that shows changes in the cell morphology are given. Scale bars (lower left) represent 0.2 μm.

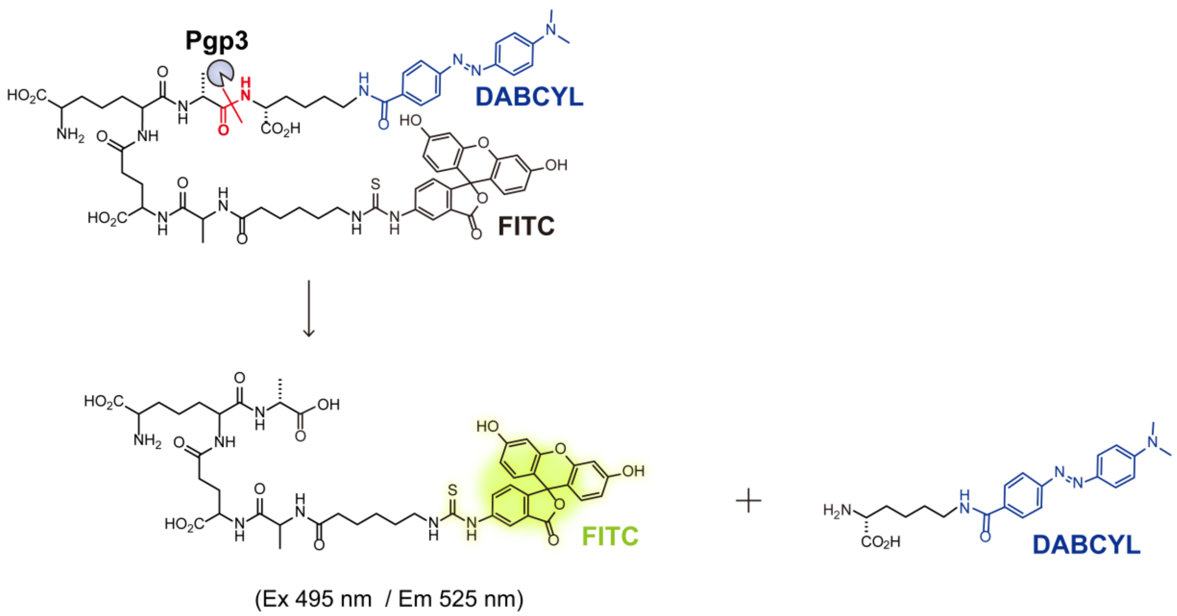

Supplementary Figure 7. Reaction scheme of the pentapeptide-derived substrate used in Pgp3 kinetic studies. DABCYL/FITC pair was used for fluorescence signal (excitation 495 nm, emission 525 nm).

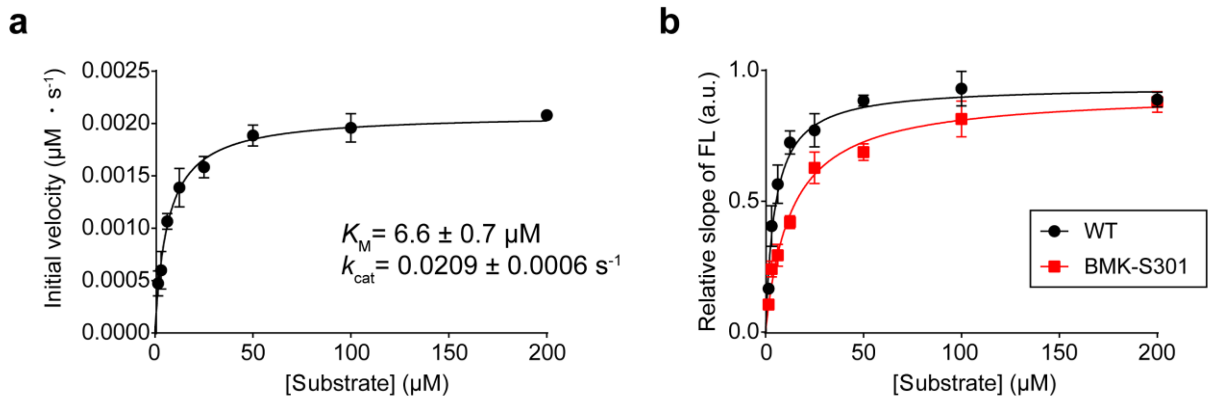

Supplementary Figure 8. Determination of kinetic parameters of Pgp3 and its inhibitor. a. K_M and k_{cat} values were determined by plotting initial rates versus substrate concentrations for the hydrolysis of the pentapeptide-derived substrate by Pgp3. Data have been fit to the Michaelis-Menten equation. **b.** Comparison between enzyme kinetics of Pgp3 with/without the inhibitor. Data have been fit to the Michaelis-Menten equation. 100 μM of BMK-S301 was used as a representative inhibitor for Pgp3. Error bars represent the means and SEM from three independent experiments.

Supplementary Table 1. Statistics for data collection and refinement.

Data set	H247A BMK-S101 bound	H247A BMK-S201 bound	H247A BMK-S202 bound	H247A BMK-S203 bound
PDB codes	7E60	7E61	7E64	7E63
A. Data collection				
X-ray source	Spring8-BL26	PLS-5C	PLS-5C	PLS-5C
X-ray wavelength (Å)	0.9999	0.9796	1.0000	0.9795
Space group	$P6_1$	$P6_1$	$P6_1$	$P2_12_12_1$
Unit cell length (*a*, *b*, *c*, Å)	115.5, 115.5, 55.9	115.2, 115.2, 55.3	115.8, 115.8, 57.7	56.9, 90.7, 100.9
Unit cell angle (α, β, γ, °)	90, 90, 120	90, 90, 120	90, 90, 120	90, 90, 90
Resolution range (Å)	50.0–2.20 (2.32–2.20)[a]	50.0–1.80 (1.90–1.80)[a]	30.0–2.90 (3.06-2.90)[a]	67.48–2.40 (2.44–2.40)[a]
Total / unique reflections	376,216 / 42,385	805,928 / 76,068	103,497 / 19,252	211,553 / 20,556
Completeness (%)	100.0 (99.8)[a]	100.0 (100.0)[a]	99.6 (98.4)[a]	96.1 (93.0)[a]
Average $I/\sigma(I)$	15.67 (2.0)[a]	29.5 (4.3)[a]	9.73 (0.5)[a]	13.2 (1.3)[a]
R_{merge}[b] (%)	11.3 (101.1)[a]	5.1 (56.8)[a]	12.7 (87.5)[a]	17.1 (95.6)[a]
B. Model refinement statistics				
Resolution range (Å)	37.3–2.24	39.9–1.80	30.0–2.90	67.48–2.38
R_{work} / R_{free}[c] (%)	18.9 / 23.4	15.5 / 18.7	23.2 / 23.9	19.7 / 26.5
Monomers per asymmetric unit	1	1	1	2
Number of non-hydrogen atoms / average *B*-factor ($Å^2$)				
Protein	2,014/ 30.4	2,023 / 33.2	2,014 / 65.3	3,994 / 45.1
Water oxygen	148 / 34.3	290 / 43.1	0 / 0	195 / 41.0
Zn^{2+}	1 / 31.0	1 / 23.5	1 / 67.0	2 / 40.5
Inhibitor	25 / 68.2	28 / 64.1	22 / 71.7	27 / 81.0
R.m.s. deviations from ideal geometry				
Bond lengths (Å) / bond angles (°)	0.010 / 1.78	0.026 / 2.44	0.004 / 0.91	0.011 / 1.48
Protein-geometry analysis				
Ramachandran favored (%)	95.3	97.2	93.7	92.9
Ramachandran allowed (%)	4.4	2.4	4.4	5.1
Ramachandran outliers (%)	0.3	0.4	1.9	2.0

Supplementary Table 1. *contd.*

Data set	H247A BMK-S301 bound	H247A BMK-S302 bound	H247A BMK-S303 bound
PDB codes	7E65	7E66	7E67
A. Data collection			
X-ray source	PLS-5C	PLS-5C	PLS-5C
X-ray wavelength (Å)	0.9795	1.0000	1.0000
Space group	$P2_12_12_1$	$P6_1$	$P6_1$
Unit cell length (*a*, *b*, *c*, Å)	56.4, 91.0, 100.9	115.6, 115.6, 57.1	115.1, 115.1, 56.6
Unit cell angle (α, β, γ, °)	90, 90, 90	90, 90, 120	90, 90, 120
Resolution range (Å)	70.0–2.65 (2.70–2.65)[a]	50.0–2.85 (3.00–2.85)[a]	50.0–2.85 (3.02–2.85)[a]
Total / unique reflections	191,812 / 15,653	106,009 / 19,937	214,141 / 19,590
Completeness (%)	99.8 (98.4)[a]	99.7 (99.0)[a]	100 (99.9)[a]
Average $I/\sigma(I)$	15.1 (2.5)[a]	8.6 (1.4)[a]	11.3 (3.3)[a]
R_{merge}[b] (%)	18.6 (95.2)[a]	14.3 (89.7)[a]	21.2 (82.4)[a]
B. Model refinement statistics			
Resolution range (Å)	67.6–2.65	28.9–2.90	40.4–2.85
R_{work} / R_{free}[c] (%)	20.7 / 27.8	21.3 / 24.1	22.3 / 27.8
Monomers per asymmetric unit	2	1	1
Number of non-hydrogen atoms / average *B*-factor ($Å^2$)			
Protein	3,994 / 45.8	2,014 / 70.4	2,014 / 47.0
Water oxygen	129 / 32.2	7 / 53.2	20 / 31.3
Zn^{2+}	2 / 45.6	1 / 62.3	1 / 45.3
Inhibitor	24 / 97.7	19 / 92.3	21 / 75.9
R.m.s. deviations from ideal geometry			
Bond lengths (Å) / bond angles (°)	0.010 / 1.43	0.004 / 0.99	0.004 / 0.98
Protein-geometry analysis			
Ramachandran favored (%)	96.1	94.5	92.5
Ramachandran allowed (%)	3.9	5.1	6.7
Ramachandran outliers (%)	0	0.4	0.8

Footnotes for Supplementary Table 1

[a]Values in parentheses refer to the highest resolution shell.

[b] $R_{merge} = \Sigma_{hkl}\Sigma_i \mid I_i(hkl) - \langle I(hkl)\rangle \mid / \Sigma_{hkl}\Sigma_i I_i(hkl)_i$, where $I(hkl)$ is the intensity of reflection *hkl*, Σ_{hkl} is the sum over all reflections, and Σ_i is the sum over i measurements of reflection *hkl*.

[c]$R = \Sigma_{hkl} \mid |F_{obs}| - |F_{calc}| \mid / \Sigma_{hkl} |F_{obs}|$, where R_{free} was calculated for a randomly chosen 5% of reflections, which were not used for structure refinement and R_{work} was calculated for the remaining.

Supplementary Table 2. Primers for site-directed mutagenesis.

Primers	Sequence (5′-3′)
Pgp3-NdeI-F	GCGCTCATATGATGAAAGCCTTATGGCTTTTTTTAAG
Pgp3-XhoI-R	GCGCTCTCGAGTCATTGAAAAATAGCATTAAATTTGGAT
Pgp3-Y129A-F	TAAAAGAAGCCAATGCGATTGCTAGTTCTTATACTCCAAAAGC
Pgp3-Y129A-R	GCTTTTGGAGTATAAGAACTAGCAATCGCATTGGCTTCTTTTA
Pgp3-S169A-F	AAAAAGTAGCAAGTTATCATGCTGGAACGGACTTTAGAGCCGC
Pgp3-S169A-R	GCGGCTCTAAAGTCCGTTCCAGCATGATAACTTGCTACTTTTT
Pgp3-R195A-F	TAGTAAAAATTGCAAAAGATGCTTATTTCGCAGGAAATTCAGT
Pgp3-R195A-R	ACTGAATTTCCTGCGAAATAAGCATCTTTTGCAATTTTTACTA
Pgp3-Y215A-F	TTGGAATTTATTCACAATATGCTCATCTTTCTAAAATCGATGT
Pgp3-Y215A-R	ACATCGATTTTAGAAAGATGAGCATATTGTGAATAAATTCCAA
Pgp3-H216A-F	GAATTTATTCACAATATTATGCTCTTTCTAAAATCGATGTTAA
Pgp3-H216A-R	TTAACATCGATTTTAGAAAGAGCATAATATTGTGAATAAATTC
Pgp3-H247A-F	GTGGTAGGGTAAGTGGGCCGGCTTTGCATTTTGGAATTTTAGC
Pgp3-H247A-R	GCTAAAATTCCAAAATGCAAAGCCGGCCCACTTACCCTACCAC

Supplementary Movie 1. Targeted MD movie depicting dynamic interaction between Pgp3 and BMK-S101

Supplementary Movie 2. Targeted MD movie depicting dynamic interaction between Pgp3 and BMK-S203

Supplementary Movie 3. Targeted MD movie depicting dynamic interaction between Pgp3 and BMK-S301

Supplementary Data 1. AMBER topology and coordinate files of the molecular-dynamics simulations used in this study.

Supplementary Data 2. Source data for figure 5.

Supplementary Reference

1. Hermant, P. et al. Controlling plasma stability of hydroxamic acids: A medchem toolbox. *J. Med. Chem.* **60**, 9067−9089 (2017).
