# Supplementary material for: Structure-based inhibitor design for reshaping bacterial morphology
Source: Commun Biol. 2022 Apr 28;5:395. doi: 10.1038/s42003-022-03355-3 (PMC9050674; doi:10.1038/s42003-022-03355-3)
Supplement: Supplementary file 2 — Description of Additional Supplementary Files [file 42003_2022_3355_MOESM2_ESM.pdf]

Description of Additional Supplementary Files

File name: Supplementary Data 1
Description: AMBER topology and coordinate files of the molecular-dynamics simulations used in this study.

File name: Supplementary Data 2
Description: Source data for figure 5.

File name: Supplementary Movie 1
Description: Targeted MD movie depicting dynamic interaction between Pgp3 and BMK-S101

File name: Supplementary Movie 2
Description: Targeted MD movie depicting dynamic interaction between Pgp3 and BMK-S203

File name: Supplementary Movie 3
Description: Targeted MD movie depicting dynamic interaction between Pgp3 and BMK-S301
